# An Integrated Approach to Reconstructing Genome-Scale Transcriptional Regulatory Networks

**DOI:** 10.1371/journal.pcbi.1004103

**Published:** 2015-02-27

**Authors:** Saheed Imam, Daniel R. Noguera, Timothy J. Donohue

**Affiliations:** 1 Program in Cellular and Molecular Biology, University of Wisconsin—Madison, Madison, Wisconsin, United States of America; 2 Department of Bacteriology, University of Wisconsin—Madison, Madison, Wisconsin, United States of America; 3 Wisconsin Energy Institute, University of Wisconsin—Madison, Madison, Wisconsin, United States of America; 4 Great Lakes Bioenergy Research Center, University of Wisconsin—Madison, Madison, Wisconsin, United States of America; 5 Department of Civil and Environmental Engineering, University of Wisconsin—Madison, Wisconsin, United States of America; Memorial Sloan-Kettering Cancer Center, UNITED STATES

## Abstract

Transcriptional regulatory networks (TRNs) program cells to dynamically alter their gene expression in response to changing internal or environmental conditions. In this study, we develop a novel workflow for generating large-scale TRN models that integrates comparative genomics data, global gene expression analyses, and intrinsic properties of transcription factors (TFs). An assessment of this workflow using benchmark datasets for the well-studied γ-proteobacterium *Escherichia coli* showed that it outperforms expression-based inference approaches, having a significantly larger area under the precision-recall curve. Further analysis indicated that this integrated workflow captures different aspects of the *E. coli* TRN than expression-based approaches, potentially making them highly complementary. We leveraged this new workflow and observations to build a large-scale TRN model for the α-Proteobacterium *Rhodobacter sphaeroides* that comprises 120 gene clusters, 1211 genes (including 93 TFs), 1858 predicted protein-DNA interactions and 76 DNA binding motifs. We found that ~67% of the predicted gene clusters in this TRN are enriched for functions ranging from photosynthesis or central carbon metabolism to environmental stress responses. We also found that members of many of the predicted gene clusters were consistent with prior knowledge in *R. sphaeroides* and/or other bacteria. Experimental validation of predictions from this *R. sphaeroides* TRN model showed that high precision and recall was also obtained for TFs involved in photosynthesis (PpsR), carbon metabolism (RSP_0489) and iron homeostasis (RSP_3341). In addition, this integrative approach enabled generation of TRNs with increased information content relative to *R. sphaeroides* TRN models built via other approaches. We also show how this approach can be used to simultaneously produce TRN models for each related organism used in the comparative genomics analysis. Our results highlight the advantages of integrating comparative genomics of closely related organisms with gene expression data to assemble large-scale TRN models with high-quality predictions.

## Author Summary

The ever growing amount of genomic data enables the assembly of large-scale network models that can provide important new insights into living systems. However, assembly and validation of such large-scale models can be challenging, since we often lack sufficient information to make accurate predictions. This work describes a new approach for constructing large-scale transcriptional regulatory networks of individual cells. We show that the reconstructed network captures a significantly larger fraction of cellular regulatory processes than networks generated by other existing approaches. We predict this approach, with appropriate refinements, will allow reconstruction of large-scale transcriptional network models for a variety of other organisms. As we work towards modeling the function of cells or complex ecosystems, individually reconstructed network models of signaling, information transfer and metabolism, can be integrated to provide high information predictions and insights not otherwise obtainable.

## Introduction

Coordinating cellular behavior in response to internal or external signals requires dynamic regulation at several levels [1,2]. Our ability to understand cellular dynamics requires detailed knowledge of each regulatory network and will, in part, depend on our ability to reconstruct models that integrate the datasets that report on these processes. Of the various levels at which cellular activities are regulated, transcriptional regulatory networks (TRNs) represent a particularly active area for modeling, as high-throughput techniques to monitor RNA levels and protein-DNA interactions can be applied in a wide range of organisms [2,3]. Using such datasets, one can analyze, model, and reverse-engineer TRNs [3,4].

Many published approaches to TRN inference depend on gene expression datasets to make predictions about direct interactions between transcription factors (TFs) and their target genes, assuming that the expression profile of a gene or cluster of genes, is directly related to that of a cognate TF(s) [5–11]. However, predictions based on this premise alone can be compromised by well-known indirect effects (e.g., co-expressed but not co-regulated genes) and post-transcriptionally regulated TFs, whose cellular levels remain relatively constant under conditions where their activity is significantly altered. In attempts to improve the TRN inference process, sequence analysis of the promoter regions of target genes has been used to inform models on the likelihood of a TF directly regulating a set of target genes [5,6,12–16]. However, there is intrinsic statistical variability in the definition of gene clusters obtained from co-expression analyses. Consequently, identifying directly co-regulated genes (i.e., genes that are both co-expressed and share conserved upstream regulatory sequences) is particularly challenging, as *de novo* identification of functional DNA binding motifs from co-expression clusters is hampered by the fact that the functional sequences of interest are often underrepresented [17].

Comparative genomics analysis of closely related organisms can facilitate identification of functional regulatory motifs by increasing the signal to noise ratio in the input DNA sequences that are used for *de novo* motif detection [13–15]. The apparent conservation of TFs and regulatory interactions across species has been leveraged to build TRNs across related species [13–16]. However, computational prediction of the presence of a shared DNA motif that is associated with the promoter in a group of genes should not be the only criterion for determination of co-regulation, as co-regulated genes would also be expected to share similar expression profiles under some conditions.

While these individual approaches to TRN inference have their strengths and limitations, they can be complementary and could potentially be combined to construct TRNs of greater coverage and better predictive power [3,6]. However, no integrated workflow currently exists that systematically combines these potentially complementary concepts. Thus, we sought to develop an approach for reconstructing large-scale TRNs that would integrate these various ideas to generate TRN models with higher information content and greater depth.

To achieve this goal, we developed a workflow to construct TRNs, which integrates comparative genomics data, global gene expression analyses, and intrinsic properties of transcription factors (TFs). Intrinsic properties comprise several well-known characteristics of bacterial TFs such as the proximity of TF structural genes to their binding sites [12,14,18,19], the correlation of expression profiles of TFs and their target genes [3,6–8], the similarity in DNA motifs bound by TFs having similar DNA binding domains [19,20] and the co-occurrence of TFs and their binding sites across species [19]. While these properties are established features of many bacterial TFs, they have not been systematically leveraged in the large-scale inference of TRN models. We assessed the function of such an integrated workflow using benchmark datasets for the well-studied bacterium *Escherichia coli* and we show that it is able to capture a significant portion of the known *E*. *coli* TRN. Furthermore, we show this integrated network provides significantly improved predictive power over expression-based inference approaches. We also observed that the content of the TRN models derived from our integrated workflow and from expression-based approaches are complementary, providing an opportunity to combine the TRN models derived from these different approaches.

We also used this workflow to construct and evaluate a large-scale TRN model for the metabolically versatile α-Proteobacterium *Rhodobacter sphaeroides*. *R*. *sphaeroides* is a purple non-sulfur bacterium that has been studied for decades as a model system for photosynthetic growth, being used to understand photon capture, light-driven energy metabolism, and other aspects of the photosynthetic lifestyle [21,22]. In addition to anoxygenic photosynthetic growth, this facultative bacterium is capable of aerobic and anaerobic respiration [22]. *R*. *sphaeroides* can also fix CO_2_ and N_2,_ and produce H_2_, polyhydroxybutyrate or other compounds of industrial importance [21–30]. Thus, gaining a detailed understanding of its TRN will be pivotal in extending our knowledge of how these various lifestyles and metabolic processes are regulated. Using our integrated workflow, we identified clusters of co-regulated genes in *R*. *sphaeroides* and made predictions on DNA binding proteins that are likely to regulate these gene clusters. By focusing on several major sub-networks, we show that predictions of our TRN are consistent with prior knowledge in *R*. *sphaeroides* and related bacteria. In addition, experimental analysis of select TFs using chromatin immunoprecipitation followed by high-throughput sequencing (ChIP-seq) and global gene expression analyses provided direct validation of the predictive power of this large-scale *R*. *sphaeroides* TRN model. Our analyses illustrate the utility of this integrated approach to assemble TRN models that provide new insights into important biological processes and highlight the role of large-scale TRN inference in driving scientific discovery.

## Results and Discussion

### TRN Inference

**Fig 1:**
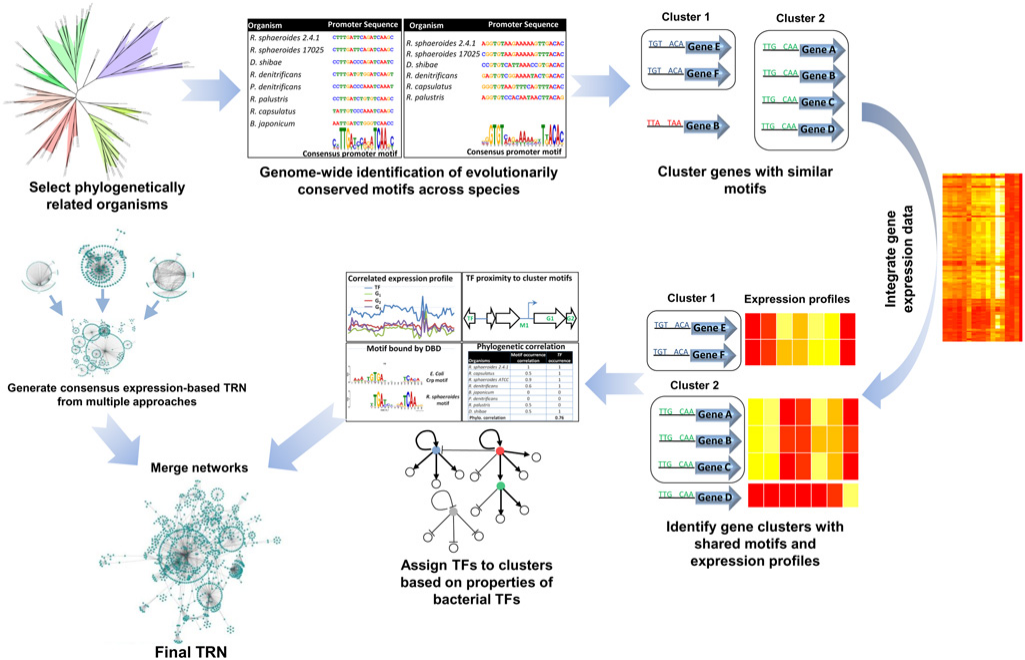
Overview of TRN reconstruction approach. A summary of the various steps involved in our TRN reconstruction workflow.

We developed an integrated inference approach to reconstruct large-scale TRNs that uses both sequence information from closely related bacteria and gene expression data, while taking into consideration known properties of bacterial TFs (summarized in Fig. 1). Gene clusters generated by this integrated approach could conceptually be thought of as being co-regulated, as they would share similar expression profiles and evolutionarily conserved upstream DNA sequence motifs. Furthermore, the prediction of TFs that directly control expression of these co-regulated clusters would not depend solely on expression information, potentially enabling more accurate TF-cluster assignments, even for post-transcriptionally regulated TFs whose expression profiles might be unrelated to those of their target genes.

The key steps in our workflow are summarized in S1 Fig., with implementation details of each step provided in the Material and Methods section. Several of these steps involve the use of a variety of well-established public domain algorithms and software packages, which are systematically integrated with new algorithms to build an automated workflow. Below, we summarize the keys steps in this workflow.

**Selecting organisms for phylogenetic footprinting**. To incorporate comparative genomics into TRN inference, our workflow begins with the selection of appropriate organisms for phylogenetic footprinting. The selection of organisms is critical for this analysis, as organisms that are too closely related may be uninformative, while organisms that are too distantly related may not possess conserved regulatory modules to inform the construction of highly predictive models (see Materials and Methods). Our analysis indicates that as few as 6 appropriately selected organisms could be sufficient to conduct a robust analysis, with addition of more species only providing marginal benefit to the TRN predictions (S2 Fig.). However, as the most appropriate organisms to use are not always known *a priori*, using a larger selection of organisms may be beneficial.

**Identification of orthologs**. Prior to de novo motif detection, orthologous genes shared between the selected organisms have to be identified. Approaches for predicting orthologs such as bidirectional best BLAST hits can provide satisfactory results for ortholog predictions in prokaryotic genomes [31]. However, the orthoMCL algorithm [32], which builds on bidirectional best BLAST hits by implementing additional normalizations for protein lengths and uses the Markov cluster algorithm (MCL) [33] to group orthologous proteins from multiple species, provides an automated approach to ortholog identification across multiple organisms that can yield improved results. Thus, our workflow leverages orthoMCL analysis to identify orthologs shared among the organisms selected for the analysis, with all orthologs of a given gene forming an orthologous group.

***De novo* motif detection**. After identification of orthologs shared across species, *de novo* motif detection analysis is conducted on the intergenic regions of all the genes coding for proteins within a given orthologous group (S2 Fig.). From our analysis, we found that MEME [34] enabled the identification of a wide variety of evolutionarily conserved motifs and performed better than a Gibb’s sampling based approach [35,36]. These evolutionarily conserved DNA motifs are then used to scan the entire genome for other candidate sites, which are clustered based on sequence similarity (see Materials and methods). This results in the generation of clusters of genes with conserved upstream DNA sequence motifs.

**Integration of gene expression data**. In addition to containing shared upstream regulatory motifs, co-regulated genes might also be expected to have common or similar expression patterns, at least under a subset of conditions. Thus, approaches for reconstruction of TRNs should use both types of information, when available, to build higher confidence networks. To integrate information captured in comparative genomics-based gene clusters with gene expression data, our workflow uses DISTILLER [37]. DISTILLER is a bi-clustering algorithm that identifies conditions or sub-conditions (biclusters) under which a group of genes share a strong co-expression pattern, as condition-dependent regulation of genes means they may not share strong co-expression profiles across the entire dataset. Thus given pre-specified a group of genes (in this instance based on the presence of a shared evolutionarily conserved motif), DISTILLER is used to identify sub-conditions under which these genes share a significant co-expression pattern. We use this approach to generate clusters of “co-regulated” genes having both shared DNA sequence motifs and gene expression patterns.

**Linking TFs to clusters**. The task of predicting the TF(s) that regulate genes or gene clusters is typically carried out by assessing the relationship between the expression profiles of TFs and their predicted targets [3,5,9,38]. While this approach has been successfully applied in bacterial systems, it is of limited use in eukaryotes [3,38]. However, even in bacteria many TFs are post-transcriptionally regulated, and therefore, their expression profiles are unlikely to share any relationship to those of their target genes. This can lead to spurious predictions when using gene expression data alone. Use of prior knowledge about the properties of TFs, beyond just correlated expression profiles, could facilitate prediction of target genes of such TFs. Thus, in order to link known or predicted TFs to the putative co-regulated gene clusters, our workflow takes advantage of four known characteristics of bacterial TFs: (i) *correlation* in expression profiles between a TF and its target genes [3,6–8]; (ii) *proximity* of a TF to the location of the closest binding site within a given cluster (since many bacterial TFs are either auto-regulatory or bind to locations in close proximity to their structural genes) [12,14,18,19]; (iii) similarity in DNA motifs bound by TFs having similar *DNA binding domains* (since TFs belonging to the same protein families often bind to similar DNA sequence motifs) [19,20]; and (iv) *phylogenetic correlation* of the occurrence of a TF and occurrence of a DNA sequence motif across species, (assuming that a DNA sequence motif is likely present in an organism if the TF which recognizes this site is also encoded in its genome) [19].

Given the set of known or predicted TFs in the organism of interest, T = {TF_1_, …, TF_i_} (where *i* is the total number of TFs in the organism), and the set of all predicted gene clusters, C = {Cluster_1_, …, Cluster_j_} (where *j* is the total number of predicted clusters), these four properties are integrated as follows:

**Correlation**: To use correlation to discriminate between potential transcriptional regulators of a cluster of putatively co-regulated genes, the average Pearson’s correlation coefficient (Corr_mean_) was determined for each TF per gene cluster (eqn. 1) (S3A Fig.). This was achieved by determining the correlation of the expression values between a given TF (TF_x_) and each gene (g_k_) within a given cluster (Cluster_y_) containing n genes, across the subsets of conditions under which the gene is tightly co-expressed with others in the cluster. The absolute values of these TF-gene correlations are then averaged to obtain a TF-cluster Corr_mean_ (eqn. 1). This is carried out for all TFs in the target organism to determine the average correlation of each TF in relation to each cluster. These average correlation scores are then converted into p-values (**P**_corr_) by random permutation. Briefly, 1000 TF-cluster Corr_mean_ scores were randomly generated, then each previously calculated TF-cluster Corr_mean_ was compared to the set of randomly generated values. The total number of randomly generated scores greater than or equal to a given TF-cluster Corr_mean_ divided by 1000 was used as an estimate of the p-value (eqn. 2).

$$\begin{array}{l} Corr_{mean}\left(TF_{x},\;Cluster_{y}\right)\;=\frac{1}{n}\sum_{k=1}^{n}\;|corr\;(TF_{x},Cluster_{y}(g_{k}))|\\ \;\forall TF_{x}\in T\;and\;\forall Cluster_{y}\in C \end{array}$$ (1)

$$\begin{array}{l} P_{corr}\left(TF_{x},\;Cluster_{y}\right)\;=\frac{\text{Total no. of random scores}\geq {\text{ Corr}}_{\text{mean}}{\text{ (TF}}_{\text{x}}{\text{, Cluster}}_{\text{y}}\text{)}}{\text{1000}}\\ \forall TF_{x}\in T\;and\;\forall Cluster_{y}\in C \end{array}$$ (2)

**Proximity**: To use the proximity of TFs to link them to their binding sites, we determined the minimum distance (in number of genes) between each TF’s location in the genome and the genes present in a given cluster (eqn. 3) (S3B Fig.). Here, the proximity score would have a value of 0 (if the TF is a member of a cluster for a given TF-cluster pair) or larger. This proximity score is determined for every TF-cluster pair where at least one member of the cluster is located on the same replicon as the TF. These minimum distance scores (Prox_min_) were also converted into p-values (**P**_prox_) by random permutation as described above (eqn. 4).

$$\begin{array}{l} Prox_{min}\left(TF_{x},\;Cluster_{y}\right)\;=Min(Dist(TF_{x},Cluster_{y}(g_{1})),\ldots ,Dist(TF_{x},Cluster_{y}(g_{n})))\\ \forall TF_{x}\in T\;and\;\forall Cluster_{y}\in C \end{array}$$ (3)

$$\begin{array}{l} P_{prox}\left(TF_{x},\;Cluster_{y}\right)\;=\frac{\text{Total no. of random min. dist.}\leq {\text{ Prox}}_{\text{min}}{\text{ (TF}}_{\text{x}}{\text{, Cluster}}_{\text{y}}\text{)}}{\text{1000}}\\ \forall TF_{x}\in T\;and\;\forall Cluster_{y}\in C \end{array}$$ (4)

**DNA binding domain**: To incorporate information on DNA binding domain (DBD) similarity into TRN predictions, we begin by determining the DBD family to which each TF in the target organism belongs to using Pfam analysis [39]. All *E*. *coli* TFs from RegulonDB [40], which had binding motif information (81 at the time of this analysis), were retrieved and their DBD families also determined using Pfam. Position specific scoring matrices (PSSMs) for the DNA binding sites of the RegulonDB TFs and each of the evolutionarily conserved *de novo* detected motifs are then constructed. For each TF-cluster pair to be assessed, the PSSM for the *de novo* detected motif of the cluster under consideration was compared to the PSSM(s) from *E*. *coli* whose associated TF(s) belongs to the same DBD family as the TF under consideration. This TF was then assigned the most significant (smallest) q-value from this set of comparisons. For instance, if TF_x_ is a Crp family TF, to assign a score to TF_x_ in relation to Cluster_y_, the PSSM for Cluster_y_ is compared to all available Crp family PSSMs from the RegulonDB data set and TF_x_ is assigned a value equivalent to the most significant match to these PSSMs (S3C Fig.). These q-values were then—log_10_ transformed to generate the DBD_score for that TF-cluster pair. PSSM comparisons were made using Tomtom [41,42] and all possible TF-cluster pairs were assessed similarly. These DBD_scores were converted into p-values (**P**_dbd_) by random permutation as previously described (eqn. 5).

$$\begin{array}{l} P_{dbd}\left(TF_{x},\;Cluster_{y}\right)\;=\frac{\text{Total no. of random DBD scores}\geq {\text{ DBD\_score (TF}}_{\text{x}}{\text{, Cluster}}_{\text{y}}\text{)}}{\text{1000}}\\ \forall TF_{x}\in T\;and\;\forall Cluster_{y}\in C \end{array}$$ (5)

**Phylogenetic correlation**: To compute a score for this property, we first determine the occurrence of a given motif across all the genomes used in the analysis. For each *de novo* detected motif, we use MAST [41] to search for all instances of that motif in the intergenic regions of each organism used for phylogenetic footprinting. These genome-wide p-values of MAST hits for a given motif were stored in separate vectors for each genome. The correlation was then calculated between the MAST hits p-value vector of the target organism and that for each species used for phylogenetic footprinting (target organism inclusive). These correlations were referred to as “motif occurrence correlations” (S3D Fig.). We then determined the occurrence of each TF in the target organism across all the species used for phylogenetic footprinting via orthoMCL analysis. Finally, the correlation between the “motif occurrence correlation” and TF occurrence was calculated to determine the phylogenetic correlation. These phylogenetic correlation scores were converted to p-values by random permutation as described above (eqn. 6).

$$\begin{array}{l} P_{pc}(TF_{x},Cluster_{y})=\frac{\text{Total no. of random Phylo. corr. scores}\geq \text{ Phylo. corr}{\text{. score (TF}}_{\text{x}}{\text{, Cluster}}_{\text{y}}\text{)}}{\text{1000}}\\ \forall TF_{x}\in T\;and\;\forall Cluster_{y}\in C \end{array}$$ (6)

**Combining scores**: To rank candidate TFs, the -log_10_ of the computed p-values for the 4 different criteria were summed together to generate a final score **R**_score_ (eqn. 7), resulting in a ranked list of TFs most likely to regulate a given cluster.

$$\begin{array}{l} R_{score}\left(TF_{x},\;Cluster_{y}\right)\;=–log_{10}(P_{corr}\left(TF_{x},\;Cluster_{y}\right)*P_{prox}\left(TF_{x},\;Cluster_{y}\right)*P_{dbd}\left(TF_{x},\;Cluster_{y}\right)*P_{pc}\left(TF_{x},\;Cluster_{y}\right))\\ \forall TF_{x}\in T\;and\;\forall Cluster_{y}\in C \end{array}$$ (7)

**Predicting regulatory interactions from global gene expression data**. The integrative approach described above identifies conserved clusters of putatively co-regulated genes, but its utility can be limited by the evolutionary distance and the degree of conservation of the individual regulatory modules across the organisms used to generate the TRN. For example, it may be difficult to identify conserved regulatory sequences across closely related species if these sequences or regulatory mechanisms have undergone significant evolution. Furthermore, individual sub-networks that are specific to a lifestyle or response of an individual species, genus and/or clade might not be captured via a comparative genomics-based approach. Thus, to complement predictions from the comparative genomics-based analysis, we considered the consensus predictions of multiple high performing direct expression-based inference approaches [3,9–11] to make predictions for additional TFs not included in the comparative genomics-based TRN. In particular, we used the consensus predictions from 3 approaches: context likelihood of relatedness (CLR), which uses normalized mutual information-based scores, as an indication of the relatedness of expression profiles, to assess potential TF-target interactions [9]; GENIE3, which uses multiple regression and tree-based feature selection to identify TFs whose expression profiles are most predictive of a given target gene [10]; and an approach which uses analysis of variance (ANOVA) to score how dependent the expression profile of a target gene is to potential transcriptional regulators [11]. The predictions from these approaches were combined using methods similar to those previously used for generating consensus networks from approaches assessed in the DREAM challenges [3]. Details of this are provided in the Materials and methods (see “Inferring regulatory interactions solely from expression data”).

The networks predicted from comparative genomic-based integration and the gene expression-based consensus network were then combined. This was achieved by taking the integrated comparative genomic-based TRN as the core of the network, then augmenting it by including high-scoring predictions for TFs not already included in the integrated network. We chose this approach based on observations from analysis of the *E*. *coli* TRN (see below).

### An integrated approach improves overall predictive performance

To assess the performance of the integrated workflow outlined above, we built a TRN for *E*. *coli* using sequence data from 14 enterobacteriales species (including *E*. *coli*) obtained from NCBI and curated expression data obtained from the many microbes microarray database [9]. The TRN built using the described integrative comparative genomics-based component of our workflow consisted of 225 motifs and clusters, 1660 genes, 126 TFs and a total of 2457 interactions (S1 Dataset). In addition, 156 of the 225 clusters were significantly enriched for at least one functional category. These predictions were compared to similar sized TRNs (2500 highest ranked interactions) generated by CLR and GENIE3 using the same gene expression dataset. These TRN models were then validated against an experimentally verified list of regulatory interactions from regulonDB [40].

**Fig 2:**
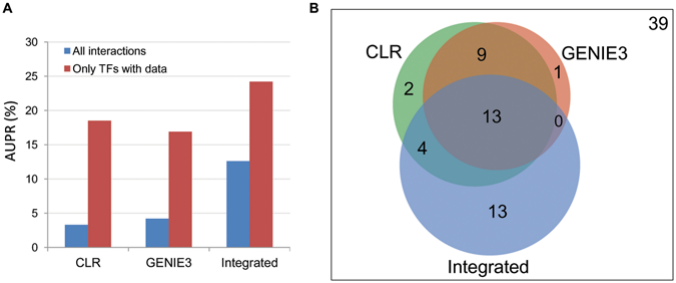
Comparison of the performance of CLR, GENIE3 and integrated approaches on *E*. *coli* dataset. (A) AUPR for CLR, GENIE3 and the integrated approach when predictions for all 267 input candidate TFs are considered (blue bars) or when only interactions for the 81 experimentally verified TFs in RegulonDB are considered (red bars). (B) Venn diagram summarizing the overlap between the 3 approaches for the TFs for which at least one accurate prediction was made.

A widely used metric for assessing the performance of TRN inference approaches is the area under a plot of precision against recall for inferred TF-target gene interactions [4,43]. Assessing this area under the precision-recall curve (AUPR), we observed that the integrated approach performed significantly better than CLR or GENIE3, both when all predicted interactions were considered (AUPR ~3 times larger) and when only interactions for TFs with experimental data were considered (AUPR ~1.5 times larger) (Fig. 2A). At a precision of 25%, CLR and GENIE3 and the integrated TRNs achieved a recall of 1.8%, 2.1% and 5.7%, respectively. These analyses indicate that our comparative genomics-based integrated approach is more accurate and able to capture a larger fraction of known regulatory interactions. It should be noted that for this analysis, only the highest scoring TF predicted for each cluster was used to build the final list of predicted interactions for the integrated TRN. In some instances other high scoring TFs may actually be the direct regulators, but these were not considered here.

**Integrated and expression-based networks are complementary**. While the above analysis highlights the improved performance of the integrated approach over the expression-only TRN inference, it may be more informative to examine the predicted interactions and assess where each approach excels or fails, to determine if there is any complementarity between these approaches. Of the 81 TFs for which experimentally verified interactions exist in the regulonDB dataset used in our analysis, CLR, GENIE3 and the integrated TRN models were able to make at least one correct prediction for 28, 23 and 30 of these TFs respectively (Table 1, Fig. 2B). While CLR and GENIE3 use different approaches to infer their TRNs, there is a large overlap in the TFs for which they make predictions (Fig. 2B, Table 1). This is consistent with previous observations from analysis of TF-target interactions conducted as part of a comprehensive assessment of expression-based inference approaches [3]. Overall 96% of the TFs for which GENIE3 made correct predictions could also be captured using CLR and 79% vice versa, though the precision and recall for each of these TFs varies between approaches (Table 1). Conversely, only 57% and 61% of the TFs for which CLR and GENIE3 made predictions for, respectively, also had predictions in the integrated TRN, while predictions for 43% of the TFs in integrated network were unique to this approach (Fig. 2B).

**Table 1:** Predictions for *E*. *coli* TFs.

			Integrated*		CLR		GENIE3	
	TFs†	Regulon size	Prec. (%)	Rec. (%)	Prec. (%)	Rec. (%)	Prec. (%)	Rec. (%)
**A**	**b1013 (RutR)**	17	100	52.9	0	0	NA	NA
	**b3438 (GntR)**	12	100	41.7	0	0	0	0
	**b4178 (NsrR)**	83	100	3.6	NA	NA	NA	NA
	**b0683 (Fur)**	129	68.6	27	0	0	0	0
	**b1712 (IhfA)**	219	66.7	1.8	NA	NA	NA	NA
	**b0080 (Cra)**	78	52	15	NA	NA	0	0
	**b3512 (GadE)**	36	50	8.3	NA	NA	NA	NA
	**b1334 (FNR)**	296	39	3	0	0	0	0
	**b3938 (MetJ)**	15	30.8	26.6	0	0	NA	NA
	**b3094 (ExuR)**	8	21	37.5	0	0	0	0
	**b3237 (ArgR)**	37	21	35	NA	NA	NA	NA
	**b2369 (EvgA)**	18	5.5	5.5	0	0	0	0
	**b1508 (HipB)**	2	0.8	100	0	0	NA	NA
**B**	**b3418 (MalT)**	10	88.9	80	66.6	40	NA	NA
	**b3828 (MetR)**	5	66.7	40	100	20	NA	NA
	**b1221 (NarL)**	121	25	0.8	25	0.8	0	0
	**b0076 (LeuO)**	20	15.5	10	8.3	5	0	0
**C**	**b3569 (XylR)**	6	80	66.6	20	16.7	100	16.7
	**b3868 (GlnG)**	44	86.4	43.2	8.3	2.3	100	2.3
	**b0064 (AraC)**	11	75	27	54.5	54.5	88.8	72
	**b0399 (PhoB)**	60	53.3	26.7	8.3	1.7	40	3.3
	**b4043 (LexA)**	59	59.57	47.5	76	32.2	61.8	35.6
	**b3357 (Crp)**	497	39.4	13.5	50	0.2	22.2	0.4
	**b1658 (PurR)**	31	26.1	58.1	23.5	12.9	22	35.5
	**b0889 (Lrp)**	105	25	1	14.2	1.9	12.9	13.3
	**b1014 (PutA)**	2	13.3	100	20	50	33.3	50
	**b0113 (PdhR)**	42	10.7	7.1	71.4	11.9	100	14
	**b0020 (NhaR)**	7	10.5	28.5	50	14.3	100	14.3
	**b3702 (DnaA)**	12	6.8	33.3	10	16.7	3.5	16.7
	**b3912 (CpxR)**	63	6.6	1.6	10.5	3.2	14.3	3.2
**D**	**b2151 (GalS)**	10	0	0	33.3	30	100	30
	**b2731 (FhlA)**	30	NA	NA	9	3.3	100	3.3
	**b1531 (MarA)**	38	NA	NA	40	5.3	50	5.3
	**b2531 (IscR)**	32	NA	NA	38.5	15.6	31.3	15.6
	**b3905 (RhaS)**	6	NA	NA	9.8	66.6	26.6	66.7
	**b0676 (NagC)**	36	NA	NA	20	2.8	25	2.8
	**b3021 (MqsA)**	4	NA	NA	11.1	25	20	25
	**b1040 (CsgD)**	23	0	0	33	13	12.1	17.4
	**b3261 (Fis)**	227	0	0	10.5	0.9	6.7	4
**E**	**b1130 (PhoP)**	55	NA	NA	20	1.8	NA	NA
	**b4324 (UxuR)**	7	NA	NA	16.66	12.5	NA	NA
**F**	**b1988 (Nac)**	21	NA	NA	0	0	2.1	4.7
	**Average**		40.7	28.6	23.2	12.5	34.6	14.6

* NA—Not applicable i.e., no predictions made by inference approach for that TF. A value of 0 indicates some predictions were made but all were inaccurate. Prec.—precision; Rec.—recall.

† TFs for which accurate predictions were made by: A—only integrated approach; B—both CLR and the integrated approach; C—all 3 inference approaches; D—only CLR and GENIE3; E—only CLR; F—only GENIE3.

These observations indicate that there are specific subsets of TFs that are amenable to predictions using expression-based assumptions. However, many TFs that are not amenable to analysis solely by expression-based analyses can be correctly assigned in a TRN constructed using an integrative approach. This is potentially due to instances where the expression profile of a TF does not show any significant relationship to those of its target genes. This typically occurs for TFs that are known to be post-translationally regulated such as FNR, ArgR, Fur, Cra etc [44–47] (Table 1). On the other hand, for several of the TFs where expression-based approaches performed better, the integrated approach failed to make any prediction (Table 1). This could be the result of a number of factors including lack of conservation of TF binding sites, small regulon size, complex DNA binding motifs or limitations in the motif detection algorithm utilized. Importantly, for TFs for which predictions were made by all three approaches, the predictions from the integrated approach were in general on par with, or better than, those obtained with expression-based approaches (Table 1). These observations lend themselves to a straight-forward approach for combining these approaches wherein the integrated comparative genomics-based network serves as the core of the TRN, and is complemented with high scoring predictions from expression-based approaches for TFs not already captured in the core network.

### Overview of the Inferred TRN for *R. sphaeroides*

Using the same workflow and leveraging the observed complementarity of integrative and expression-based approaches, we generated a large-scale TRN model for the metabolically versatile photosynthetic bacterium *R*. *sphaeroides*. In this case, we used sequence information from 8 closely related α-Proteobacteria, including *R*. *sphaeroides* (S4 Fig.) and gene expression data from 198 experiments (S1 Dataset). The resulting TRN model consists of 120 clusters, 93 TFs, 76 distinct evolutionarily conserved DNA sequence motifs and 1858 TF (or motif)-target interactions (S5 Fig., S1 Table). This model includes a total 1211 *R*. *sphaeroides* genes (about 28% of the open reading frames predicted in its genome [48,49]). Below, we provide an overview of some of the pertinent predicted sub-networks in the TRN, as well as experimental validation of some key TFs in the network.

**Fig 3:**
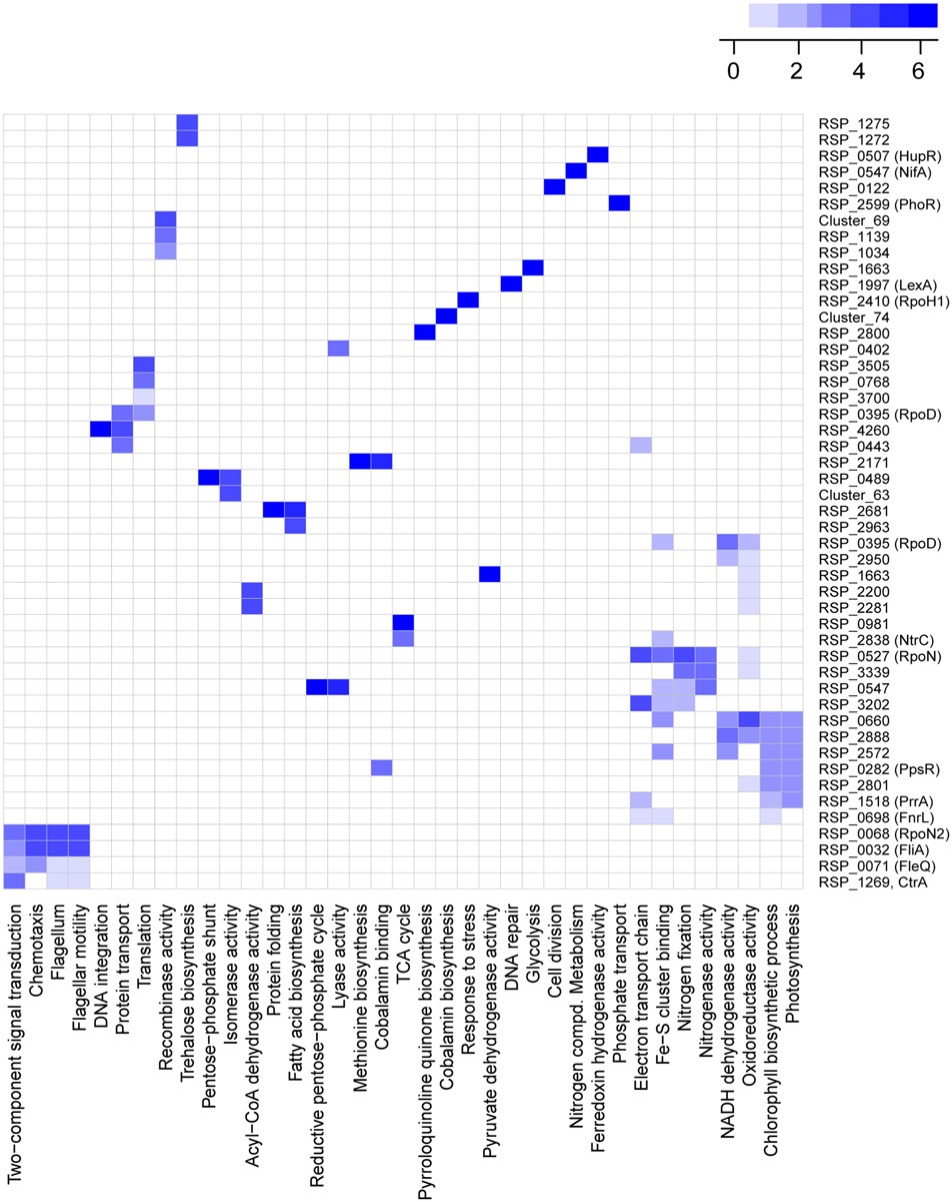
Overview of functional categories captured in the *R*. *sphaeroides* TRN. Heat map showing the most significantly enriched GO terms for 48 of the 120 clusters identified in our analysis. The predicted regulators for each cluster is shown on the right hand side of the map, while the GO categories are at the bottom. Darker shades of blue indicated greater significance.

**Reconstructed TRN encompasses a wide variety of functions**. The *R*. *sphaeroides* TRN model encompasses a wide variety of cellular functions ranging from central carbon metabolism and global stress responses, to processes more specific to *R*. *sphaeroides*, such as nitrogen fixation and photosynthesis (Fig. 3, S5 Fig.). Of the 120 identified gene clusters, 80 were significantly enriched for at least one gene ontology (GO) [50] category (S1 Table, Fig. 3), indicating this TRN model captures a high degree of functional information even though this type of functional data was not used in the network inference workflow.

**Fig 4:**
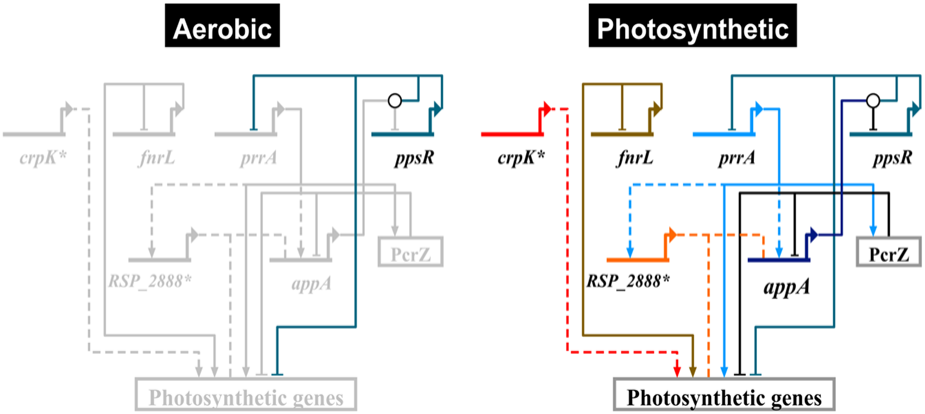
Photosynthetic gene regulatory network. An overview of the *R*. *sphaeroides* photosynthetic gene regulatory network, showing all the known/predicted transcriptional regulators. Solid lines indicate experimentally verified interactions, while dashed lines indicate predicted but as yet unverified interactions. Under aerobic conditions, AppA—the anti-repressor of PpsR—is inactive, allowing PpsR to repress photosynthetic genes (grey nodes and edges indicate inactivity). Under photosynthetic conditions, AppA becomes active and interacts with PpsR via protein-protein interactions (depicted with a white circle), thereby inhibiting PpsR repression. The transcriptional activators, PrrA and FnrL become active under these conditions and drive the expression of photosynthetic genes. CrpK and RSP_2888 (MppG) are also predicted to be involved in this process. PcrZ is a sRNA shown to negatively impact photopigment gene expression under photosynthetic conditions. Biotapestry was used for network visualization [62]. * indicates newly added components of the photosynthetic gene regulatory network identified in this study.

**Photosynthesis**. Previous analyses of the photosynthetic lifestyle of *R*. *sphaeroides* have implicated 3 TFs in this process: PpsR [51,52], FnrL (a homolog of FNR) [53–55] and PrrA (the response regulator of the PrrAB two component system) [56–60] (Fig. 4). More recently a small non-coding RNA, PcrZ has been implicated in the regulation of photosynthesis in *R*. *sphaeroides* [61]. Despite extensive prior analysis, our TRN model predicts at least 2 additional regulators of photosynthesis: CrpK (RSP_2572) and RSP_2888 (Fig. 4). To illustrate the predictive ability of our TRN, below we provide details about the known or predicted TFs in the *R*. *sphaeroides* photosynthetic lifestyle.

**Fig 5:**
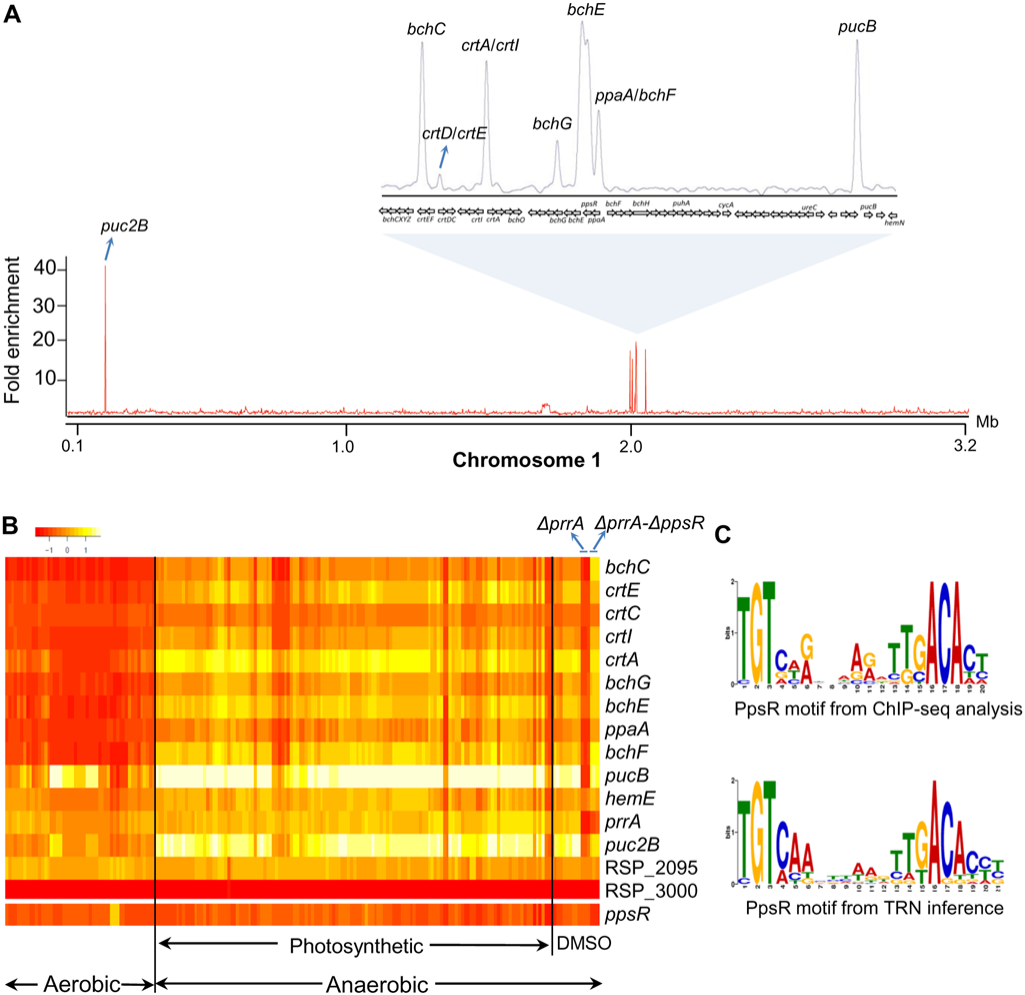
Analysis of the PpsR regulon in *R*. *sphaeroides*. (A) Using ChIP-seq, we identified the binding sites for PpsR across the *R*. *sphaeroides* genome, with several binding sites across chromosome 1 highlighted. MochiView [69] was used for visualization of binding profile. (B) Heat map depicts the expression profiles of the first members of PpsR targets operons across our microarray compendium of 198 experiments conducted under aerobic respiratory (Aerobic), anoxygenic photosynthetic (Photosynthesis) and anaerobic respiratory conditions (DMSO). Expression profiles for experiments conducted on the *ΔprrA* and *ΔprrA-ΔppsR* strains are highlighted. Deletion of PpsR from *ΔprrA* results in derepession of PpsR target genes. (C) Position weight matrix logo generated for PpsR using targets identified by ChIP-seq compared to logo generated from our TRN inference analysis.

Previous analysis of **PpsR (RSP_0282)** identified this TF as a repressor of photopigment production under aerobic conditions [51,52,63,64]. The activity of PpsR is regulated by its cognate anti-repressor, AppA, which is reported to respond to both oxygen and blue light [65–68]. To gain a more complete picture of the PpsR regulon, as well as assess the predictive performance of our inferred TRN for this TF, we determined the genome-wide binding of PpsR to its target sites by ChIP-seq using a 3X-myc tagged PpsR protein that complements a defined *ΔppsR* mutant. We identified a total of 19 PpsR binding sites in the genome that were located upstream of 15 operons, only 2 of which had been previously verified as direct targets for this TF [51] (Table 2, Fig. 5A). Consistent with its role in regulation of photopigment formation, the majority of PpsR target operons had known or predicted photosynthesis-related functions (Table 2). Interestingly, PpsR was bound upstream of the *prrA* gene, which encodes another transcriptional regulator of photosynthesis in *R*. *sphaeroides* [56–60], suggesting a previously unknown genetic interaction between these TFs.

**Table 2:** PpsR binding sites across the *R*. *sphaeroides* genome identifed by ChIP-seq.

	ID	Annotation	chrID	peakStart	peakStop	FC^a^	Motif	Expr^b^
1	RSP_0263–59^c^	*bchCXYZ-pufQ*	chr1	1987800	1988799	24.3	**TGT**CCAATAAAGTTG**ACA**CT	-36.61
2	RSP_0265–4^c^	*crtEF*	chr1	1990400	1990799	3.3	**TGT**AAGAAAAAGTTG**ACA**CC	-8.98
	RSP_0266–7^c^	*crtCD*						-2.65
3	RSP_0271–69^c^	*crtIB-tspO*	chr1	1996200	1996799	20.2	**TGT**CTAGTCAGGTTT**ACA**AT	-11.75
	RSP_0272–5^c^	*crtA-bchIDO*						-20.05
4	RSP_0279–6^c^	*bchG-pucC-bchP*	chr1	2005000	2005599	7.8	**TGT**AAGGATAGATTG**ACA**CT	-8.03
5	RSP_0281–80^c^	*bchEJ*	chr1	2007600	2009599	21.9	**TGT**CAACTGAAATGG**ACA**CA	-9.60
6							**TGT**CCAGTGCGTCTG**ACA**CT	
7	RSP_0283* ^,^ ^c^	*ppaA*	chr1	2010000	2010799	12.6	**TGT**CAAAGAAAATTG**ACA**CC	-7.48
8	RSP_0284–91* ^,^ ^c^	*bchFNBHLM-puhA*	chr1				**TGT**AAGTCAGAATTG**ACA**CT	-36.33
9	RSP_0314-RSP_6256^c^	*pucBA*	chr1	2042200	2042799	23.5	**TGT**CAGCGCAATGTG**ACA**CC	-112.17
10							**TGT**CAGCCAACACTG**ACA**TT	
11	RSP_0680	*hemE*	chr1	2424000	2424400	1.7	**TGT**CCATTTGCCCTG**ACA**AC	-2.23
12	RSP_1518^c^	*prrA*	chr1	105181	105204	2.1	**CGT**CAAAGGAAGTTG**ACA**CA	NA
13	RSP_1556-RSP_6158^c^	*puc2B2A*	chr1	146000	146599	58.4	**TGT**CTGCATGGCATG**ACA**TA	-8.99
14	RSP_2095	hypothetical protein	chr1	694600	694999	2.5	**TGT**GTGCGCAGTTGG**ACA**CC	-1.09
15	RSP_3000	hypothetical protein	chr1	1697500	1697700	3	**TGT**CCATATGGGTTG**ACA**TT	-1.21
16			chr1	4000	4200	3.5	**TGT**GTGTCAAGATGC**ACA**CT	ND
17			chr1	1680000	1680599	3.2	**TGT**CTATGACATTTC**ACA**AT	ND
18			chr2	4000	4200	3.4	**TGT**GTGTCAAGATGC**ACA**CT	ND
19			chr2	33000	39599	5	**TGT**GTGTCAAGATGC**ACA**CT	ND

* Previously experimentally verified as direct PpsR target

^a^ Fold enrichment of PpsR-myc ChIP over control myc antibody ChIP in WT.

^b^ Fold change in gene expression from microarray analysis of ΔPpsR and its parental strain. NA—Not applicable (*prrA* is deleted from both strains used for expression analysis). ND—Not determined (binding sites not located upstream of any annotated gene(s)).

^c^ PpsR targets predicted in the TRN

In addition to photosynthesis-related targets, PpsR was bound upstream of RSP_2095 and RSP_3000, which encode proteins of unknown function. However, these genes were not found to be significantly differentially expressed (DE) in a pair-wise comparison of RNA levels between a *ΔppsR* mutant and its parental strain [51], nor did their expression profiles show significant correlation to other members of the PpsR regulon across the available microarray dataset compendium (Fig. 5B), suggesting these might represent non-functional binding sites in the genome, despite possessing strong PpsR motifs (Table 2). Consistent with the known role of PpsR as a transcriptional repressor, all DE PpsR targets we identified were predicted to be repressed by PpsR as RNA levels were increased in cells lacking this TF (Table 2).

Our TRN predicted a total of 13 PpsR target operons, 12 of which were verified via ChIP-seq analysis (S1 Table (cluster 60), Table 2), corresponding to a recall of 80% (i.e., 12 of 15 PpsR ChIP-seq identified sites were predicted) and a precision of 92.3% (i.e., 12 of 13 predicted target sites were accurate). The only predicted PpsR target site not verified by ChIP-seq analysis (RSP_4172—a hypothetical protein) was classified as a false-positive since enrichment for PpsR binding was not detected by subsequent ChIP-qPCR analysis under the growth conditions tested (S6 Fig.). On the other hand, 3 PpsR sites identified in our ChIP-seq assay were not predicted in our TRN (RSP_2095, RSP_3000 and *hemE*). However, given that putative targets such as RSP_2095 and RSP_3000 were not DE in the absence of PpsR (Table 2, Fig. 5B), these might represent non-functional or false positive binding events. Independent ChIP-qPCR validation of ChIP-seq identified sites suggest that RSP_2095 and RSP_3000 are likely bound by PpsR but not DE under the conditions tested (S6 Fig.). Overall, our inferred TRN provided an accurate and expanded picture of PpsR binding sites across the genome with a large coverage of true binding sites. Accordingly, the consensus DNA sequence motifs obtained for PpsR from ChIP-seq and phylogenetic footprinting analysis are very similar (Fig. 5C).

**FnrL (RSP_0698)** is an iron-sulfur cluster-containing Crp-family TF which previous studies have reported to be essential for anaerobic growth in *R*. *sphaeroides* [54,55]. Previous ChIP-chip analysis of genome-wide FnrL binding sites *in vivo* indicated the direct involvement of this TF in a host of processes including photosynthetic and anaerobic respiratory growth [53]. Our inferred TRN captured a significant portion of the known FnrL regulon, predicting a total of 59 FnrL target operons (S2 Table, S1 Table (cluster 11)) that included 24 of the 25 previously identified FnrL target operons, a recall of 96%. The only previously verified FnrL target operon not identified in our analysis was RSP_6116, which is not represented on the *R*. *sphaeroides* Affymetrix gene chip, and thus dropped out during the integration of gene expression data. In addition to previously identified sites, our large-scale TRN predicted an additional 35 FnrL target operons not previously known or predicted to be under the control of FnrL (S2 Table). Each of these new FnrL target operons have putative binding sites with strong similarity to the FnrL consensus and share a similar expression profile with other members of the FnrL cluster (S3 Table). Several of these newly predicted FnrL targets encode functions for which this TF has been previously implicated; including the regulation of Fe-S cluster biogenesis (e.g., RSP_1949) and Fe-S binding proteins (e.g., RSP_0692_89—*rdxBHIS*). However, several new functions for FnrL that are predicted in this data set need to be tested experimentally. If these predictions are correct, it would significantly broaden the functional role of FnrL in this species.

In addition to PpsR and FnrL, whose regulons were globally characterized in this or previous studies, our TRN model also made predictions for direct targets of less-well characterized TFs. For instance, our TRN model made several new predictions for targets of the photosynthesis regulator **PrrA (RSP_1518)**. PrrA has previously been proposed to be major global regulator in *R*. *sphaeroides* and other bacteria [57]. PrrA is essential for photosynthetic growth in *R*. *sphaeroides* and direct control of photosynthesis related operons, tetrapyrolle biosynthesis (*hemA*) and the Calvin—Benson—Bassham (CBB) cycle genes has be shown *in vitro* [59,70]. Our TRN predicts that a total of 17 operons are directly regulated by PrrA (S1 Table (cluster 96)). Of these, 7 predicted PrrA target operons have a photosynthesis related role, including *pufLMX* (RSP_0255–7), *pufA* (RSP_0258), *ppaA* (RSP_0283), *bchFNBHLM-puhA* (RSP_0284–91), *hemC* (RSP_0679), *hemA* (RSP_2984) and *appA* (RSP_1565). However, only two of these operons (*bchF* and *hemA*) have previously been experimentally verified as PrrA-dependent in *R*. *sphaeroides* [70], so direct analysis of PrrA binding to these newly proposed targets is required.

**CrpK (RSP_2572)** is a Crp/Fnr-family TF, which possesses predicted cyclic nucleotide-binding and Crp-like helix-turn-helix domains. However, unlike FnrL, CrpK does not possess N-terminal cysteine residues required for coordination of iron-sulfur clusters, suggesting CrpK might not directly sense oxygen. Our TRN predicts that CrpK regulates overlapping targets to FnrL, including several photosynthesis related operons such as *bchEJGP* (RSP_0281–76) and *hemA* (RSP_2984) (S1 Table (cluster 105)), as well as several other known FnrL target genes including *nuoA-N* (RSP_0100–12) and *ccoNOQP* (RSP_0696–3), amongst others. These predictions suggest CrpK could substitute for FnrL under some conditions, providing added, previously unappreciated, robustness to the photosynthetic TRN of this bacterium and possibly others containing homologs of both FnrL and CrpK. The overlapping nature of the CrpK and FnrL regulons was recently demonstrated experimentally [71].

**RSP_2888** (recently renamed MppG [71]) is a BadM/Rrf2 family TF predicted by our TRN to control photosynthesis gene expression in *R*. *sphaeroides*. Predictions from our TRN suggest a direct role of MppG in the regulation of a bacteriochlorophyll biosynthesis operon *bchFNBHLM* (RSP_0284–91), in addition to key photosynthesis related genes, such as *appA* (RSP_1565) (S1 Table (cluster 110)). MppG mRNA levels are increased under photosynthetic conditions in our expression datasets and this gene is predicted in our TRN to be under the control of PrrA. These observations are consistent with a role for MppG in the photosynthesis sub-network of the TRN, which has been experimentally verified [71].

Overall our TRN captures a significant portion of the known regulatory interactions in the photosynthesis sub-network (Fig. 4), while making a large number of novel predictions that should provide new insights into the complex combinatorial regulation of this lifestyle in PNB.

**Central and alternative carbon metabolism**. For cells to survive in nature, they must adapt to the types and quantities of nutrients present in their environment. For instance, *E*. *coli* uses the cAMP receptor protein (CRP), in part, to preferentially utilize glucose over other nutrient sources, if present in its environment [72]. On the other hand, the ArcAB two-component global regulator represses portions of *E*. *coli*’s central metabolic pathways under anaerobic respiratory conditions [73,74]. In addition to these global regulators, the Cra/FruR regulator specifically regulates carbon and energy metabolism in enteric bacteria [47].

**Fig 6:**
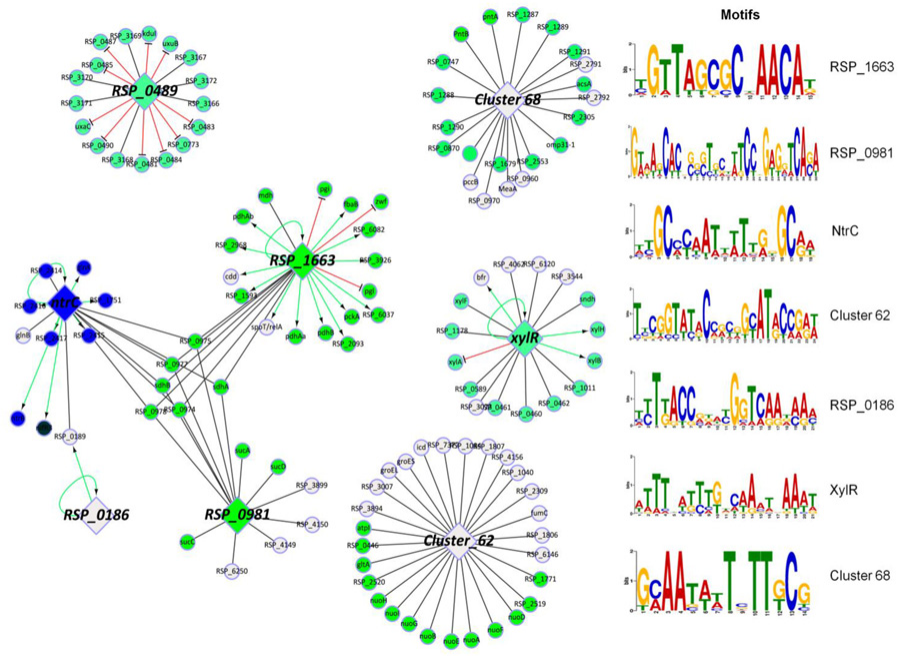
Predicted gene regulatory network controlling central and alternative carbon metabolism in *R*. *sphaeroides*. Sub-network highlighting the regulons of the major TFs predicted to be involved in the regulation of carbon metabolism in *R*. *sphaeroides*. TFs are represented by diamond shaped nodes while non-TF target genes are presented as circular nodes. Green edges represent activation; red edges represent repression, while black edges represent undetermined regulation. Green nodes indicate genes known or predicted to be involved in carbon metabolism, while blue nodes are related to nitrogen metabolism. Motifs predicted to be bound by the various TFs in this sub-network are shown on the right.

*R*. *sphaeroides* is not predicted to possess proteins analogous to CRP or ArcAB. However, our TRN predicts that the regulation of central carbon metabolism in *R*. *sphaeroides* is controlled by a LacI family transcriptional regulator, **RSP_1663**. RSP_1663 is predicted to regulate transcription of genes encoding the central carbon metabolism enzymes Mdh (RSP_0968), PckA (RSP_1680), malic enzyme (RSP_1593), PdhAB (RSP_2968-RSP_4047-RSP_4050), succinate dehydrogenase (RSP_0974–6), as well as glycolytic enzymes Zwf (RSP_2734), Pgl (RSP_2735), Pgi (RSP_2736) and FbaB (RSP_4045), potentially making this TF a major regulator of carbon metabolism under many conditions (Fig. 6). This predicted RSP_1663 regulon might make it functionally analogous to the Cra/FruR regulator in enteric bacteria [47] and the RpiR family TF HexR in β- and γ-proteobacteria [75]. RSP_1663 is predicted to bind to an inverted repeat DNA motif with the sequence [A/G/T]GTT N_6–8_ AAC[A/C/T] (where N is any nucleotide) (Fig. 6). In addition, differences in spacer between the inverted repeats divides the genes predicted to be regulated by this TF into 2 clusters (S1 Table (clusters 15 and 36)). Further experimental analysis is needed to understand the functional role of RSP_1663.

In addition to RSP_1663, **RSP_0981**—a GntR family transcriptional regulator, is predicted to regulate transcription of genes encoding the succinyl-CoA synthetase (RSP_0967–6), succinate dehydrogenase (RSP_0974–6) and α-ketoglutarate dehydrogenase (RSP_0965–62) complexes of the tricarboxylic acid cycle (Fig. 6, S1 Table (cluster 48)), while **NtrC (RSP_2838)** is also predicted to be involved in the regulation of the succinate dehydrogenase complex (Fig. 6, S1 Table (cluster 1)). **Cluster 62** in our TRN (Fig. 6, S1 Table) also contains a number of genes encoding enzymes involved in central carbon metabolism including Icd (RSP_0446 and RSP_1559), L-malyl-CoA lyase (RSP_1771), citrate synthase (RSP_1994) and NuoA-N (RSP_2512–23). The members of cluster 62 share the inverted repeat motif (Fig. 6), indicating that these central metabolism genes are under the joint control of an as yet unidentified TF.

**Fig 7:**
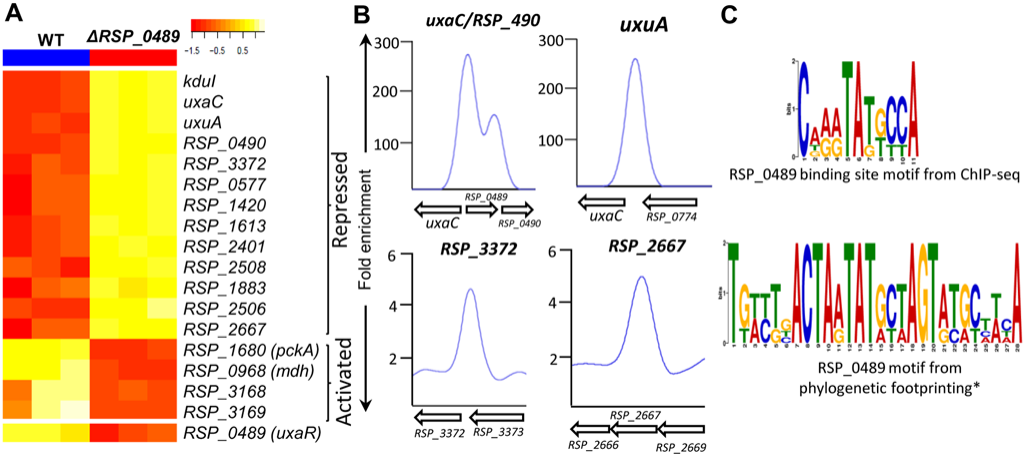
The RSP_0489 regulon. (A) Heat map of metabolic genes DE between wild-type (WT) and *ΔRSP_0489* mutant cells from global gene expression analysis. Only the first members of DE operons are depicted in the heat map for brevity. RSP_0490 (carbohydrate kinase), RSP_3372 (TRAP-T family transporter), RSP_0577 (hypothetical protein), RSP_1420 (TRAP-T family transporter), RSP_1613 (TRAP-T family transporter), RSP_2401 (putative 6-aminohexanoate-cyclic-dimer hydrolase), RSP_2508 (Methylcrotonyl-CoA carboxylase beta chain), RSP_1883 (ABC polyamine/opine transporter), RSP_2506 (Isovaleryl-CoA dehydrogenase), RSP_3168 (ABC transporter), RSP_3169 (FAA-hydrolase-family protein). (B) Direct binding of RSP_0489 to the *uxaC*, RSP_0490, *uxuA*, RSP_3372 and RSP_2667 promoters identified by ChIP-seq. (C) RSP_0489 binding site motif obtained from ChIP-seq analysis compared to that obtained from phylogenetic footprinting analysis of the RSP_0489 promoter.

Our TRN also made predictions about regulation of metabolism of several other carbon sources. For instance, **RSP_0489**—a GntR family transcriptional regulator, is predicted to regulate transcription of genes encoding enzymes that are involved in the metabolism of carboxylic acids including UxuA (RSP_0773), UxaC (RSP_0488), KduID-UxuB (RSP_0482–80) and carbohydrate kinase (RSP_0490), as well as substrate transport (RSP_0487–3 and RSP_3168–5) (Fig. 6, S1 Table (cluster 83)), making it functionally analogous to UxaR [76]. We tested these predictions by comparing RNA levels between wild type (WT) and *ΔRSP_0489* cells, and conducting ChIP-seq analysis with a myc-tagged version of RSP_0489 (Fig. 7). A total of 55 genes were DE (1.5 fold change, pvalue < 0.05) between WT and *ΔRSP_0489* cells, including predicted targets *uxuA*, *kduID-uxuB*, *uxaC* and RSP_0487–3, which were repressed in the presence of RSP_0489 by as much as 36-fold (Fig. 7A, S4 Table). Several other genes involved in substrate transport and metabolism were also DE in this data set (Fig. 7A, S4 Table). ChIP-seq analysis with a 3X myc tagged variant of RSP_0489 revealed that RSP_0489 binds at the promoters for *uxuA* (RSP_0773), the *uxaC* operon (RSP_0488–0), RSP_0489, RSP_0490 and within the coding regions of substrate transporter (RSP_3372–70 and RSP_2667–3) (Fig. 7B, Table 3), verifying several predictions from our TRN model. Overall 4 out of these 6 RSP_0489 target operons (~67%) were correctly predicted in our TRN. The conserved DNA sequence motif derived from sites bound by RSP_0489 also showed similarities to that obtained from phylogenetic footprinting analysis of the RSP_0489 promoter (Fig. 7C). Other genomic locations enriched for RSP_0489 but with no corresponding DE genes are listed in S5 Table.

**Table 3:** RSP_0489 direct targets identified by ChIP-seq and expression profiling.

	ID	Annotation	chrID	peakStart	peakStop	FC^a^	Motif	Expr^b^
1	RSP_0488–80*	*uxaC-kduID-uxuB*	chr1	2222600	2223600	248	TGTCTGA**CTAATATGCTA**GTATGC	-36
	RSP_0489*	GntR family TF	chr1					
2	RSP_0490*	Carbohydrate kinase	chr1	2223800	2224200	135	CGGCGGT**CAGATAGTCCA**CCTCCG	-2.4
3	RSP_0773*	*uxuA*	chr1	2515000	2515799	225	TAATATG**CAAGTATGCCA**GTTTGC	-26
4	RSP_3372–70	TRAP-T transporter	chr2	437000	437600	6	TCGCCCG**CGAATATGTCA**CGCGGG	-2.4
5	RSP_2667–3	ABC transporter	chr1	1310200	1310600	5	CATCGCG**CAGGTATTCCA**GTTTCC	-1.5

* RSP_0489 targets also predicted in TRN

^a^ Fold enrichment of RSP_0489-myc ChIP over control myc antibody ChIP in WT.

^b^ Fold change in gene expression in WT w.r.t ΔRSP_0489.

**Fe-S cluster biogenesis and iron homeostasis**. Genes of the Fe-S biogenesis pathway (*iscSUA-hscBA-fdx*) are regulated by the Rrf2-family TF IscR, in *E*. *coli* and several other bacteria [77,78]. In *E*. *coli*, IscR is a global regulator that is able bind to two different DNA target sequences depending on whether it is ligated to a 2Fe-2S cluster [77,78]. The *R*. *sphaeroides* homolog of IscR, **RSP_0443**, differs from *E*. *coli* IscR as it does not possess cysteine residues required for the ligation to a 2Fe-2S cluster, suggesting that this protein is unable to ligate a Fe-S cluster. If this is true, then the upstream signaling pathway utilized and target genes regulated by RSP_0443 is likely to differ from that of *E*. *coli* IscR.

Consistent with observations in *E*. *coli*, RSP_0443 is predicted in our TRN model to regulate transcription of its own operon (RSP_0443–31). However, the RSP_0443 operon encodes homologs of the Suf Fe-S biogenesis pathway (*sufABCDSE*), which is also a direct IscR target in *E*. *coli* [79]. In addition, RSP_0443 is predicted in our TRN model to regulate transcription of catalase (RSP_2779), bacterioferritin-associated ferredoxin (RSP_1547), imelysin (RSP_1548), biopolymer transport protein TonB-ExbBD (RSP_0920–2), *napEFDABC* (RSP_4112–8), all gene products with predicted Fe-S cluster or heme-binding domains or predicted to be involved in iron uptake (S1 Table (cluster 82)). Thus, members of the predicted RSP_0443 regulon could play a significant role in maintaining cellular iron homeostasis, possibly to provide the metal needed for Fe-S centers. There is also a strong positive correlation between RSP_0443 RNA levels and transcription of its predicted target genes in *R*. *sphaeroides*, suggesting this TF functions as an activator.

**Fig 8:**
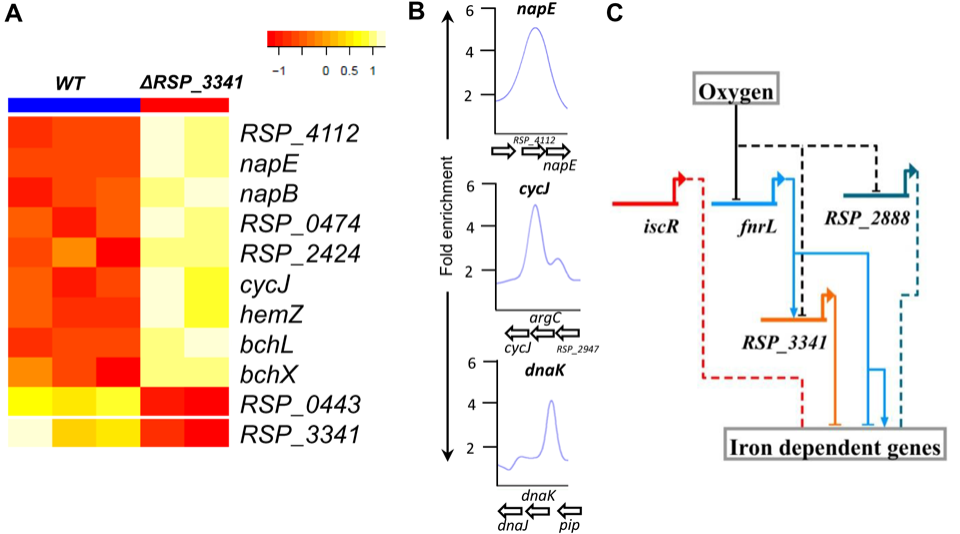
Regulation of iron-dependent genes in *R*. *sphaeroides*. (A) Heat map of iron-dependent DE genes between wild-type (WT) and *ΔRSP_3341* mutant cells from global gene expression analysis. RSP_4112 (hypothetical protein), RSP_0474 (Cytochrome c’), RSP_2424 (ferredoxin II), RSP_2945 (cytochrome c-type biogenesis protein CcmE). (B) Direct binding of RSP_3341 to the *napEFGABC*, *cycJ* and *dnaK* promoters identified by ChIP-seq. (C) Predicted gene regulatory network controlling iron-homeostasis in *R*. *sphaeroides*. Both RSP_2888 and RSP_3341 are RirA like proteins with C-terminal cysteine residues potentially capable of binding Fe-S clusters and sensing oxygen. Solid lines indicate experimentally verified interactions, while dashed lines indicated predicted but as yet unverified interactions.

In addition to RSP_0443, FnrL is directly involved in regulating transcription of genes encoding iron transporters such as *feoABC*, as well as a number of Fe-S and heme containing proteins in *R*. *sphaeroides*. Thus, our TRN predicts that RSP_0443 and FnrL both play an important role in regulation of cellular iron homeostasis. Furthermore, FnrL is also predicted in our TRN to directly activate **RSP_3341**, a putative iron binding RirA-like [80] protein in *R*. *sphaeroides*, which in turn is predicted to negatively regulate the putative 4Fe-4S binding nitrate reductase (*napEFDABC*). We tested this prediction by comparing RNA abundance levels between wild type (WT) and *ΔRSP_3341* cells, and via ChIP-seq analysis using a myc-tagged version of RSP_3341 (Fig. 8A). We found a total of 69 genes were DE (2 fold change, pvalue <0.05) between WT and *ΔRSP_3341* cells including several members of the nitrate reductase operon (*napEFDABC*), which were all repressed by RSP_3341 (Fig. 8A, S6 Table). In addition, transcription of genes encoding other iron dependent proteins (such as cytochromes and ferredoxins) were also repressed by RSP_3341 (Fig. 8A, S6 Table). The mRNA level RSP_0443 was 2 fold higher in WT relative to *ΔRSP_3341* cells, suggesting there might be some cross talk between these TFs. We conducted ChIP-seq analysis with a 3X myc tagged version of RSP_3341 and confirmed the direct regulation of *napEFDABC* by this protein, consistent with our gene expression data and TRN model predictions (Fig. 8B). In addition, RSP_3341 binding was found near Hsp70 DnaK (RSP_1173) and *cycJ* (RSP_2945) (Fig. 8B, Table 4). These genes were also DE in our gene expression dataset, thus were considered as additional direct RSP_3341 targets (Table 4). Twenty-two other sites showing significant enrichment for RSP_3341 but for which no genes in those genomic locations were DE are provided in S7 Table. These data verify the prediction of our TRN model of the involvement of RSP_3341 in the direct and indirect regulation of iron-dependent genes in *R*. *sphaeroides*.

**Table 4:** RSP_3341 direct targets identified by ChIP-seq and expression profiling.

	ID	Annotation	chrID	peakStart	peakStop	FC^a^	Expr^b^
1	RSP_1173	Heat shock protein Hsp70 (*dnaK*)	chr1	2941200	2941599	4.4	-2.7
2	RSP_2945	cytochrome c-type biogenesis protein CcmE (*cycJ*)	chr1	1626000	1626599	5.0	-2.5
3	RSP_4112–8*	Nitrate reductase (*napEFDABC*)	plasmidC	79400	79799	5.1	-6.3

* RSP_3341 targets also predicted in TRN

^a^ Fold enrichment of RSP_RSP_3341-myc ChIP over control myc antibody ChIP in WT.

^b^ Gene expression in WT w.r.t ΔRSP_3341.

Another RirA-like protein in *R*. *sphaeroides*, MppG, predicted to be important in regulation of photosynthesis is also involved in the regulation of iron containing proteins such as AppA (RSP_1565) and those involved in bacteriochlorophyll biosynthesis. Thus, the maintenance of iron homeostasis and the transcriptional regulation of genes encoding iron-dependent enzymes appears to involve a complex gene regulatory network in *R*. *sphaeroides* (Fig. 8C).

**Other major cellular sub-networks**. In addition to the sub-networks described above, many others were predicted in our *R*. *sphaeroides* TRN model including networks involved in carbon metabolism, nitrogen metabolism, hydrogen production, DNA repair, flagella biosynthesis and chemotaxis, heat shock and oxidative stress responses, methionine biosynthesis, phosphate transporter and carotenoid biosynthesis (described in S1 Text).

**Links between sub-networks in the *R*. *sphaeroides* TRN**. In addition to the depth and variety of networks captured in our TRN, we also identified several new and interesting links between these predicted sub-networks. For instance, the TRN predicts a previously unrecognized connection between photosynthesis and iron homeostasis in *R*. *sphaeroides*. The photosynthesis regulators MppG, CrpK, and FnrL, are predicted to regulate several iron/heme-dependent and iron transport proteins. Furthermore, FnrL is also predicted to regulate RSP_3341, which we have shown in this work to be directly involved in regulation of other iron-dependent genes. These data suggest that regulation of photosynthesis, which employs several iron-dependent proteins, and iron homeostasis need to be coordinated in *R*. *sphaeroides* to achieve optimal growth under anaerobic photosynthetic conditions.

NtrC, which is predicted in our TRN to be involved in regulation of nitrogen metabolism (S1 Text), is also predicted to control transcription of genes for central carbon metabolism (Fig. 3 and S5 Fig.), suggesting a possible previously unrecognized link between carbon and nitrogen metabolism in *R*. *sphaeroides*. Similar links between carbon and nitrogen metabolism have been identified in *B*. *subtilis* via the global regulator of carbon metabolism CcpA [81]. Our TRN also captures previously known links between sub-networks controlling the response to heat shock, singlet oxygen stress and DNA repair (S1 Text).

While this description of sub-networks is by no means exhaustive, it provides a useful overview of the various functionalities and connections captured in the *R*. *sphaeroides* TRN model. Overall our TRN model captures a significant amount of known transcriptional regulatory interactions in *R*. *sphaeroides*, while predicting a large number of new interactions for this bacterium that are consistent with observations in other organisms. Furthermore, the TRN model also makes a large number of novel predictions unique to *R*. *sphaeroides*, which represent high-quality targets for future experimental verification. In sum, given the high predictive ability of our TRN model for characterized TFs, we propose that it provides an excellent roadmap for future analysis of the *R*. *sphaeroides* TRN and those of related bacteria.

**Fig 9:**
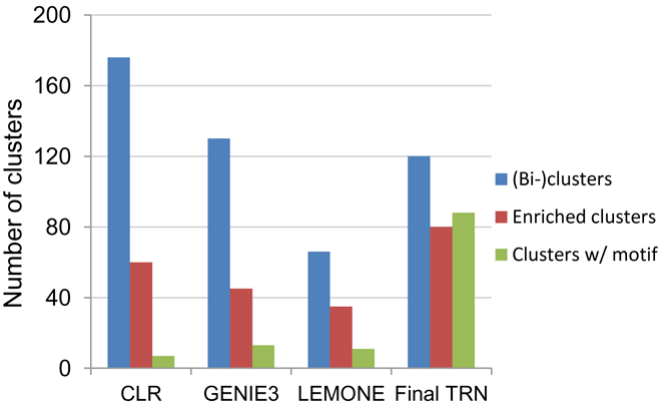
Comparison of predictions from our workflow to those from other inference approaches. Bar chart depicts the number of clusters (or regulons) predicted by CLR, GENIE3, LeMoNe and our approach (Final TRN). It also shows the number of these clusters that are significantly enriched for at least one GO functional category (p <0.00001) and the number of these clusters where we could identify a shared conserved motif using the same *de novo* motif detection approach.

**The integrated TRN inference approach provided significant improvement in information content**. We compared the integrated *R*. *sphaeroides* TRN model to others built from our gene expression compendium using the direct inference approaches CLR [9] and GENIE3 [10], and a module-base inference approach LeMoNe [82]. Selecting networks of similar size (i.e., the top ~1900 predicted TF-target predictions from each approach), we found that our integrated approach generated a TRN with significantly improved information content (Fig. 9). Of the 120 clusters identified in our TRN, 80 (~67%) were enriched for at least one GO functional category compared to 34, 35 and 53% for networks built with CLR, GENIE and LeMoNe, respectively. This comparison suggests our approach captures more functional information. Furthermore, the number of *de novo* detected DNA sequence motifs obtained in the integrated TRN (88 motifs corresponding to ~73% of the clusters), significantly supersedes that obtained by searching the intergenic regions of predicted TF targets obtained from CLR, GENIE and LeMoNe analyses (7, 13 and 11 motifs corresponding to 4, 10 and 17% of the clusters respectively) (Fig. 9). These data suggest that while these expression-based approaches can group potentially functionally related and co-expressed genes together, the resulting clusters likely do not include a sufficiently high percentage of co-regulated genes, so the ability to detect conserved promoter motifs from these predicted clusters/regulons is very low. Thus, it appears that initiating TRN inference with motif detection (via phylogenetic footprinting) prior to incorporating expression data significantly improved its information content and allowed us to overcome some of the limitations in gene expression datasets.

While the regulons of only a handful of TFs have been studied on a genome-scale in *R*. *sphaeroides*, assessing predictions made for some of these TFs highlights other advantages of an integrated approach. For instance, CLR, GENIE and LeMoNe were not able to accurately predict targets for PpsR or FnrL, likely due to the almost invariant expression profiles of these TFs (see Fig. 5B for *ppsR* expression), as their activities are regulated post-transcriptionally. However, by taking other features of bacterial TFs into consideration, we were able to accurately link PpsR and FnrL to their respective regulons, while making predictions across our network for other similarly regulated TFs. On the other hand, for the alternative sigma factor σ^E^ whose binding elements are separated by a variable length spacer region and whose regulon might differ considerably across the species used in our comparative genomics analysis, the expression based approaches performed better at identifying members of this regulon. Thus, incorporating consensus predictions for expression-based inference approaches allowed us to capture such predictions in our final *R*. *sphaeroides* TRN.

Overall, for the 7 TFs for which genome-wide TF-target interaction data exist for *R*. *sphaeroides* (including the 3 TFs analyzed in this study), the predictions from the integrated network outperformed that obtained from expression-based inference approaches, achieving an overall precision (and recall) of 75% (32%), compared to 52% (6%), 74% (12%) and 82% (13%) for CLR, GENIE3 and LeMoNe networks respectively (S8 Table).

### Targets of some TFs remain difficult to identify

Though our approach performed relatively well for many *R*. *sphaeroides* TFs, a large number of verified target genes were not identified for some regulators (e.g., RpoH_I_ and RpoH_II_). This could possibly be due to difficulties in discriminating DNA binding motifs for these or other closely related σ-factors as well as limitations in available gene expression data. Alternatively, it could be the result of constraints used in *de novo* motif detection or limitation of the motif finding algorithm itself. While these constraints performed well at identifying likely binding sites of many traditional TFs, they might be too prohibitive for identification of σ-type motifs.

In addition, of the 81 characterized *E*. *coli* TFs used for performance assessment, accurate predictions could be made for 42 (~52%) of them when considering the predictions from both integrative and expression-based approaches. This leaves a relatively large category of TFs for which available datasets do not provide sufficient information or resolution to make predictions at a reasonable level of precision. Thus, advances in algorithmic and experimental methodologies are still required to bridge this gap.

**Preliminary TRNs for closely related organisms**. An additional benefit of using comparative genomics for TRN inference is that preliminary TRNs can also be built for the other organisms used in the comparative analysis. For instance, the inference of the TRN model for *R*. *sphaeroides* served as the basis for the construction of preliminary sequence-based TRNs for *R*. *sphaeroides* ATCC 17025, *Rhodobacter capsulatus* SB 1003, *Roseobacter denitrificans* Och 114, *Dinoroseobacter shibae* DFL 12, *Rhodopseudomonas palustris* CGA009, *Bradyrhizobium japonicum* USDA 110 and *Paracoccus denitrificans* PD1222 (S9 Table). We expect that these preliminary TRN models will provide insights into the peculiarities of the TRNs of these α-Proteobacteria. They can also serve as starting points for construction of more detailed global TRNs for these and other related bacteria.

### Concluding remarks

In this study, we developed a new workflow to generate genome-scale TRNs, which integrates genome sequence information and gene expression data, as well as taking into consideration properties of bacterial TFs. Validation of this workflow using benchmark datasets for *E*. *coli* showed that it provides significantly improved predictive capability compared to high-performing expression-based approaches. Further analysis of the predicted TRN models showed that the predictions from this workflow and expression-based inference approaches are highly complementary—a feature that could be exploited to build TRN models with greater coverage. We further demonstrated the utility of this workflow by building a large-scale TRN model for *R*. *sphaeroides*. The *R*. *sphaeroides* TRN model consists of 120 gene clusters and 1858 regulatory interactions encompassing ~28% of the genes for this organism. Several observations indicated that this approach generated a large-scale TRN with high predictive power. The majority of the predicted gene clusters were enriched for specific functions and the genes found in many of these clusters were consistent with prior knowledge in *R*. *sphaeroides* or other bacteria. In addition, experimental validation of select *R*. *sphaeroides* TFs showed that the TRN assembled via this integrated approach makes accurate predictions for several of these regulators. Our analysis also illustrates the ability of this workflow to generate of large-scale TRN models with increased information content relative to those built via other approaches. An additional benefit of our approach is that it simultaneously enables construction TRN models for other organisms used in the comparative genomics analysis. Overall, the workflow presented here represents a powerful approach by which to reconstruct TRNs for bacteria for which similar data types are available. It has also provided a large amount of new insight into transcriptional regulation in a phototroph, correctly capturing many aspects of the diverse lifestyles of *R*. *sphaeroides*, while providing novel predictions into regulatory networks that await experimental validation. Thus, this large-scale TRN model should serve as an indispensable data source for those interested in *R*. *sphaeroides* and related bacteria.

## Materials and Methods

### TRN reconstruction

To build large-scale TRN models for *E*. *coli* and *R*. *sphaeroides*, we utilized an approach that combined comparative genomics, gene expression analysis and intrinsic properties of bacterial TFs. The workflow used for our reconstructions is detailed below in a stepwise fashion and summarized in S1 Fig.

**Selecting genomes for phylogenetic footprinting**. Our TRN reconstruction workflow begins with exploiting the sequence information from closely related bacteria [13–15]. In order to identify evolutionarily conserved sequences upstream of homologous genes across multiple species (i.e., phylogenetic footprinting), it is important that relatively closely related species are used, as regulatory mechanisms are more likely to be conserved across these organisms [83]. However, if species are too closely related analysis of upstream sequences becomes uninformative, as large stretches of identical or highly similar sequences prevent the identification of relevant regulatory sequences. Thus, species selected for phylogenetic footprinting analysis were carefully chosen to increase the utility of this approach [12]. To select organisms for our analyses, we used a combination of orthology, phylogeny and physiological information. We considered 3 factors in organism selection: (i) the number of orthologs shared between a given organism and our target organisms, *E*. *coli* and *R*. *sphaeroides* (as a larger number of shared orthologs would enable identification of a potentially larger set of regulatory motifs); (ii) phylogenetic distance (as more closely related species would be more likely to have conserved regulatory mechanisms); and (iii) metabolic diversity (in addition to general cellular processes, we considered the regulation of processes peculiar to these metabolically diverse organisms). Based on these criteria, we restricted the organisms selected for phylogenetic footprinting to those belonging to the orders Rhodobacterales and Rhizobiales for *R*. *sphaeroides*, as these organisms share a larger number of orthologs with *R*. *sphaeroides* (S4 Fig.), are close phylogenetic relatives to *R*. *sphaeroides* (S4 Fig.) and are more metabolically diverse than many members of other α-Proteobacterial orders. From these two orders we selected 8 organisms for our phylogenetic footprinting analysis: *R*. *sphaeroides* 2.4.1, *R*. *sphaeroides* ATCC 17025, *R*. *capsulatus* SB 1003, *R*. *denitrificans* Och 114, *D*. *shibae* DFL 12, *R*. *palustris* CGA009, *B*. *japonicum* USDA 110 and *P*. *denitrificans* PD1222. The criteria used for limiting our analysis to 8 organisms are discussed in the section “Identifying phylogenetically conserved motifs”. For the *E*. *coli* analysis we selected 14 organisms from the Enterobacteriales order based on the same rules: *Escherichia coli* str. K-12 substr. MG1655, *Citrobacter rodentium* ICC168, *Cronobacter sakazakii* ATCC BAA-894, *Dickeya dadantii* 3937, *Escherichia fergusonii* ATCC 35469, *Enterobacter aerogenes* EA1509E, *Erwinia pyrifoliae* DSM 12163, *Klebsiella pneumoniae* CG43, *Pantoea ananatis* AJ13355, *Pectobacterium wasabiae* WPP163, *Salmonella enterica* subsp. enterica serovar Typhi str. CT18, *Shigella dysenteriae* Sd197, *Vibrio cholerae* O1 biovar El Tor str. N16961, *Yersinia pestis* KIM10+. Sequence information for the selected organisms was downloaded from NCBI.

**Identifying orthologous genes between species**. To identify orthologs shared between the selected organisms, we used orthoMCL version 2.0.2 [32]. The blastall function was run with the following parameters:-v 100000-b 100000-F ‘m S’-m 8-e 1e^-5^. All other functions were run with their default settings. Each of the identified orthologous groups (i.e., all orthologs of a given gene across species) was required to have an ortholog from the target species (*E*. *coli* or *R*. *sphaeroides*). For each orthologous group, the intergenic regions (IGRs) greater than 40bp in length, upstream of each gene in the group were then extracted from the appropriate organism, if they existed (genes within operons would generally not contain IGRs of sufficient length). As subsequent motif finding steps would require a sufficient number of sequences to identify meaningful motifs shared by the orthologs, we restricted the orthologous groups carried over to the motif finding step to those having at least 4 IGR sequences. A total of 2162 and 1326 groups of sequences met these criteria and were used for subsequent *de novo* motif detection, for *E*. *coli* and *R*. *sphaeroides*, respectively.

**Identifying phylogenetically conserved motifs**. These groups of intergenic sequences upstream of orthologous genes were used as input for *de novo* motif detection. Motif detection was conducted using MEME [41] with the following parameters:-dna-mod zoops-evt 0.01-nmotifs 3-maxw 30. A third order background distribution file was generated using all the intergenic sequences from all the organisms selected for each analysis and was used to aid subsequent motif detection. A total of 5144 and 914 phylogenetically conserved (PC) *de novo* motifs were detected from these sequences, for *E*. *coli* and *R*. *sphaeroides*, respectively. These were represented PSSMs (S2A Fig.). It should be noted that increasing the number of organism used in our phylogenetic footprinting analysis did not significantly increase the number of identified PC motifs for *R*. *sphaeroides* (S2B Fig.). This analysis also indicated as few as 6 organisms could be sufficient to carry out this analysis, if they possess the appropriate characteristics with respect to the target organism.

**Clustering of identified motifs**. These PC motifs identified in the phylogenetic footprinting step will contain a significant amount of redundancy, as multiple instances of essentially the same motif, corresponding to different binding sites of a specific transcription factor (TF), exist in this set. To eliminate this redundancy from the data set, we grouped identical or very similar motifs into clusters based on their similarity. To achieve this, we first conducted a pair-wise comparison of all identified PC motifs using Tomtom [41,42], generating q-values as measures of the similarity of these motifs to one another. Only motif pairs with q-values <0.01 were considered as potentially identical motifs and retained for subsequent clustering analysis. We then used MAST [41] to identify all the instances of each of the PC motifs (represented as PSSMs) across the target genome. The set of instances identified for a given motif were called “motif groups”. We then conducted a pair-wise comparison of all these motif groups to one another. Motif group pairs showing a high degree of overlap (based on identification of the same motif instances across the genome—threshold set to 33%) and for which the parent motif pairs had a q-value <0.01 were clustered into one group. These clustered motif groups, theoretically contain all the targets for a putative TF within the target genome. The identified target sequences were then used to generate species specific PSSMs based on all instances of each motif identified (see S1 Dataset and S3 Table for *E*. *coli* and *R*. *sphaeroides*, respectively). Based on these analyses, the 5144 and 914 PC motifs were clustered into 225 and 76 unique motifs for *E*. *coli* and *R*. *sphaeroides* respectively, based on their similarity.

**Processing of gene expression data**. Collecting all of the publicly available microarray datasets from *R*. *sphaeroides* from the gene expression omnibus (GEO platform GPL162) (totaling 174 microarrays) and combining these with unpublished microarray experiments conducted in our lab (totaling 24 microarrays), we generated a compendium of 198 microarrays encompassing experiments conducted under a variety of conditions (S1 Dataset), as well as a variety of gene deletion strains (ΔRpoHI, ΔRpoHII, ΔRpoHI/ ΔRpoHII, ΔFnrL, ΔPpsR, ΔPrrA, ΔRSP_4157 and ΔAppA) all constructed in the *R*. *sphaeroides* strain 2.4.1 background. All microarray analyses were conducted on the same Affymetrix platform, circumventing some of the data consistency and normalization issues that can arise when working with heterogeneous data from multiple platforms. All microarrays were normalized together using Robust Multichip Average (RMA) to log_2_ scale with background adjustment and quantile normalization [84]. The RMA normalized data were standardized by row normalization. The normalized *R*. *sphaeroides* gene expression dataset is provided in S1 Dataset. Normalized *E*. *coli* gene expression data was obtained from M3D (http://m3d.mssm.edu/).

**Identifying clusters of co-regulated genes**. Based on the phylogenetic footprinting analysis described above, we identified a total of 225 and 76 clusters of putatively co-regulated genes that shared conserved motifs for *E*. *coli* and *R*. *sphaeroides*, respectively. While these sequence-based networks were rich in information content about co-regulated genes and their putative shared cis-acting regulatory sequences, the information content of such networks could be improved by integration of gene expression data, as genes regulated by the same TFs are likely to have similar transcriptional profiles at least under a subset of conditions [8,12,37]. Hence, a gene which has a predicted shared motif with the other genes in a cluster but does not share a similar transcriptional profile with any other genes in that cluster, over at least a subset of experimental conditions, could be a false positive prediction, which could potentially be filtered out by using expression data. Furthermore, by utilizing bi-clustering algorithms that allow identification of subsets of conditions under which genes are co-expressed, one can potentially determine under what experimental conditions the genes of different clusters are active, providing an indication of their functional roles and/or signals to which they are responsive [12,37].

To integrate the data generated from phylogenetic footprinting with expression datasets, we utilized the data integration frame work DISTILLER [37]. DISTILLER takes in motif information as a binary file indicating whether a particular *de novo* detected motif is present or not. It also takes an expression matrix of normalized expression data across conditions. It then uses an itemset data mining approach to predict what conditions genes sharing a common motif, show correlated expression patterns. We ran DISTILLER on our data sets using the following parameters: binary supports: 1, box supports: 30, box p-values: 0.001, number of randomizations: 100000, size of random modules: 4, minimal module size: 3, number of greedy modules: 400.

The integration of expression data into our predictions resulted in the removal of a subset of genes from the original sequence-based clusters due to an inability to identify sub-conditions under which they are co-expressed with other members of the cluster. For instance, in the case of target genes predicted for cluster 60 in the *R*. *sphaeroides* dataset, eighteen genes were predicted to be members of this cluster based on our phylogenetic footprinting analysis (S7 Fig.), while only 13 of these genes showed strong co-expression with other members of the operon, under at least a subset of conditions. The genes not showing strong co-expression were thus removed from the cluster. Subsequent experimental analysis of the predicted transcriptional regulator for this bi-cluster (PpsR, see Results) verified that these excluded genes were likely false positive predictions from our phylogenetic footprinting step. Thus, at least in subset of instances, integration of our gene expression data sets using DISTILLER appeared to improve the overall accuracy of our TRN.

**Operon extension**. While phylogenetic footprinting analysis enabled us to identify putative binding sites for TFs and thus identify the closest gene(s) to the binding site, other genes within close proximity of this target gene, and potentially in an operon with it, were not captured in the initial analysis. To incorporate operon structure into our predictions, we combined distance-based operon predictions from microbes online [85], with correlation data from the microarray datasets. Genes predicted to be in an operon based on distance and had a Pearson’s correlation coefficient of at least 0.8 across the entirety of our microarray compendium, were considered to be in an operon. This information was used to extend to predictions in our TRN to take into account genes that might be in an operon with targets identified via our sequence-based analysis.

**Prediction of transcriptional regulators for clusters**. Having identified and refined our clusters of co-regulated genes using sequence and gene expression information, we then predicted the most likely of the known or predicted TFs in our target organisms to regulate each of these clusters. To achieve this we used a combination of 4 criteria based on known properties of bacterial TRNs (S3 Fig.). They consisted of:

1. **Correlation** between a TF and its target genes [3,6–8]
2. **Proximity** of a TF to the location of its closest binding site in the genome [12,14,18,19].
3. Similarity in DNA motifs bounds by TFs having similar **DNA binding domains** (DBD) [19,20].
4. **Phylogenetic correlation** of the occurrence of a TF and occurrence of a motif across species [19].

Implementation details of these analyses are provided in the Result and Discussion. The top 3 highest scoring TFs for the *R*. *sphaeroides* network presented in S1 Table are provided in S10 Table, while those for the *E*. *coli* network are provided as part of S1 Dataset.

**Inferring regulatory interactions solely from expression data**. Recent analysis has shown that combining the predictions from a small number of high performing expression-based TRN inference approaches can result in significantly improved prediction accuracy [3]. Thus, to make predictions for TFs not captured in the comparative genomics-based TRN model for *R*. *sphaeroides*, we employed a combination of expression-based TRN inference approaches to try to identify regulatory interactions using only our microarray datasets. For this analysis, we combined the predictions from 3 well-established, high performing direct inference approaches: context likelihood of relatedness (CLR) [9], GENIE3 [10] and ANOVA-based approach [11]. As these approaches have previously been described [3], thus we do not provide any details of implementation or assumptions peculiar to each approach.

Our RMA normalized and row standardized gene expression data from 198 microarray experiments for *R*. *sphaeroides* were used as input data for these 3 inference approaches. A list of 216 *R*. *sphaeroides* TFs was also provided as potential transcriptional regulators (S1 Dataset). In addition, information on specific deleted or over-expressed genes was provided as additional input for ANOVA. The top 50,000 predicted TF-target interactions from each approach were selected. For each inference approach, the scores of TF-target predictions were converted to p-values by random permutation, generating 10000 random TF-target scores for each approach and comparing actual TF-target scores to this set. To determine the likelihood that TF i regulates target gene j, the predictions from each of the 3 approaches for that specific interaction were then combined by averaging the—log10 of the p-values from each approach (eqn. 8). In instances where no prediction was made for a particular TF-target interaction by any one approach, but predicted by at least one of the other 2 approaches, a score of 0 was assigned for that approach. Potential TF-target interactions not in the top 50,000 of any of the 3 prediction lists were not considered.

$$R_{exp}\left(TF_{i},\;Target_{j}\right)\;=\;\frac{1}{3}–log_{10}(P_{CLR}\left(TF_{i},\;Target_{j}\right)*P_{GENIE3}\left(TF_{i},\;Target_{j}\right)*P_{ANOVA}\left(TFi,\;Targetj\right))$$ (8)

Predicted targets for each TF were then extended to include all genes in a potential operon, as described above, to generate the expression-based TRN.

To determine a score threshold to use as a cut-off for interactions to be retained in the *R*. *sphaeroides* expression-based network, we collated all previously generated genome-wide protein-DNA interaction (ChIP) datasets for *R*. *sphaeroides* and used this to generate a precision-recall curve for the network (S8 Fig.). Genome-wide protein-DNA interaction data for FnrL [53], σ^E^ [86], RpoH_I_ and RpoH_II_ [87], corresponding to a total of 467 TF-target interactions, were used for this analysis. We used these interactions as our set of true positives (TP). Precision-recall curves were generated for ranked lists of predictions from CLR, ANOVA, GENIE3 and the combined network (S8 Fig.), with precision and recall calculated at intervals of 100 predictions. Typically precision is calculated as:

$$\frac{TPP}{TPP+FPP}=\frac{True\;positive\;predictions}{True\;positive\;predictions\;+\;False\;positive\;predictions}=\frac{True\;positive\;predictions}{All\;predictions\;made}$$ (9)

where TPP and FPP are assessed based on the number of interactions considered and a gold standard of true positives interactions (TP) [43]. However, due to the small number of TP available for assessment, for each interval of 100 predictions from the *R*. *sphaeroides* expression-based TRNs, we only considered predictions for TFs for which we had ChIP data and thus we redefined precision as follows:

$$\begin{array}{l} \frac{TPP}{TPP+FPP}=\frac{TPP\;for\;TFs\;with\;ChIP\;daTa}{TPP\;for\;TFs\;with\;ChIP\;daTa+FPP\;for\;TFs\;with\;ChIP\;daTa}\\ =\frac{TPP\;for\;TFs\;with\;ChIP\;daTa}{All\;predictions\;made\;for\;TFs\;with\;ChIP\;daTa} \end{array}$$ (10)

Recall was calculated as previously described [43]:

$$\frac{TPP}{TP}=\frac{True\;positive\;predictions}{All\;known\;true\;positives\;}$$ (11)

We selected a precision cut off of 95%, which corresponded to a recall of 8% and a score cut-off of 1.3 (S8 Fig.). Using this cut off for the entire set of predicted interactions resulted in a total of 1100 predicted TF-target interactions. In this analysis the best performing of the individual approach selected was GENIE3, whose performance was very close to the final composite TRN, though the predictions retained the final composite network differed significantly from the predictions of any one of the individual networks (S9 Fig.), as predictions supported by at least 2 of the 3 approaches received higher scores.

**Combining sequence-based and expression-based networks**. To leverage the potential complementarity between the reconstructed *R*. *sphaeroides* sequence- and expression-based networks, we merged predictions from the 2 networks giving precedence to predictions made using the comparative genomics-based integrative approach, as these predictions were supported by both sequence and expression data. Thus, for TFs for which predictions were made in both our comparative genomics- and expression-based networks, only the predictions from the comparative genomics-base network were retained in our final combined network. Based on this a total of 641 TF-target interactions from our expression-based analysis were retained in the final combined network. This included a total of 44 TFs. The final set of interactions predicted using expression-based approaches is provided in S11 Table.

**Experimental analysis**. Details of growth conditions, construction of mutants, microarray and ChIP-seq analyses are provided in S2 Text and S12 Table. All microarray and ChIP-seq datasets generated in this study were deposited in GEO under the accession GSE58658.

**Data and software**. The software required to run the workflow is written in JAVA and provided as part of S1 Dataset. In addition, the code together with files for an example run are available at http://dx.doi.org/10.6084/m9.figshare.1249869.

## Supporting Information

S1 Fig Reconstruction workflow. (PDF)

S2 Fig Identification of phylogenetically conserved motifs. (A) As a illustrative example, promoter sequences of orthologs of FnrL across 8 bacteria are used to build an evolutionarily conserved FnrL motif. This was carried out for the promoters of an additional 1325 groups on intergenic sequences for shared orthologs. (B) To determine how the addition of more genomes would affect the results we obtained from our phylogenetic footprinting analysis, we re-conducted this portion of our analysis with an increasing number of organism. The graph depicts the total number of orthologous groups identified (blue boxes) and the total number of PC motifs identified (red boxes) with respect to the total number of genomes used in the analysis ranging from 8 to 20. The organisms utilized for this analysis were: *R*. *sphaeroides* 2.4.1, *R*. *sphaeroides* ATCC 17025, *R*. *capsulatus* SB 1003, *Roseobacter denitrificans* Och 114, *Dinoroseobacter shibae* DFL 12, *Paracoccus denitrificans* PD1222, *Rhodopseudomonas palustris* CGA009, *Bradyrhizobium japonicum* USDA 110, *Sinorhizobium meliloti*, *Ruegeria pomeroyi*, *Jannaschia sp*. *CCS1*, *Mesorhizobium ciceri*, *Azospirillum* sp. B510, *Rhizobium etli*, *Starkeya novella*, *Azorhizobium caulinodans*, *Xanthobacter autotrophicus*, *Methylobacterium chloromethanicum*, *Rhodospirillum rubrum*, *Ketogulonicigenium vulgare*.
(PDF)

S3 Fig Predicting transcriptional regulators of identified clusters. Overview of the 4 criteria used in predicting the most likely TFs to regulate the sequence-based clusters identified in our analysis.
(PDF)

S4 Fig Phylogenetic tree of α-proteobacteria. The phylogenetic tree was constructed by the neighbor-joining method using aligned *gyrB* DNA sequences from 52 representative species of the major genera of α-proteobacteria. Sequence alignment was done using Clustal X and phylogenetic tree generated using phylip. *Escherichia coli* was used as the out group in this analysis. The grey bar next to each organism is a measure of the number of orthologs that organism shares with *R*. *sphaeroides* 2.4.1. The dash red circle corresponds to the number of orthologs *E*. *coli* shares with *R*. *sphaeroides* 2.4.1. Organisms selected for phylogenetic footprinting are identified with a red star. Phylogenetic tree was visualized using iTOL.
(PDF)

S5 Fig Overview of the reconstructed TRN for *R*. *sphaeroides*. A high-level visualization of the TRN constructed for *R*. *sphaeroides* consisting of 1221 nodes and 1858 edges. Some sub-networks consisting of genes and their regulating TFs enriched for different GO functional categories are highlighted. Green edges represent activation; red edges represent repression, while back edges indicate undetermined regulatory control. Cytoscape 3.0.2 was used for network visualization.
(TIF)

S6 Fig qPCR validation of select PpsR binding sites. Predicted and subsequently ChIP-seq verified PpsR sites validated using ChIP-qPCR.
(PDF)

S7 Fig Improving TRN predictions by integrating gene expression data. (A) Heatmap of the gene expression profiles of genes in cluster 60 across our microarray compendium. The genes in black (RSP_0271 to RSP_1556) show a high degree of correlation in their gene expression profiles, while the genes in red (RSP_1449 to RSP_3000) do not show any significant correlation to one another or other members of the cluster. (B) Summary of predictions made for cluster 18, first using phylogenetic footprinting data only and then after integration with gene expression data. Initial members of the cluster such as RSP_1449 and RSP_3000, which do not show any significant correlation in their expression profiles to other members of the cluster, were filtered out via this data integration step. Subsequent experimental validation via ChIP-seq verified these were likely false positives.
(PDF)

S8 Fig Precision-recall curves for expression-based TRN constructed for *R*. *sphaeroides*. Graph depicts precision and recall calculated for interactions for CLR, ANOVA, GENIE3 and the combined network at 100 predictions intervals.
(PDF)

S9 Fig Comparison of the expression-based networks. Venn diagram comparing the top 1100 predictions made using CLR, ANOVA, GENIE3 and the combined expression network. 144 of the final set of predicted interactions were in agreement across all 3 approaches. In addition, 560 of the predictions in the combined network were also in the top 1100 predictions of at least 2 of the selected approaches. There is an overlap of 571, 604 and 518 between the combined network and the GENIE3, CLR and ANOVA networks respectively.
(PDF)

S1 Table Identified clusters and their predicted transcriptional regulators in *R*. *sphaeroides* TRN. (XLSX)

S2 Table Predicted FnrL targets in *R*. *sphaeroides*. (XLSX)

S3 Table Predicted binding sequences of members of each cluster identified by comparative genomics analysis (XLSX)

S4 Table List of genes differentially expressed between WT and ΔRSP_0489 cells under aerobic respiratory conditions in *R*. *sphaeorides*.Fold change (FC) represents genes up/down regulated in WT w.r.t ΔRSP_0489. (XLSX)

S5 Table Other genomic locations enriched for RSP_0489 in *R*. *sphaeroides*. (XLSX)

S6 Table List of genes differentially expressed between WT and ΔRSP_3341 cells under aerobic respiratory conditions in *R*. *sphaeorides*.Fold change (FC) represents genes up/down regulated in WT w.r.t ΔRSP_3341. (XLSX)

S7 Table Other genomic locations enriched for RSP_3341 in *R*. *sphaeroides*. (XLSX)

S8 Table Comparison of predictions from integrated *R*. *sphaeroides* TRN to expression-based approaches. (XLSX)

S9 Table Clusters identified in the 7 other species used in our comparatives genomics analysis. (XLSX)

S10 Table Top 3 predicted transcriptional regulators for each of the identified clusters with phylogenetically conserved motifs (XLSX)

S11 Table TF-target interactions predicted using the consensus predictions from 3 direct inference approaches. (XLSX)

S12 Table Plasmids, strains and primers used in this study. (XLSX)

S1 Text Description of other pertinent sub-networks. (PDF)

S2 Text Details of experimental analysis. PDF file containing details of experimental procedures.
(PDF)

S1 Dataset Data, results and sample code. This dataset consists of 3 folders containing data used for the analysis presented in the main text and a summary of the results from the *E*. *coli* analysis. These folder include: “Ecoli _results”, which contains a summary of the results obtained from the *E*. *coli* analysis; “Integrate_v1.0”, which contains sample code for running the presented analysis (the most up-to-date version of this code can be obtained from the figshare link provided in the Materials and methods); and “Rsp_data”, which contains the *R*. *sphaeroides* gene expression compendium used in our analysis.
(ZIP)
