# Supplementary material for: An Integrated Approach to Reconstructing Genome-Scale Transcriptional Regulatory Networks
Source: PLoS Comput Biol. 2015 Feb 27;11(2):e1004103. doi: 10.1371/journal.pcbi.1004103 (PMC4344238; doi:10.1371/journal.pcbi.1004103)
Supplement: S1 Fig — (PDF) [file pcbi.1004103.s001.pdf]

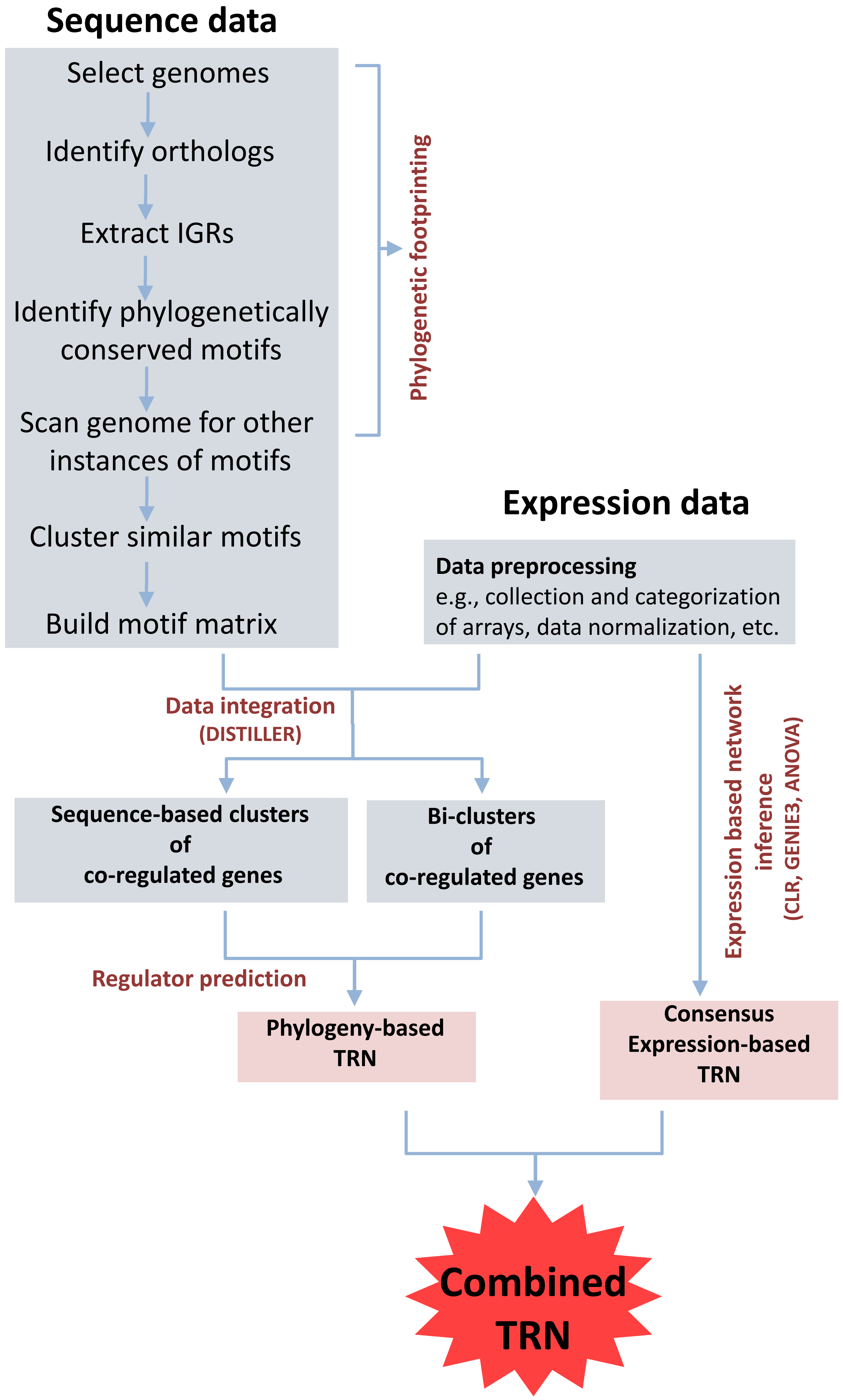
Sequence data
Select genomes
Identify orthologs
Extract IGRs
Identify phylogenetically conserved motifs
Scan genome for other instances of motifs
Cluster similar motifs
Build motif matrix
Phylogenetic footprinting
Expression data
Data preprocessing
e.g., collection and categorization of arrays, data normalization, etc.
Data integration (DISTILLER)
Sequence-based clusters of co-regulated genes
Bi-clusters of co-regulated genes
Expression based network inference (CLR, GENIE3, ANOVA)
Regulator prediction
Phylogeny-based TRN
Consensus Expression-based TRN
Combined TRN
