# Supplementary material for: An Integrated Approach to Reconstructing Genome-Scale Transcriptional Regulatory Networks
Source: PLoS Comput Biol. 2015 Feb 27;11(2):e1004103. doi: 10.1371/journal.pcbi.1004103 (PMC4344238; doi:10.1371/journal.pcbi.1004103)

A.

Organism	Promoter Sequence
R. sphaeroides 2.4.1	CTTTGATTCAGATCAAGC
R. sphaeroides ATCC 17025	CTTTGATTCAGATCAAGC
D. shibae	CCTTGACCCAGATCAATC
R. denitrificans	CTTTGATGTGGATCAAGT
P. denitrificans	CCTTGACCCAAATCAAAT
R. palustris	CCTTGATCTGTGTCAAGC
R. capsulatus	TATTGTCCCAAATCAAGC
B. japonicum	AATTGATCTGGGTCAACC

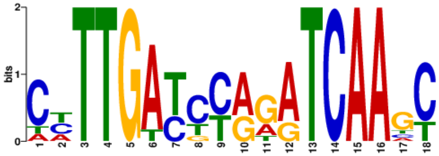

Consensus FnrL promoter motif

B.

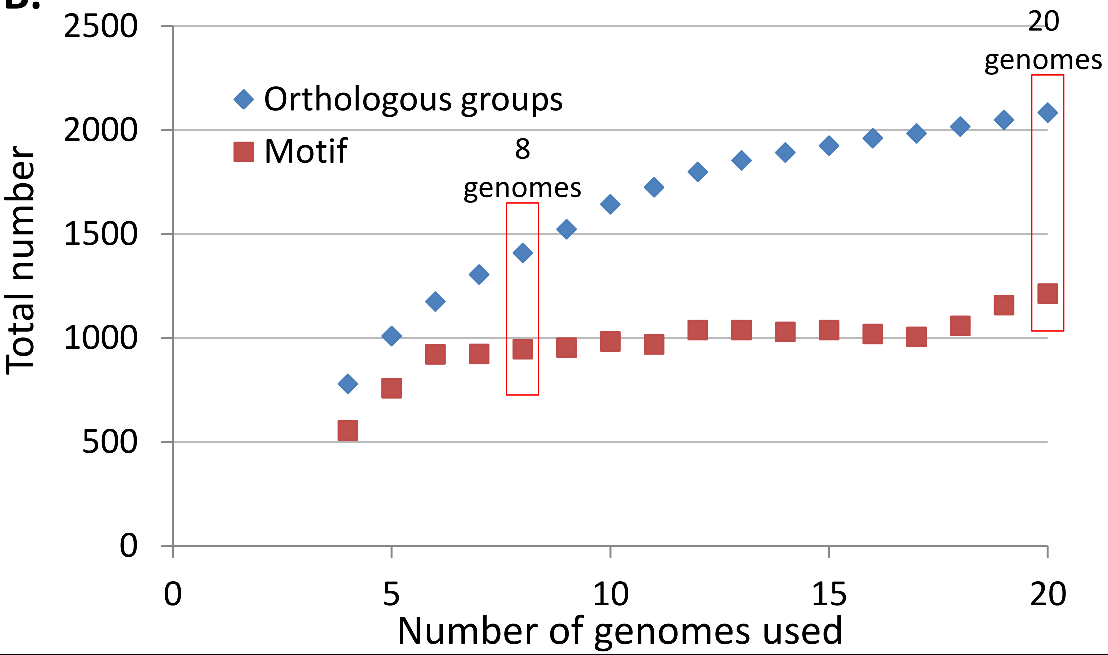

Supplement: S2 Fig — (A) As a illustrative example, promoter sequences of orthologs of FnrL across 8 bacteria are used to build an evolutionarily conserved FnrL motif. This was carried out for the promoters of an additional 1325 groups on intergenic sequences for shared orthologs. (B) To determine how the addition of more genomes would affect the results we obtained from our phylogenetic footprinting analysis, we re-conducted this portion of our analysis with an increasing number of organism. The graph depicts the total number of orthologous groups identified (blue boxes) and the total number of PC motifs identified (red boxes) with respect to the total number of genomes used in the analysis ranging from 8 to 20. The organisms utilized for this analysis were: R. sphaeroides 2.4.1, R. sphaeroides ATCC 17025, R. capsulatus SB 1003, Roseobacter denitrificans Och 114, Dinoroseobacter shibae DFL 12, Paracoccus denitrificans PD1222, Rhodopseudomonas palustris CGA009, Bradyrhizobium japonicum USDA 110, Sinorhizobium meliloti, Ruegeria pomeroyi, Jannaschia sp. CCS1, Mesorhizobium ciceri, Azospirillum sp. B510, Rhizobium etli, Starkeya novella, Azorhizobium caulinodans, Xanthobacter autotrophicus, Methylobacterium chloromethanicum, Rhodospirillum rubrum, Ketogulonicigenium vulgare. (PDF) [file pcbi.1004103.s002.pdf]
