# Supplementary material for: An Integrated Approach to Reconstructing Genome-Scale Transcriptional Regulatory Networks
Source: PLoS Comput Biol. 2015 Feb 27;11(2):e1004103. doi: 10.1371/journal.pcbi.1004103 (PMC4344238; doi:10.1371/journal.pcbi.1004103)
Supplement: S3 Fig — Overview of the 4 criteria used in predicting the most likely TFs to regulate the sequence-based clusters identified in our analysis. (PDF) [file pcbi.1004103.s003.pdf]

A. Correlated expression profile

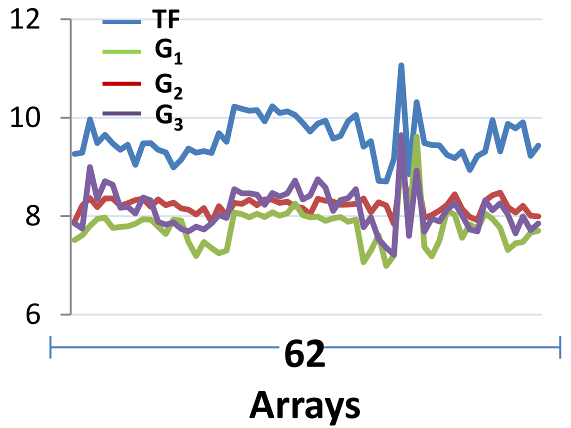

If a transcription factor (TF) is highly correlated to other genes (G) within a cluster, the TF is given a high score for that cluster.

B. TF proximity to cluster motifs

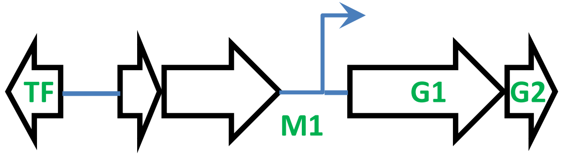

If a transcription factor's (TF) genomic location is close to one (or more) of the motifs (M1) identified in the cluster, the TF is given a high score.

C. Structure of DNA-binding domain (DBD)

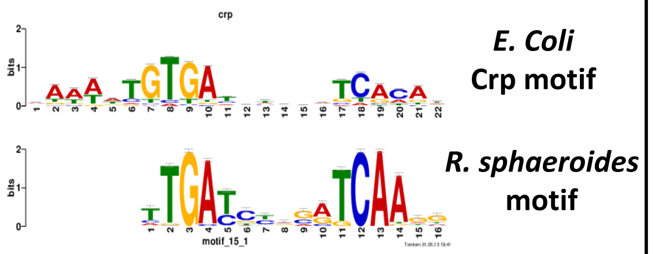

The DBD family of each *R. sphaeroides* TF was determined by Pfam analysis. Then for each identified phylogenetically conserved motif, potential TFs are scored by comparing this motif to the *E. coli* motif of the DBD family to which a given TF belongs. TFs with high similarities get high scores.

D. Phylogenetic correlation

Organisms	Motif occurrence correlation	*TF* occurrence
R. sphaeroides 2.4.1	1	1
R. capsulatus	0.5	1
R. sphaeroides ATCC17025	0.9	1
R. denitrificans	0.6	1
B. japonicum	0	0
P. denitrificans	0	0
R. palustris	0.5	0
D. shibae	0.5	1
**Phylogenetic correlation**		**0.76**

If there is a strong correlation between the occurrence of a TF and a motif across the selected genomes, then the TF gets a high score.
