# Supplementary figures and images for: An Integrated Approach to Reconstructing Genome-Scale Transcriptional Regulatory Networks
Source: PLoS Comput Biol. 2015 Feb 27;11(2):e1004103. doi: 10.1371/journal.pcbi.1004103 (PMC4344238; doi:10.1371/journal.pcbi.1004103)

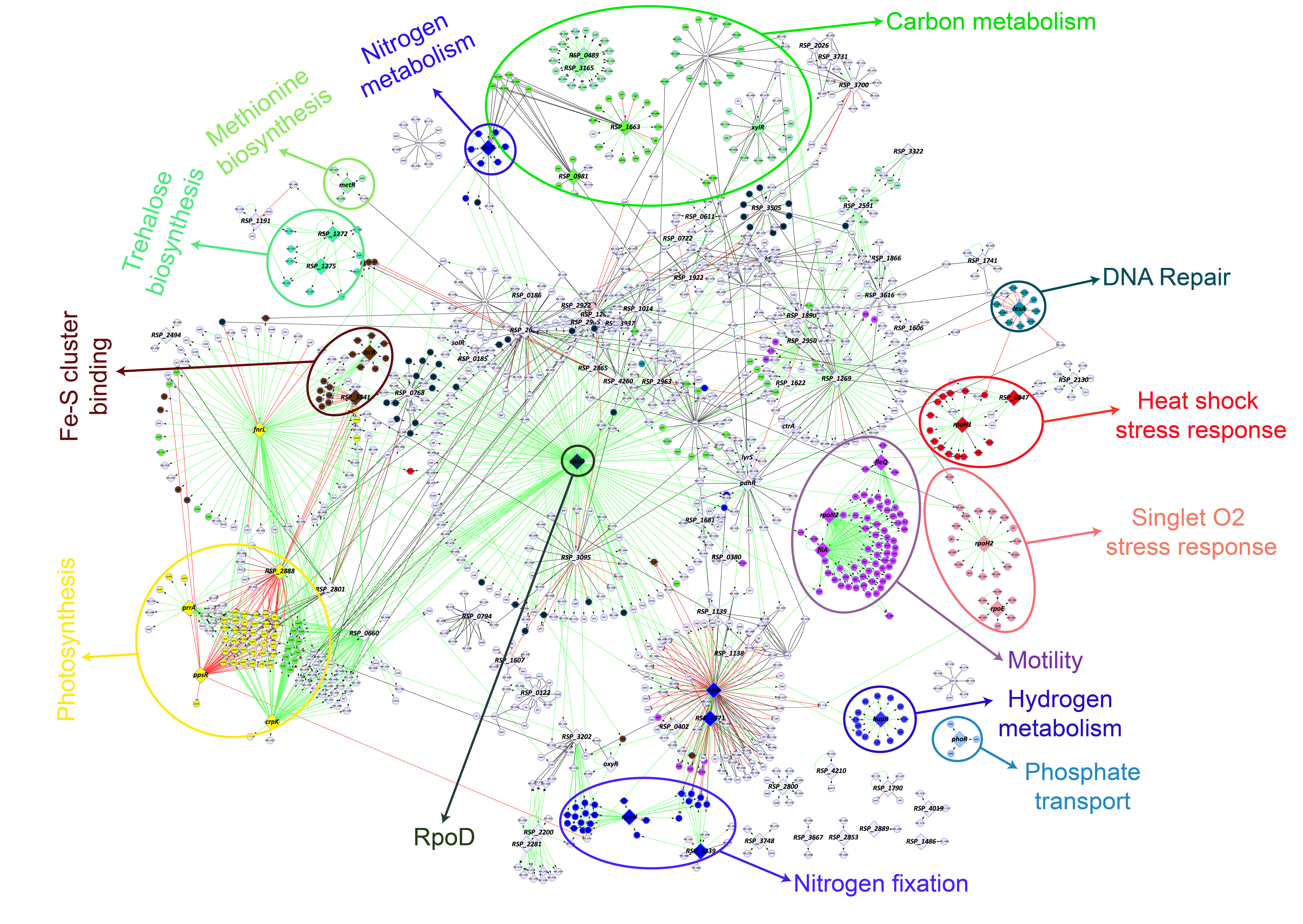

Supplement: S5 Fig — A high-level visualization of the TRN constructed for R. sphaeroides consisting of 1221 nodes and 1858 edges. Some sub-networks consisting of genes and their regulating TFs enriched for different GO functional categories are highlighted. Green edges represent activation; red edges represent repression, while back edges indicate undetermined regulatory control. Cytoscape 3.0.2 was used for network visualization. (TIF) [file pcbi.1004103.s005.tif]

Fold enrichment

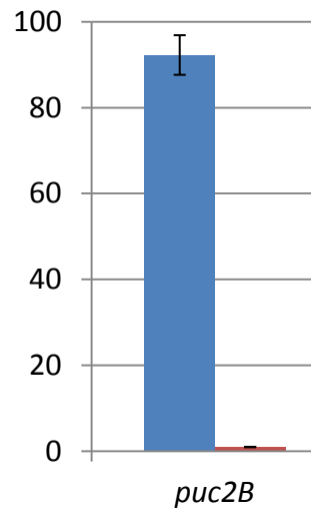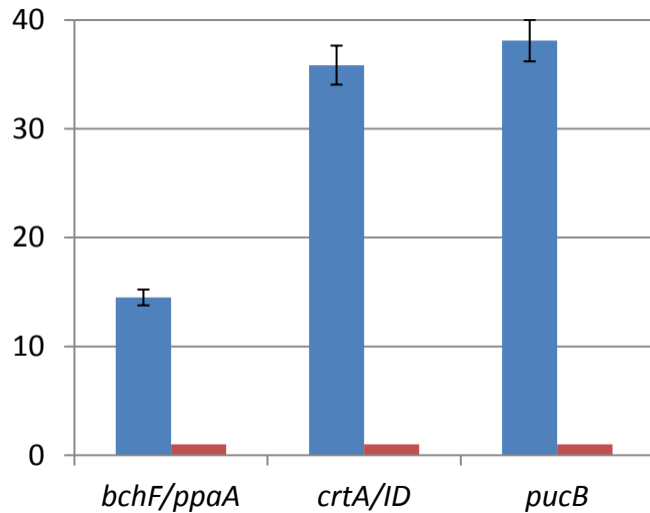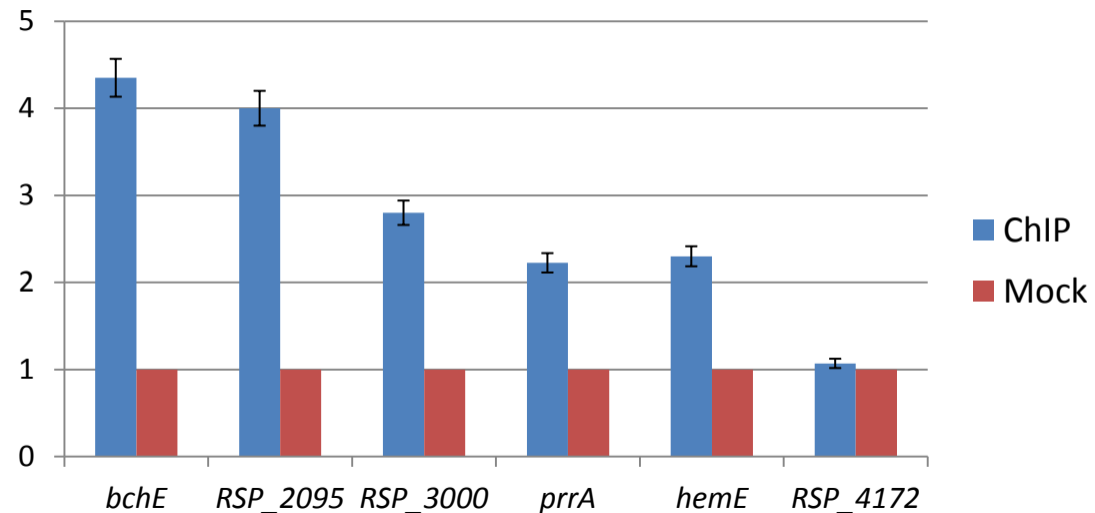

Supplement: S6 Fig — Predicted and subsequently ChIP-seq verified PpsR sites validated using ChIP-qPCR. (PDF) [file pcbi.1004103.s006.pdf]

Precision

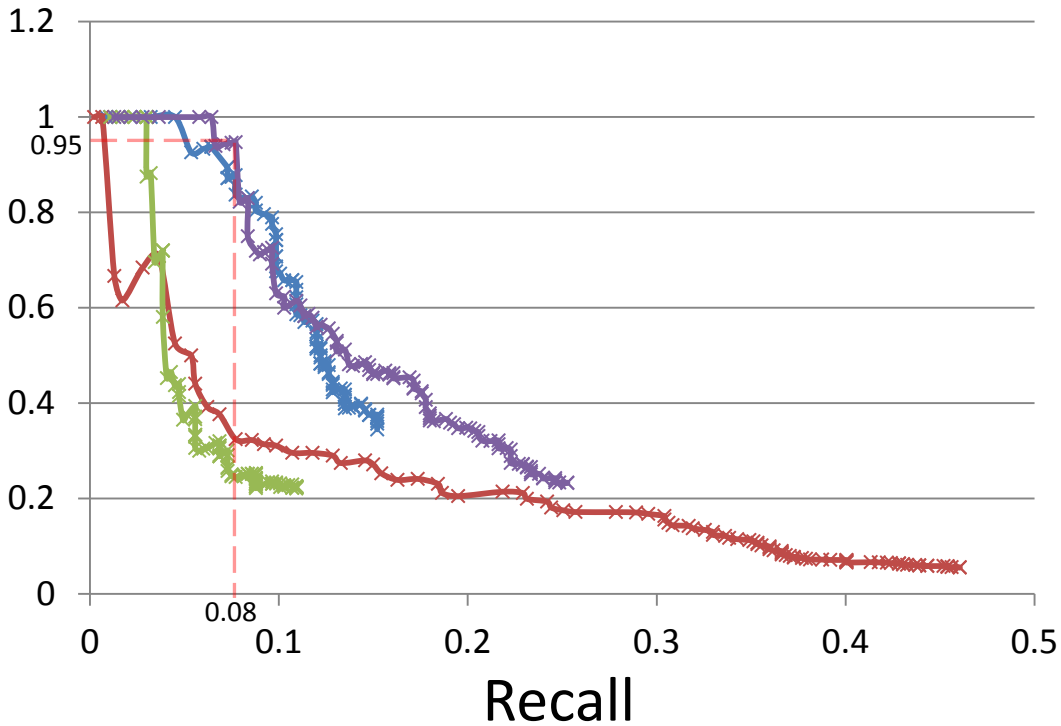

Supplement: S8 Fig — Graph depicts precision and recall calculated for interactions for CLR, ANOVA, GENIE3 and the combined network at 100 predictions intervals. (PDF) [file pcbi.1004103.s008.pdf]

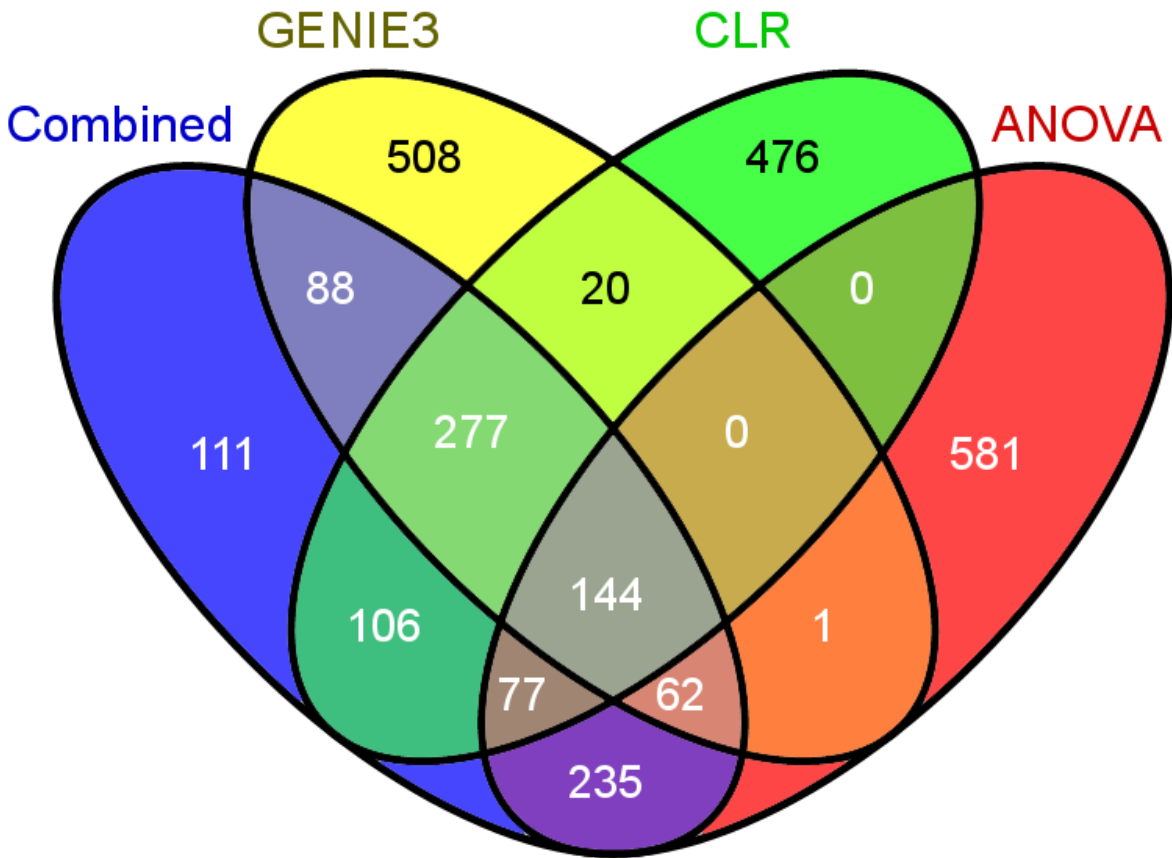

Supplement: S9 Fig — Venn diagram comparing the top 1100 predictions made using CLR, ANOVA, GENIE3 and the combined expression network. 144 of the final set of predicted interactions were in agreement across all 3 approaches. In addition, 560 of the predictions in the combined network were also in the top 1100 predictions of at least 2 of the selected approaches. There is an overlap of 571, 604 and 518 between the combined network and the GENIE3, CLR and ANOVA networks respectively. (PDF) [file pcbi.1004103.s009.pdf]
