# Supplementary material for: An Integrated Approach to Reconstructing Genome-Scale Transcriptional Regulatory Networks
Source: PLoS Comput Biol. 2015 Feb 27;11(2):e1004103. doi: 10.1371/journal.pcbi.1004103 (PMC4344238; doi:10.1371/journal.pcbi.1004103)
Supplement: S7 Fig — (A) Heatmap of the gene expression profiles of genes in cluster 60 across our microarray compendium. The genes in black (RSP_0271 to RSP_1556) show a high degree of correlation in their gene expression profiles, while the genes in red (RSP_1449 to RSP_3000) do not show any significant correlation to one another or other members of the cluster. (B) Summary of predictions made for cluster 18, first using phylogenetic footprinting data only and then after integration with gene expression data. Initial members of the cluster such as RSP_1449 and RSP_3000, which do not show any significant correlation in their expression profiles to other members of the cluster, were filtered out via this data integration step. Subsequent experimental validation via ChIP-seq verified these were likely false positives. (PDF) [file pcbi.1004103.s007.pdf]

A. Expression profiles for Cluster 60

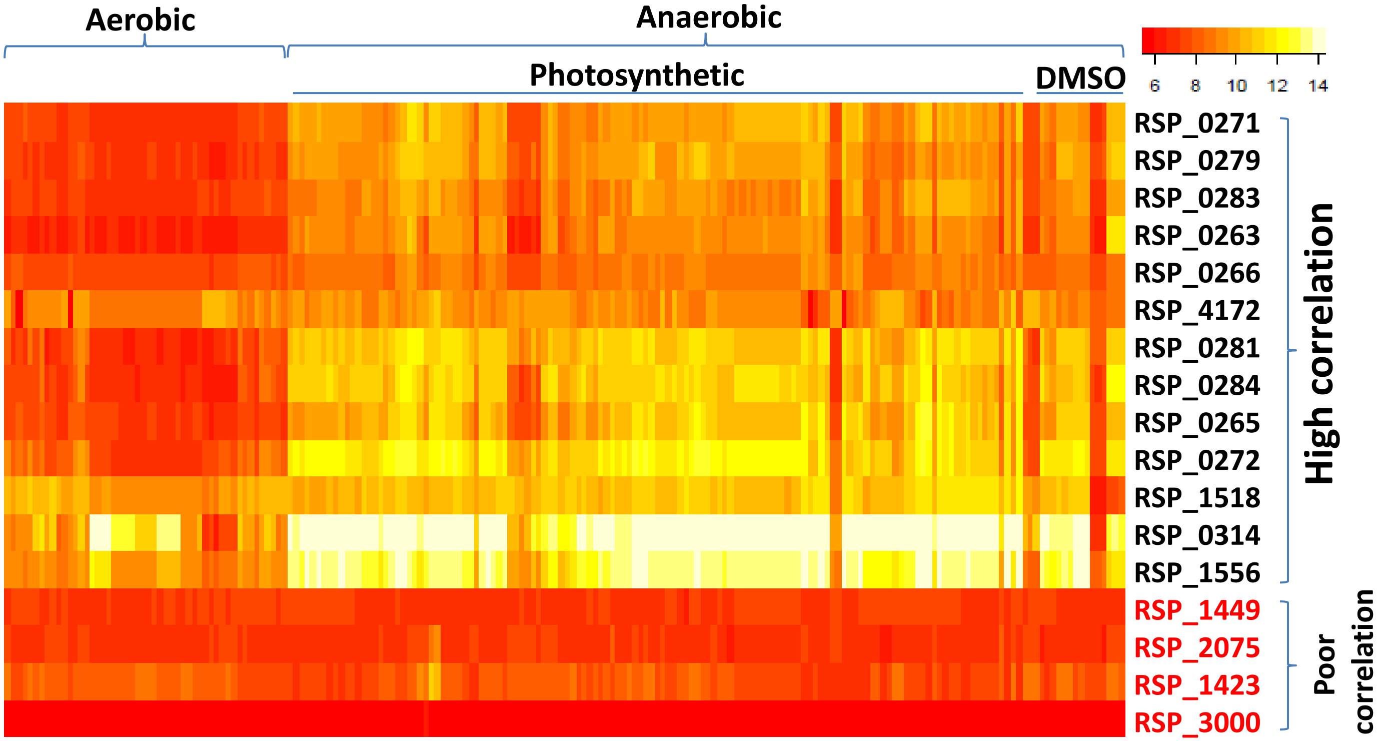

B. Predictions for Cluster 60

Phylogenetic footprinting

ID	Annotation	Verified*
RSP_0263	*bchC*	✓
RSP_0265	*crtE*	✓
RSP_0266	*crtD*	✓
RSP_0271	*crtI*	✓
RSP_0272	*crtA*	✓
RSP_0279	*bchG*	✓
RSP_0281	*bchE*	✓
RSP_0283	*ppaA*	✓
RSP_0284	*bchF*	✓
RSP_0314	*pucB*	✓
RSP_1518	*prrA*	✓
RSP_1556	*puc2B*	✓
RSP_4172	Hypothetical	✗
RSP_1423	ParA family ATPase	✗
RSP_1449	Transcriptional regulator	✗
RSP_2075	Transcriptional regulator	✗
RSP_3000	Hypothetical	✗
RSP_7374	*mvpT*	✗

Phylogenetic footprinting + Bi-clustering

ID	Annotation	Verified*
RSP_0263	*bchC*	✓
RSP_0265	*crtE*	✓
RSP_0266	*crtD*	✓
RSP_0271	*crtI*	✓
RSP_0272	*crtA*	✓
RSP_0279	*bchG*	✓
RSP_0281	*bchE*	✓
RSP_0283	*ppaA*	✓
RSP_0284	*bchF*	✓
RSP_0314	*pucB*	✓
RSP_1518	*prrA*	✓
RSP_1556	*puc2B*	✓
RSP_4172	Hypothetical	✗

*Verified by ChIP-seq and gene expression analysis
