# Supplementary material for: An Integrated Approach to Reconstructing Genome-Scale Transcriptional Regulatory Networks
Source: PLoS Comput Biol. 2015 Feb 27;11(2):e1004103. doi: 10.1371/journal.pcbi.1004103 (PMC4344238; doi:10.1371/journal.pcbi.1004103)
Supplement: S1 Text — (PDF) [file pcbi.1004103.s022.pdf]

Other pertinent sub-networks

Carbon metabolism

Several TFs were predicted to be involved in regulation of carbon metabolism in *R. sphaeroides*. RSP_1663, RSP_0981 and RSP_0489 were already discussed in the main manuscript. Another TF predicted to be involved in regulation of *R. sphaeroides* carbon metabolism is **RSP_1181**, which is annotated as a xylose operon repressor, *xylR*. Consistent with this annotation, RSP_1181 is predicted to regulate a putative xylose ABC transporter (RSP_1178-80), *xylB* (RSP_1177), *xylA* (RSP_1176), as well as its own expression in our TRN. RSP_1181 is also predicted to regulate transcription of L-sorbosone dehydrogenase (RSP_3072), as well as other metabolic and transport proteins, suggesting a potentially broader role for this TF beyond xylose utilization (Fig. 6, S1 Table (cluster 37)). A phylogenetically conserved inverted repeat binding motif (TTTN[A/T]TTTG N CAAA[T/A]NAAA) was identified for this TF (Fig. 6).

RSP_0185 and **RSP_0186** were predicted to regulate genes involved in pyrimidine utilization in *R. sphaeroides* such as allantoate amidohydrolase (RSP_0184), dihydropyrimidine dehydrogenase/glutamate synthase (RSP_0189), as well as their own expression. Both of these TetR family TFs are homologs of *E. coli* RutR, which is known to regulate pyrimidine and purine utilization [1]. Furthermore, the predicted phylogenetically conserved inverted repeat motif for these TFs (T[T/G]ACC N_4 CCT[C/A]AA) is similar to one previously identified for *E. coli* RutR [1] (Fig. 6), indicating that our TRN might accurately capture the regulons of these TFs.

R. sphaeroides lacks enzymes required for the metabolism of acetate via the glyoxylate shunt, but instead utilizes the recently elucidated ethylmalonyl-CoA pathway for this purpose [2]. A large number of the genes encoding enzymes known or predicted to function in the ethylmalonyl-CoA pathway are found in **Cluster 68** (S1 Table, Fig. 6). These include (R)-3-hydroxybutyryl-CoA dehydrogenase (RSP_0747), crotonyl-CoA carboxylase/reductase (RSP_0960), (2R)-ethylmalonyl-CoA mutase (RSP_0961), Malyl-CoA thioesterase (RSP_0970), (2S)-methylsuccinyl-CoA dehydrogenase (RSP_1679) and propionyl-CoA carboxylase (RSP_2189), crotonase (RSP_2305). In addition, the NADH/NADPH transhydrogenase enzyme (RSP_0239-40) is also a member of cluster 68, which might be expected given the large requirement of NADPH for function of the ethylmalonyl-CoA pathway [3]. The members of this cluster are predicted to share a common conserved promoter motif of [G/T][C/A]AA N_5 TT[G/T]C and have a shared co-expression pattern suggesting they are under joint control of a common TF. However, we were unable to link any high scoring TF to cluster 68, so additional experiments are needed to resolve this.

Overall, the predictions of our TRN suggest *R. sphaeroides* uses a robust TRN to control carbon metabolism. This is not surprising, given its ability to utilize at least 68 different carbon sources for growth [3]. Regulation of central metabolism is well studied in *E. coli* [4,5] and *Bacillus*

subtilis [6,7], but not *R. sphaeroides* or other α-Proteobacteria. To fully utilize the properties of *R. sphaeroides* and other bacteria for biotechnological purposes, it is imperative that the gene regulatory networks controlling these crucial aspects of its metabolic network are better understood. Our TRN provides a blueprint to gaining additional understanding of carbon metabolism in *R. sphaeroides* and related bacteria.

Nitrogen Metabolism, Nitrogen Fixation and Hydrogen Production

Many α-Proteobacteria and most PNB are diazotrophs capable of fixing atmospheric nitrogen into ammonia [8]. Consistent with previous observations about the regulation of nitrogen fixation genes in *R. capsulatus* [8], the *R. sphaeroides* Mo-nitrogenase operon, *nifHDK* (RSP_0539-41) and accessory nitrogen fixation genes, *nifXNE* (RSP_0535-38), are predicted in our TRN to be under the joint control of **RpoN (RSP_0527)** and **NifA (RSP_0547)**. In other α-Proteobacteria, NifA is a known transcriptional activator of the nitrogenase structural genes, recruiting RpoN to the promoter of the *nif* operon to stimulate transcription under nitrogen-limiting conditions [8]. A similar positive control mechanism for NifA in nitrogen fixation is predicted in our TRN. In addition to RpoN and NifA, another TF predicted by our TRN to participate in the regulation of the *nif* operons is **RSP_3339** – a GntR family TF. In addition to the *nif* operons, RSP_3339 is also predicted to regulate spermidine/putrescine (RSP_3337-8) and di-/oligopeptide transporters (RSP_3892) (S1 Table (cluster 45)). Thus, RSP_3339 might control nitrogen metabolism in the presence of specific nitrogen sources or under certain nitrogen-limiting conditions. Finally, our TRN predicts that **RSP_3771** – a XRE-family TF, negatively regulates the expression of the *nif* genes functioning antagonistically to NifA and potentially preventing the unnecessary expression of proteins involved in the energetically demanding process of nitrogen fixation.

In addition to the *nif* genes, RpoN is predicted to regulate a small number of other operons including those encoding proteins proposed to transfer electrons to the nitrogenase [8,9] including *rnfABCDGEH* (RSP_3192-9) and the genes in the RSP_3191-88 operon (S1 Table (cluster 5)). The features of the predicted RpoN regulon in our TRN model could reflect either a highly specialized role for this σ factor in *R. sphaeroides* or a limitation of our approach in identifying targets for this transcriptional regulator.

NtrC is predicted in our TRN model to bind a phylogenetically conserved inverted repeat motif of GC N_{11} GC (S1 Table (cluster 1)). NtrC is predicted to regulate transcription of genes encoding proteins involved in maintaining cellular nitrogen homeostasis including GlnB (RSP_0146), GlnK (RSP_0889), AmtB (RSP_0888), dihydropyrimidine dehydrogenase/glutamate synthase (RSP_0189), as well as its own operon that includes genes like NifR3 (RSP_2836) and NtrB (RSP_2837). These predictions are consistent with the known role of NtrC in regulating genes and pathways in response to environmental nitrogen level [8].

The production of H_2 in *R. sphaeroides* is intimately linked to the nitrogen status of the cell, as the nitrogenase enzyme is the major source of H_2 in this bacterium [8-10]. Transcription of the

uptake hydrogenase enzyme, which splits H_2 into protons and electrons, is increased under H_2 producing conditions [11]. H_2 is sensed via HupTUV, while the hydrogenase genes *hupSL* are directly bound by the response regulator HupR in *R. capsulatus* [12]. Consistent with these observations, our TRN predicts that genes encoding subunits of the uptake hydrogenase *hupSL* (RSP_0495-6) and several accessory proteins (RSP_0497-RSP_0509) are under the control of the response regulator, **HupR (RSP_0507)** in *R. sphaeroides* (S1 Table (cluster 84)).

DNA Repair

Similar to other well studied bacteria such as *E. coli* [13,14], the DNA repair machinery in *R. sphaeroides* is predicted in our TRN to be controlled by the **LexA (RSP_1997)** repressor (S1 Table (cluster 59)). Our TRN predicts LexA regulates transcription of *recA* (RSP_0452), putative DNA repair enzyme (RSP_1458), *lexA* (RSP_1997), *uvrD* (RSP_2092), DNA-binding protein (RSP_2234) and *uvrA* (RSP_2966), all genes known or predicted to be involved in DNA repair and which are verified LexA targets either in *R. sphaeroides* [14,15] or other bacteria [13]. Our TRN also predicts that LexA binds to the direct repeat motif GTTC N_7 GTTC, consistent with a previously identified SOS box for *R. sphaeroides* [14].

In addition, our TRN predicts that LexA regulates genes involved in other processes such as class I diheme cytochrome c (RSP_0306), ferredoxin (RSP_0352), aldo/keto reductase (RSP_0423), UDP-3-O-[3-hydroxymyristoyl] N-acetylglucosamine deacetylase (RSP_2115), and several hypothetical proteins (RSP_0424, RSP_1656, RSP_2624, RSP_6187, and RSP_6249). Most of these predicted target genes are likely involved in various aspects of metabolism, suggesting that the functional role of LexA in *R. sphaeroides* might extend beyond DNA repair, as has been reported in some other bacteria [16,17].

Flagella biosynthesis, cell motility and chemotaxis

R. sphaeroides has a single uni-directionally rotating flagellum used for swimming and chemotaxis [18]. The *R. sphaeroides* genome encodes 3 chemotaxis (*che*Op_1 (RSP_2443-33), *che*Op_2 (RSP_1583-89) and *che*Op_3 (RSP_0049-2)) and 2 flagella (RSP_0052-66 and RSP_0083-71) operons [19-21]. Consistent with previous observations [22-24], our TRN predicts that the regulation of flagella biosynthesis and cell motility, as well as chemotaxis, is jointly controlled by 3 TFs: **FliA (RSP_0032), RpoN2 (RSP_0068) and FleQ (RSP_0071)** (S1 Table (clusters 77, 78 and 79)).

FliA and RpoN2 are predicted in our TRN to jointly regulate *che*Op_2 and *che*$Op_{3,}$ as well as both flagella biosynthesis operons. In addition, several genes located outside of the chemotaxis and flagella operons but likely important in motility and chemotaxis were also predicted to be part of this shared regulon, including RSP_3083 (methyl accepting chemotaxis protein), RSP_3302 (chemotaxis response regulator CheY4), RSP_3303 (putative methyl accepting chemotaxis protein McpG) and others (S1 Table). On the other hand, FleQ is predicted to regulate only *che*Op_2 as well as the flagella operon RSP_0052-66, consistent with the previous implication of

FleQ in the regulation of these flagella genes [23]. FleQ is also predicted to regulate genes outside the chemotaxis/flagella operons including RSP_0513 (a putative glycoside hydrolase) and RSP_3432 (methyl-accepting chemotaxis protein).

Interestingly none of the 3 TFs in this motility sub-network was predicted to regulate *che*Op_1. Previous mutational analysis of the *che*Op_1 operon showed its loss had little impact on *R. sphaeroides* motility, unlike *che*Op_2 and *che*Op_3 [18,20],which are required for normal chemotatic responses [19-21]. These results might mean that *che*Op_1 is not expressed under experimental conditions analyzed thus far and might be under the control of a completely different, as a yet unidentified, regulatory program from the other chemotaxis and flagella genes.

Heat Shock and Oxidative Stress Responses

The ability of an organism to survive in nature is also highly dependent on its ability to mount effective responses to various environmental stresses. Previous analysis of two *R. sphaeroides* σ^{32} paralogs, **$RpoH_I$ (RSP_2410)** and **$RpoH_{II}$ (RSP_0601)**, revealed the overlapping but distinct regulons of these σ factors, indicating a convergence in the transcriptional responses to heat shock and singlet oxygen [25,26]. $RpoH_I$, primarily involved in the cellular response to heat shock, directly regulates a large regulon consisting of ~175 target genes involved in a wide variety of functions ranging from protein folding to fatty acid biosynthesis [25]. Our TRN accurately captures a portion of this regulon, identifying several genes directly involved in heat shock response such as HtpX (RSP_0554), a zinc dependent protease HtpX (RSP_2649), RSP_0559, Hsp20 (RSP_1572) and a small heat shock protein (RSP_1016), in addition to several other proteins of varying function previously identified as part of the $RpoH_I$ regulon (S1 Table (cluster 4)) [25]. In total, 15 experimentally verified $RpoH_I$ target operons were identified in our TRN model. In addition, new putative targets such as *recA* (RSP_0452) and *lolA* (RSP_1497) were also predicted as direct targets for $RpoH_I$.

On the other hand, $RpoH_{II}$ expression is induced in response to the presence of the reactive oxygen species singlet oxygen in *R. sphaeroides* [25]. Previous ChIP-chip and expression analysis identified 144 direct targets of $RpoH_{II}$, 45 of which overlap with $RpoH_I$ targets. Consistent with the previously verified regulon for this σ factor, our TRN predicts $RpoH_{II}$ regulates the expression of genes involved in maintenance of the glutathione pool (glutathione peroxidase (RSP_2389), hydroxyacylglutathione hydrolase (RSP_2294), glutathione S-transferase (RSP_1591)) and oxidative DNA damage repair (oxidoreductases (RSP_2314, RSP_3537), RSP_2388), amongst others (S1 Table (cluster 85)). In total 17 verified direct $RpoH_{II}$ targets were identified in our TRN with no new predicted members of this regulon.

While $RpoH_{II}$ regulates transcription of the genes required to amount a stress response to singlet oxygen, **σ^E (RSP_1092)** is the master regulator of this response in *R. sphaeroides* [27]. Previous analysis of the σ^E using ChIP-chip and expression analysis, identified the direct targets of σ^E [28]. Our TRN captured a significant portion of the known σ^E regulon including RSP_1088-91,

RSP_1092-3, *phrB* (RSP_2143) and RSP_1852. However, other target genes like RpoH$_{II}$, RSP_1409 and *cycA* (RSP_0296) were not predicted in our analysis (S1 Table (cluster 90)).

The cellular response to oxidative stress caused by H_2O_2 is modulated by OxyR in many bacteria including *E. coli* and *R. sphaeroides* [29]. Our TRN predicts that *R. sphaeroides* **OxyR (RSP_2780)** regulates its own expression, as well as that of catalase (RSP_2779), a verified direct OxyR target in *R. sphaeroides* [29]. In addition, a ferretin-like protein (RSP_0850) and a TetR family TF (RSP_6137) are predicted to be *R. sphaeroides* OxyR targets (S1 Table (cluster 8)), suggesting that this TF may initiate a transcriptional cascade in this bacterium. OxyR regulation of ferritin has previously been reported in other bacteria [30], where it is proposed to serve as an iron reservoir to prevent Fenton chemistry. While motifs for LysR TFs like OxyR are often difficult to identify, our phylogenetic footprinting analysis allowed us to identify several LysR-type motifs, including a highly conserved motif for OxyR in *R. sphaeroides*, highlighting another advantage of utilizing a comparative genomics approach for TRN reconstruction.

Other major cellular sub-networks

Furthermore, consistent with its role as the housekeeping σ factor, **RpoD (RSP_0395)** is predicted to control a large number of genes within our TRN. Seven different clusters were predicted to be under the control of RpoD, with these clusters being enriched in functions ranging from electron transport to protein synthesis (Fig. S5, clusters 12, 26, 39, 43, 52, 54, 58 – S1 Table), consistent with known global role of RpoD in bacteria. The predicted DNA sequence motifs in all clusters resembled typical -35 -10 motifs bound by RpoD with variations in each motif sufficient to cause them to be split into different clusters. Another σ factor predicted to be involved in the regulation of general processes is **RSP_2681**, a Group IV or ECF family alternative sigma factor. RSP_2681 is predicted to be involved in the regulation of 2 gene clusters that encode proteins involved in protein synthesis and fatty acid biosynthesis (S1 Table (clusters 32 and 57)).

In addition to the above sub-networks, several other predictions of our TRN are either consistent with previous knowledge or represent entirely novel predictions which remain to be verified. For instance, **PhoR (RSP_2599)** is predicted regulate the expression of RSP_2601-3 (phosphate ABC transporter), while **RSP_2800** is predicted to regulate genes encoding proteins involved in pyrroloquinoline quinone biosynthesis PqqEDCB (RSP_0790-3)**. RSP_2801** is predicted to regulate transcription of carotenoid biosynthesis genes *crtEF* (RSP_0265-4) and *crtDE* (RSP_0266-7). On the other hand, **RSP_1243** and **RSP_2963** are predicted to be involved in the regulation of genes involved in fatty acid/PHB biosynthesis, while **RSP_2171 (MetR)** is predicted to regulate methionine biosynthesis (S1 Table).

References

1. Shimada T, Ishihama A, Busby SJ, Grainger DC (2008) The *Escherichia coli* RutR transcription factor binds at targets within genes as well as intergenic regions. Nucleic Acids Res 36: 3950-3955.
2. Alber BE (2011) Biotechnological potential of the ethylmalonyl-CoA pathway. Appl Microbiol Biotechnol 89: 17-25.
3. Imam S, Noguera DR, Donohue TJ (2013) Global insights into energetic and metabolic networks in *Rhodobacter sphaeroides*. BMC Syst Biol 7: 89.
4. Perrenoud A, Sauer U (2005) Impact of global transcriptional regulation by ArcA, ArcB, Cra, Crp, Cya, Fnr, and Mlc on glucose catabolism in *Escherichia coli*. J Bacteriol 187: 3171-3179.
5. Saier MH, Jr., Ramseier TM (1996) The catabolite repressor/activator (Cra) protein of enteric bacteria. J Bacteriol 178: 3411-3417.
6. Blencke HM, Homuth G, Ludwig H, Mader U, Hecker M, et al. (2003) Transcriptional profiling of gene expression in response to glucose in *Bacillus subtilis*: regulation of the central metabolic pathways. Metab Eng 5: 133-149.
7. Goelzer A, Bekkal Brikci F, Martin-Verstraete I, Noirot P, Bessieres P, et al. (2008) Reconstruction and analysis of the genetic and metabolic regulatory networks of the central metabolism of *Bacillus subtilis*. BMC Syst Biol 2: 20.
8. Masepohl B, Hallenbeck PC (2010) Nitrogen and molybdenum control of nitrogen fixation in the phototrophic bacterium *Rhodobacter capsulatus*. Adv Exp Med Biol 675: 49-70.
9. Kontur WS, Ziegelhoffer EC, Spero MA, Imam S, Noguera DR, et al. (2011) Pathways involved in reductant distribution during photobiological H_2 production by *Rhodobacter sphaeroides*. Appl Environ Microbiol 77: 7425-7429.
10. Yilmaz LS, Kontur WS, Sanders AP, Sohmen U, Donohue TJ, et al. (2010) Electron partitioning during light- and nutrient-powered hydrogen production by *Rhodobacter sphaeroides*. Bioenerg Res Volume: 55 - 66.
11. Colbeau A, Vignais PM (1992) Use of *hupS::lacZ* gene fusion to study regulation of hydrogenase expression in *Rhodobacter capsulatus*: stimulation by H_2. J Bacteriol 174: 4258-4264.
12. Dischert W, Vignais PM, Colbeau A (1999) The synthesis of *Rhodobacter capsulatus* HupSL hydrogenase is regulated by the two-component HupT/HupR system. Mol Microbiol 34: 995-1006.
13. Butala M, Zgur-Bertok D, Busby SJ (2009) The bacterial LexA transcriptional repressor. Cell Mol Life Sci 66: 82-93.
14. Fernandez de Henestrosa AR, Rivera E, Tapias A, Barbe J (1998) Identification of the *Rhodobacter sphaeroides* SOS box. Mol Microbiol 28: 991-1003.
15. Tapias A, Campoy S, Barbe J (2000) Analysis of the expression of the *Rhodobacter sphaeroides* lexA gene. Mol Gen Genet 263: 957-965.
16. Oliveira P, Lindblad P (2005) LexA, a transcription regulator binding in the promoter region of the bidirectional hydrogenase in the cyanobacterium *Synechocystis sp.* PCC 6803. FEMS Microbiol Lett 251: 59-66.
17. Oliveira P, Lindblad P (2011) Novel insights into the regulation of LexA in the cyanobacterium *Synechocystis sp.* Strain PCC 6803. J Bacteriol 193: 3804-3814.

18. Armitage JP, Macnab RM (1987) Unidirectional, intermittent rotation of the flagellum of *Rhodobacter sphaeroides*. J Bacteriol 169: 514-518.
19. Porter SL, Warren AV, Martin AC, Armitage JP (2002) The third chemotaxis locus of *Rhodobacter sphaeroides* is essential for chemotaxis. Mol Microbiol 46: 1081-1094.
20. Ward MJ, Bell AW, Hamblin PA, Packer HL, Armitage JP (1995) Identification of a chemotaxis operon with two *cheY* genes in *Rhodobacter sphaeroides*. Mol Microbiol 17: 357-366.
21. Hamblin PA, Maguire BA, Grishanin RN, Armitage JP (1997) Evidence for two chemosensory pathways in *Rhodobacter sphaeroides*. Mol Microbiol 26: 1083-1096.
22. Martin AC, Gould M, Byles E, Roberts MA, Armitage JP (2006) Two chemosensory operons of *Rhodobacter sphaeroides* are regulated independently by sigma 28 and sigma 54. J Bacteriol 188: 7932-7940.
23. Wilkinson DA, Chacko SJ, Venien-Bryan C, Wadhams GH, Armitage JP (2011) Regulation of flagellum number by FliA and FlgM and role in biofilm formation by *Rhodobacter sphaeroides*. J Bacteriol 193: 4010-4014.
24. Poggio S, Osorio A, Dreyfus G, Camarena L (2005) The flagellar hierarchy of *Rhodobacter sphaeroides* is controlled by the concerted action of two enhancer-binding proteins. Mol Microbiol 58: 969-983.
25. Dufour YS, Imam S, Koo BM, Green HA, Donohue TJ (2012) Convergence of the transcriptional responses to heat shock and singlet oxygen stresses. PLoS Genet 8: e1002929.
26. Nuss AM, Glaeser J, Berghoff BA, Klug G (2010) Overlapping alternative sigma factor regulons in the response to singlet oxygen in *Rhodobacter sphaeroides*. J Bacteriol 192: 2613-2623.
27. Anthony JR, Warczak KL, Donohue TJ (2005) A transcriptional response to singlet oxygen, a toxic byproduct of photosynthesis. Proc Natl Acad Sci U S A 102: 6502-6507.
28. Dufour YS, Landick R, Donohue TJ (2008) Organization and evolution of the biological response to singlet oxygen stress. J Mol Biol 383: 713-730.
29. Zeller T, Mraheil MA, Moskvin OV, Li K, Gomelsky M, et al. (2007) Regulation of hydrogen peroxide-dependent gene expression in *Rhodobacter sphaeroides*: regulatory functions of OxyR. J Bacteriol 189: 3784-3792.
30. Rocha ER, Smith CJ (2004) Transcriptional regulation of the *Bacteroides fragilis* ferritin gene (*ftnA*) by redox stress. Microbiology 150: 2125-2134.
