# Supplementary material for: An Integrated Approach to Reconstructing Genome-Scale Transcriptional Regulatory Networks
Source: PLoS Comput Biol. 2015 Feb 27;11(2):e1004103. doi: 10.1371/journal.pcbi.1004103 (PMC4344238; doi:10.1371/journal.pcbi.1004103)
Supplement: S2 Text — PDF file containing details of experimental procedures. (PDF) [file pcbi.1004103.s023.pdf]

Bacterial strains and growth conditions

R. sphaeroides 2.4.1 was used as the parental strain in this study and all mutants were constructed in this background. *E. coli* DH5α was used as a plasmid host and *E. coli* S17-1 was used to conjugate DNA into *R. sphaeroides*. *R. sphaeroides* cultures were incubated at 30 ^{o}C in Sistrom's Minimal Medium (SMM) [1]. When required, the media was supplemented with 1 to 5 μM IPTG and 25 μg/mL kanamycin. *E. coli* cells were grown in Luria Bertani medium at 37 ^{o}C, supplemented with 50 μg/mL kanamycin where needed.

Construction of mutants and expression plasmids

All mutants constructed for this study were in-frame markerless deletions. Regions spanning ~1500 bp upstream and downstream of the target gene were amplified using sequence specific primers containing restriction sites for EcoRI, XbaI or HindIII (S12 Table). These fragments were digested with the appropriate restriction enzymes and ligated into pK18mobsacB plasmid [2], which had been digested with EcoRI and HindIII, by three-way ligation to generate the various gene deletion constructs, which were confirmed by sequencing. The pK18mobsacB-based plasmids were separately mobilized from *E. coli* S17-1 into *R. sphaeroides* strains. Cells in which the plasmid had successfully integrated into the genome via homologous recombination were identified by selection on SMM plates supplemented with kanamycin. These cells were then grown overnight in SMM without kanamycin. Cells that had lost the deletion plasmid (and thus the *sacB* gene) via a second recombination event were identified by growth on SMM plates supplemented with 10% sucrose. Individual gene deletions were confirmed by PCR and sequencing with specific primers (S12 Table).

To construct plasmids for the ectopic expression of 3x Myc tagged proteins, we modified the pIND5 plasmid [3] to include 3 copies of a codon optimized Myc tag (EQKLISEEDL – GAGCAGAAGCTGATCTCGGAGGAGGACCTG) within the pIND5 multiple cloning site. New multiple cloning sites were created to allowing tagging of proteins either C-terminally (NdeI-PstI-NcoI) or N-terminally (BamHI-SalI-BglII-HindIII). Ectopic expression plasmids were made by amplifying the target genes from the genome using sequence specific primers (S12 Table) containing restriction sites for NdeI and BglII, HindIII or BamHI for cloning into pIND5 and NdeI/NcoI or BamHI/HindIII for cloning into pIND5-3xMyc. These DNA fragments were digested with the appropriate enzymes and cloned into pIND5 or pIND5-3xMyc digested with the same enzymes. These plasmids were conjugated from *E. coli* S17-1 into the relevant *R. sphaeroides* strains. Cells which harbored the desired plasmid were identified by selection on SMM plates supplemented with kanamycin.

RNA extraction and microarray analyses

RNA was isolated from exponential phase cultures of *R. sphaeroides* strains that were grown in 500 ml cultures in roux bottles either photosynthetically (bubbling with 95% N_2, 5% CO_2) or aerobically (bubbling with 69% N_2, 30% O_2, 1% CO_2). RNA isolation, cDNA synthesis, labeling

and hybridization to *R. sphaeroides* GeneChip microarrays (Affymetrix, Santa Clara, CA) were performed as previously described [4]. Microarray datasets were normalized by Robust Multichip Average (RMA) to $\log_2$ scale with background adjustment and quantile normalization [5]. Statistical analysis of normalized data to identify DE genes was done using the Limma package [6]. Correction for multiple testing was done using Benjamini-Hochberg correction [7]. All analyses were conducted in the R statistical programming environment (http://www.R-project.org).

Chromatin immunoprecipitation analysis

R. sphaeroides cells were grown either photosynthetically (*ΔRSP_3341*+pIND5-*RSP_3341*-3xMyc and *ΔRSP_0489*+pIND5-*RSP_0489*-3xMyc) or aerobically (*ΔppsR*+pIND5-*ppsR*-3xMyc) in 500 ml cultures in roux bottles with bubbling as described above. Cells were induced with 1 to 5 µM IPTG at inoculation and harvested at mid-exponential phase. Chromatin immunoprecipication was conducted as previously described [8], with polyclonal antibodies against the Myc epitope tag (ab9132, Abcam plc). Immunoprecipitated DNA samples were PCR-amplified, gel purified (size selection ~200bp) and sequenced at the UW Biotechnoloy Center sequencing facility, using the HiSeq 2500 sequencing system (Illumina, Inc). The initial 50bp sequence tags were mapped to the *R. sphaeroides* 2.4.1 genome (ftp://ftp.ncbi.nih.gov/genomes/Bacteria/Rhodobacter_sphaeroides_2_4_1_uid57653/) using SOAP version 2.21 [9], allowing a maximum of 2 mismatches and no gaps.

Peaks were identified using MOSAiCS [10] at a false discovery rate of 0.05. The MOSAiCS analysis was conducted as a two-sample analysis, with ChIP-seq data from either input DNA or ChIP conducted with anti-Myc antibody in the WT strain without a Myc-tagged protein used as a control. Only peaks that were called as significant using both controls (i.e., the intersect of the 2 analysis) were considered as true peaks. Motifs were identified from within peak regions using MEME [11].

References

1. Sistrom WR (1960) A requirement for sodium in the growth of *Rhodopseudomonas spheroides*. J Gen Microbiol 22: 778-785.
2. Schafer A, Tauch A, Jager W, Kalinowski J, Thierbach G, et al. (1994) Small mobilizable multi-purpose cloning vectors derived from the *Escherichia coli* plasmids pK18 and pK19: selection of defined deletions in the chromosome of *Corynebacterium glutamicum*. Gene 145: 69-73.
3. Ind AC, Porter SL, Brown MT, Byles ED, de Beyer JA, et al. (2009) Inducible-expression plasmid for Rhodobacter sphaeroides and Paracoccus denitrificans. Appl Environ Microbiol 75: 6613-6615.
4. Tavano CL, Podevels AM, Donohue TJ (2005) Identification of genes required for recycling reducing power during photosynthetic growth. J Bacteriol 187: 5249-5258.
5. Bolstad BM, Irizarry RA, Astrand M, Speed TP (2003) A comparison of normalization methods for high density oligonucleotide array data based on variance and bias. Bioinformatics 19: 185-193.
6. Smyth G (2004) Applications in Genetics and Molecular Biology 3: Berkeley Electronic Press.
7. Benjamini Y, Hochberg Y (1995) Controlling the False Discovery Rate - a Practical and Powerful Approach to Multiple Testing. Journal of the Royal Statistical Society Series B-Methodological 57: 289-300.
8. Dufour YS, Landick R, Donohue TJ (2008) Organization and evolution of the biological response to singlet oxygen stress. J Mol Biol 383: 713-730.
9. Li R, Yu C, Li Y, Lam TW, Yiu SM, et al. (2009) SOAP2: an improved ultrafast tool for short read alignment. Bioinformatics 25: 1966-1967.
10. Kuan PF, Chung D, Pan G, Thomson JA, Stewart R, et al. (2011) A statistical framework for the analysis of ChIP-seq data. Journal of the American Statistical Association 106: 891-903.
11. Bailey TL, Boden M, Buske FA, Frith M, Grant CE, et al. (2009) MEME SUITE: tools for motif discovery and searching. Nucleic Acids Res 37: W202-208.
