# Supplementary material for: An Integrated Approach to Reconstructing Genome-Scale Transcriptional Regulatory Networks
Source: PLoS Comput Biol. 2015 Feb 27;11(2):e1004103. doi: 10.1371/journal.pcbi.1004103 (PMC4344238; doi:10.1371/journal.pcbi.1004103)
Supplement: S1 Dataset — This dataset consists of 3 folders containing data used for the analysis presented in the main text and a summary of the results from the E. coli analysis. These folder include: “Ecoli _results”, which contains a summary of the results obtained from the E. coli analysis; “Integrate_v1.0”, which contains sample code for running the presented analysis (the most up-to-date version of this code can be obtained from the figshare link provided in the Materials and methods); and “Rsp_data”, which contains the R. sphaeroides gene expression compendium used in our analysis. (ZIP) [file pcbi.1004103.s024.zip › S1 Dataset/Ecoli_results/Ecoli_Summary_final.pdf]

| Cluster ID | Genes in cluster | Motif | Motif_Evalue | Enriched functional categories | Top predicted regulators | Best hit RegulonDB |
|---|---|---|---|---|---|---|
| **Cluster_1** | b2659, b2661, b2662, b2663, b1111, b1892, b1881, b1882, b1883, b1884, b1885, b1886, b1887, b1888, b1889, b1890, b1891, b2735, b2714, b3905, b3906, b2869, b2241, b2242, b2243, b2240, b2239, b2887, b2886, b2888, b3437, b4476, b4267, b4266, b4268, b0040, b2143, b0041, b0042, b0043, b0044, b3599, b2343, b3598, b3597, b2736, b2737, b2774, b2769, b2770, b2771, b4463, b4620, b2775, b0459, b0678, b0676, b0677, b4353, b4352, b4354, b2800, b2799, b2463, b2150, b2148, b2149, b0825, b0700, b1242, b1541, b2715, b3904, b3903, b0824, b0823, b0679, b1617, b1615, b1616, b2913, b0907, b0908, b1795, b3830, b0518, b4572, b0520, b0521, b1139, b0610, b1138, b1137, b1112, b3909, b3364, b4060, b1778, b1777, b1256, b1904, b3260, b3261, b0125, b3831, b2845, b4139, b4140, b2924, b2464, b2465, b0162, b2462, b2457, b2458, b2459, b2460, b2461, b0517, b1779, b1780, b1255, b1254, b1528, b3242, b0802, b2141, b2142, b2140, b2844, b2448, b2449, b2964, b3363, b2830, b2829, b1036, b4061, b1459, b3243, b0048, b4111, b0124, b4030, b1520, b1241, b2801, b2802, b2831, b1594, b2344 | 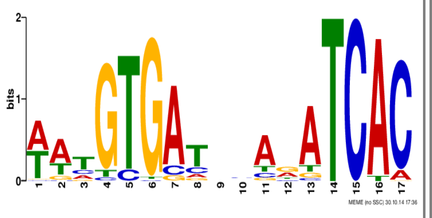 | 8.5e-278 | **GO:0006935** chemotaxis (p=6.7E-12) **GO:0019299** rhamnose metabolic process (p=3.5E-7) | **b3357** (R_score=6.3) **b1334** (R_score=5.6) **b3905** (R_score=3.6) | **crp_1** (qval=0.0000032) **crp** (qval=0.0000032) |
| **Cluster_2** | b2698, b2699, b1275, b3832, b3615, b1144, b1556, b1554, b1555, b1561, b1372, b2882, b2883, b4464, b0606, b1711, b1096, b1097, b1098, b1099, b2176, b3800, b2934, b0074, b0071, b0072, b0073, b3229, b3228, b2450, b4352, b4353, b4354, b1855, b1856, b1857, b1427, b2586, b2414, b2924, b4441, b1370, b2902, b3338, b2456, b2453, b2454, b2455, b0507, b0508, b0509, b3635, b3293, b4550, b3292, b3383, b2019, b2020, b2021, b2022, b2023, b2025, b2026, b1206, b3989 | 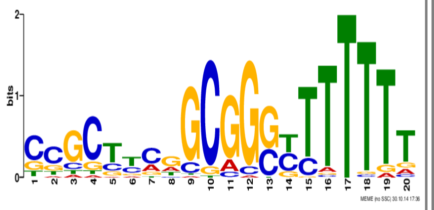 | 1.2e-124 | **GO:0000105** histidine biosynthetic process (p=1.0E-13) **GO:0008652** cellular amino acid biosynthetic process (p=4.5E-10) | **b1892** (R_score=5.6) **b2697** (R_score=3.7) **b1275** (R_score=3.6) | |
| **Cluster_3** | b2421, b2422, b2423, b2424, b1922, b1921, b1264, b1260, b1261, b1262, b1263, b2497, b2498, b1050, b1738, b1734, b1735, b1736, b1737, b2028, b1419, b1780, b1779, b0074, b0071, b0072, b0073, b0075, b4597, b0002, b0003, b0004, b3918, b3730, b3729, b0611, b4131, b4130, b4132, b3642, b3989, b1060, b0437, b0438, b0439, b0167 | 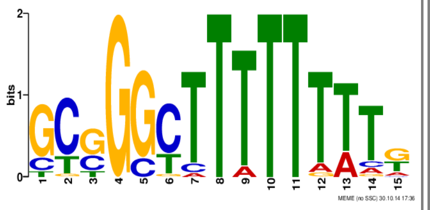 | 1.8e-058 | **GO:1902815** (p=0) **GO:0008652** cellular amino acid biosynthetic process (p=1.1E-13) | **b4133** (R_score=3.6) **b1422** (R_score=3.3) **b1275** (R_score=3.1) | |
| **Cluster_4** | b0894, b0895, b0896, b3094, b1663, b1662, b0750, b0751, b2599, b2598, b1551, b3635, b0171, b4414, b0169, b0170, b0436, b4014, b4015, b1611, b1612, b4441, b2214, b1605, b1606, b3267, b4483, b3833, b3834, b3835, b3838, b3839, b3876, b3877 | 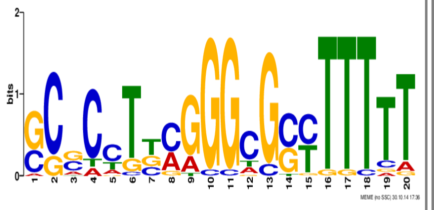 | 2.6e-071 | **GO:0009390** dimethyl sulfoxide reductase complex (p=0) **GO:0004333** fumarate hydratase activity (p=5.1E-7) | **b3884** (R_score=5.0) **b1892** (R_score=4.8) **b3082** (R_score=4.4) | |
| **Cluster_5** | b0592, b4293, b4292, b3071, b4366, b4365, b0150, b0151, b0152, b0153, b1452, b3070, b0584, b0583, b0585, b4511, b0586, b4451, b0593, b0594, b0595, b0596, b0597, b0591, b0590, b0588, b0589, b2155, b1674, b1672, b1673, b2339, b4661, b4582, b3337, b4367, b3908, b0812, b3441, b1451 | 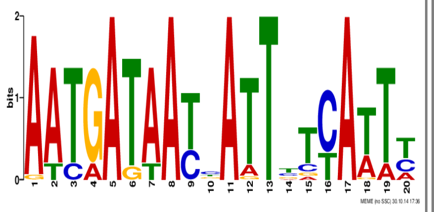 | 1.5e-103 | **GO:0009239** enterobactin biosynthetic process (p=0) **GO:0055072** iron ion homeostasis (p=1.9E-22) | **b0683** (R_score=5.0) **b4293** (R_score=4.5) **b2248** (R_score=4.0) | **fur** (qval=0.0000042) |
| | b3639, b3640, b3641, b0796, b0794, b0795, b4049, b3775, b3606, b0797, b0415, b0413, b0414, b0416, b0417, b1835, b3925, b3778, | | | **GO:0009231** riboflavin biosynthetic process (p=0.0000062) **GO:0008757** | **b3669** (R_score=5.8) **b1914** | |

| | | | | | | |
|---|---|---|---|---|---|---|
| **Cluster_6** | b3638, b0096, b3777 | 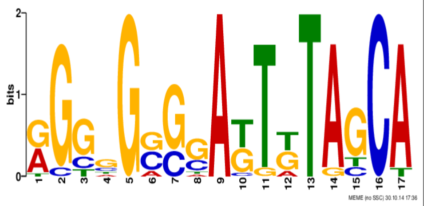 | 1.2e-039 | S-adenosylmethionine-dependent methyltransferase activity (p=0.000013) | (R_score=5.4) **b3461** (R_score=5.3) | |
| **Cluster_7** | b0584, b0583, b3237, b0585, b4511, b0586, b2081, b2305, b0651, b3470, b0503, b4601, b1597, b2244, b3411 | 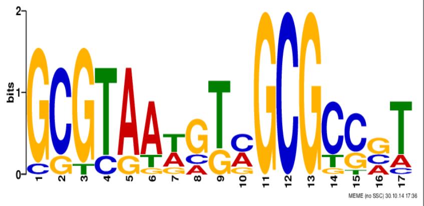 | 3.9e-027 | **GO:0009239** enterobactin biosynthetic process (p=7.8E-9) **GO:0055072** iron ion homeostasis (p=0.00012) | **b2082** (R_score=3.0) **b3405** (R_score=2.7) **b2248** (R_score=2.7) | |
| **Cluster_8** | b0273, b4018, b2669, b4254, b4255, b3172, b0860, b0032, b0033, b3441, b2551, b4019, b3237, b3171, b2018, b2552, b4451, b2062, b2046, b2047, b2048, b2049, b2050, b2051, b2052, b2053, b2054, b2055, b2057, b2058, b2059, b2060, b2061, b3236, b2017, b2016, b4539, b2670 | 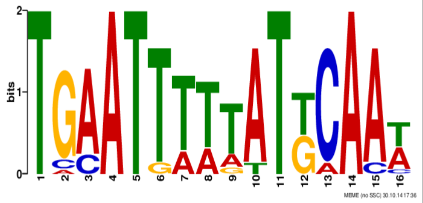 | 9.4e-072 | **GO:0009103** lipopolysaccharide biosynthetic process (p=3.5E-14) **GO:0045228** slime layer polysaccharide biosynthetic process (p=3.6E-13) | **b3237** (R_score=7.4) **b0889** (R_score=4.6) **b3438** (R_score=4.6) | **argr** (qval=0.000088) |
| **Cluster_9** | b1919, b1917, b1918, b1920, b2085, b2086, b0099, b0441, b3698 | 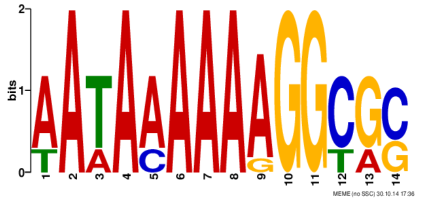 | 8.7e-019 | **GO:0006865** amino acid transport (p=0.00038) | **b0440** (R_score=3.0) **b2082** (R_score=3.0) **b3702** (R_score=2.7) | |
| **Cluster_10** | b4013, b3938, b3941, b3939, b0260, b4012 | 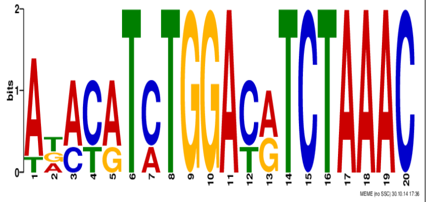 | 1.3e-026 | **GO:0009086** methionine biosynthetic process (p=1.8E-15) **GO:0008652** cellular amino acid biosynthetic process (p=5.9E-8) | **b3938** (R_score=7.6) **b4018** (R_score=2.7) **b3934** (R_score=2.4) | |
| **Cluster_11** | b4236, b3779, b3780, b4348, b4349, b4350 | 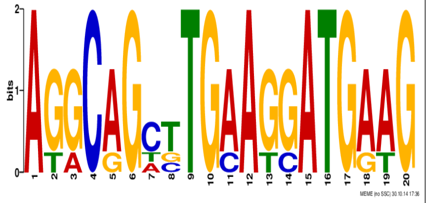 | 5.2e-014 | **GO:0009307** DNA restriction-modification system (p=1.5E-10) **GO:0006304** DNA modification (p=1.7E-9) | **b3346** (R_score=3.3) **b3702** (R_score=3.3) **b3743** (R_score=3.1) | |
| | | | | **GO:0009102** biotin biosynthetic process | **b4401** (R_score=5.8) | |

| | | | | | | |
|---|---|---|---|---|---|---|
| **Cluster_12** | b0774, b2106, b2105, b0775, b0776, b0777, b0778 | 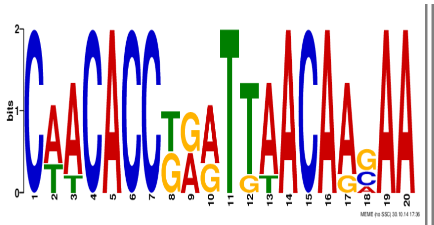 | 4.4e-019 | (p=4.6E-16) **GO:0030170** pyridoxal phosphate binding (p=0.000083) | **b0995** (R_score=5.7) **b0694** (R_score=5.7) | **arca** (qval=0.080) |
| **Cluster_13** | b4237, b3954, b1922, b1921, b2531, b2524, b2525, b2526, b2527, b2528, b2529, b2530, b1508, b1507, b2706, b2702, b2703, b2704, b2705, b2707, b2708, b3190, b3189, b1020, b4512, b0598, b3523, b4068, b1078, b1073, b1074, b1075, b1076, b1077, b1079, b1080, b1081, b1082, b0353, b4096, b4095, b4097, b4098, b4099, b4100, b4101, b4102, b4485, b4227, b4230, b0860, b0674, b1050, b4026, b1973, b3081, b2663, b2659, b2660, b2661, b2662, b2664, b0342, b0343, b3453, b3451, b3452, b2059, b2046, b2051, b2054, b2055, b2056, b2058, b2060, b2061, b2062, b0323, b3587, b3586, b3944, b3167, b3165, b3166, b3168, b3169, b3170, b2522, b3244, b4033, b4032, b4034, b4037, b4323, b4322, b3383, b3382, b4030, b3917, b4107, b4145, b2452, b2451, b2131, b2128, b2129, b2130, b3902, b3901, b2045, b2044, b2924, b2235, b2234, b2236, b3725, b3724, b3726, b3727, b3728, b4259, b4258, b4260, b0891, b0892, b2972, b2971, b4054, b0583, b0584, b0689, b3070, b4016, b3543, b3540, b3541, b3542, b4104, b4106, b3869, b3956, b3937, b0726, b0727, b0728, b0729, b2812, b3603, b3604, b3605, b2958, b3083, b2096, b2092, b2093, b2094, b2095, b3698, b2313, b2312, b0223, b1846, b0028, b0025, b0026, b0027, b0029, b4180, b0038, b4620, b3960, b3958, b3959, b2762, b2763, b2764, b2421, b2422, b2423, b2424, b2425, b1956, b3600, b3601, b1109, b4218, b3408, b3409, b4369, b3071, b4381, b4382, b4383, b4384, b3706, b4160, b4161, b3136, b0333, b0334, b0335, b2681, b0061, b0228, b4087, b4084, b4085, b4086, b4088, b1300, b1301, b1302, b0808, b3813, b0781, b0782, b0783, b0784, b0785, b2277, b2276, b2278, b2279, b2280, b2281, b2282, b2283, b2284, b2285, b2286, b2287, b2288, b0336, b0337, b3673, b0786, b2803, b2804, b0681, b2322, b0780, b3496 | 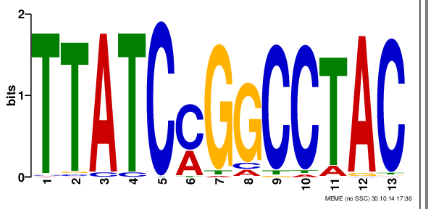 | 7.5e-463 | **GO:0015415** phosphate ion transmembrane-transporting ATPase activity (p=0) **GO:0050136** NADH dehydrogenase (quinone) activity (p=0) | **b1508** (R_score=7.6) **b3555** (R_score=6.0) **b4390** (R_score=6.0) | |
| **Cluster_14** | b2699, b2698, b4043, b4044, b4618, b1183, b1184, b4058, b3813, b3645, b0958, b1848, b1847, b0231, b0232, b0233, b0234, b0799, b2616, b4059, b1061, b0060, b1728, b1182, b0959, b0779, b0227, b4613, b2009, b1176, b1174, b1175, b1849, b1177, b0226, b0225, b0581, b0798, b4616, b1741 | 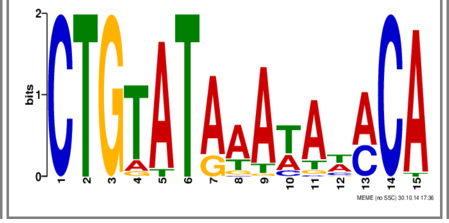 | 1.1e-105 | **GO:0009432** SOS response (p=0) **GO:0006281** DNA repair (p=1.5E-16) | **b4043** (R_score=8.7) **b3237** (R_score=3.6) **b1146** (R_score=3.2) | **lexa** (qval=1.1E-10) **argr** (qval=0.020) |
| **Cluster_15** | b4237, b1020, b4512, b0598, b3523, b4068, b1078, b1073, b1074, b1075, b1076, b1077, b1079, b1080, b1081, b1082, b1922, b1921, b0353, b4096, b4095, b4097, b4098, b4099, b4100, b4101, b4102, b4485, b4227, b4230, b0860, b0674, b1050, b4026, b1973, b3081, b2663, b2659, b2660, b2661, b2662, b2664, b0342, b0343, b3453, b3451, b3452, b2059, b2046, b2051, b2054, b2055, b2056, b2058, b2060, b2061, b2062, b0323, b3587, b3586, b3944, b3167, b3165, b3166, b3168, b3169, b3170, b2522, b3244, b3190, b3189, b4033, b4032, b4034, b4037, b4323, b4322, b3383, b3382, b4030, b3917, b4107, b4145, b2452, b2451, b2131, b2128, b2129, b2130, b3902, b3901, b2045, b2044, b2531, b2524, b2525, b2526, b2527, b2528, b2529, b2530, b2924, b2235, b2234, b2236, b3725, b3724, b3726, b3727, b3728, b4259, b4258, b4260, b0891, b0892, b2972, b2971, b4054, b0583, b0584, b0689, b3070, b4016, b3543, b3540, b3541, b3542, b4104, b4106, b3869, b3956, b3937, b0726, b0727, b0728, b0729, b2812, b3603, b3604, b3605, b2958, b3083, b2096, b2092, b2093, b2094, b2095, b3698, b3954, b2313, b2312, b0223, b1846, b1592, b2712, b0681, b0808, b0028, b0025, b0026, b0027, b0029, b4180, b0038, b4620, b3600, b3601, b1109, b4218, b3408, b3409, b4369, b3071, b0061, b0228, b2476, b1294, b1290, b1291, b1292, b1293, b1741, b4490, b1018, b1019, b3925, b2144, b2145, b2943, b3662 | 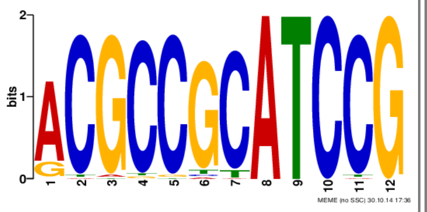 | 9.0e-457 | **GO:0015415** phosphate ion transmembrane-transporting ATPase activity (p=0) **GO:0019700** organic phosphonate catabolic process (p=1.7E-11) | **b3190** (R_score=4.0) **b4018** (R_score=3.6) **b1922** (R_score=3.5) | |
| **Cluster_16** | b2826, b2825, b3889, b3934, b0108, b0106, b0107, b0913, b2761, b3648, b3649, b3650, b3651, b2897, b2896, b3611, b3638, b3639, b3640, b3641, b3612, b3613, b3614, b2898, b4224, b4225, b0959, b0228, b0798, b3766, b2569, b2568, b0019, b0020, b3220, b2798, b0799, b4437, b3890, b1421, b0442, b2244, b0958, b3765, b3647, b3832, b3411, b2536, b2074, b2075, b2076, b2077, b2078, b3523, b3129, b3130 | 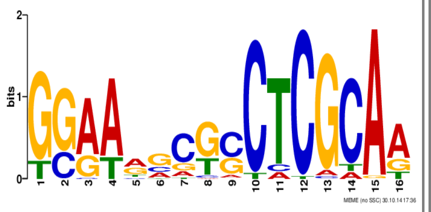 | 2.1e-127 | **GO:0030308** negative regulation of cell growth (p=0.000023) **GO:0015307** drug:hydrogen antiporter activity (p=0.000079) | **b3889** (R_score=3.6) **b3934** (R_score=3.6) **b1422** (R_score=3.4) | |
| | | | | **GO:0006189** 'de novo' IMP | **b1658** | |

| | | | | | | |
|---|---|---|---|---|---|---|
| **Cluster_17** | b2313, b2312, b2476, b2499, b2500, b3654, b0336, b0337, b4311, b1849, b2870, b2871, b2873, b2905, b2903, b2904, b0945, b2498, b2497, b4313, b3653, b1848, b1847, b1185, b1186 | 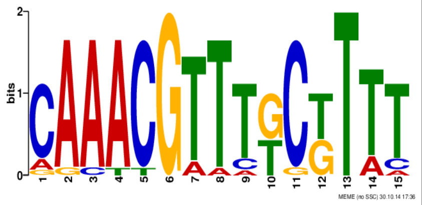 | 1.1e-050 | biosynthetic process (p=2.3E-11) **GO:0006164** purine nucleotide biosynthetic process (p=3.0E-10) | (R_score=6.0) **b0345** (R_score=5.7) **b2837** (R_score=5.4) | **purr** (qval=0.0018) |
| **Cluster_18** | b2551, b1658, b2476, b2557, b1062, b4246, b4244, b4245, b3714, b4602, b2552, b2558, b3715 | 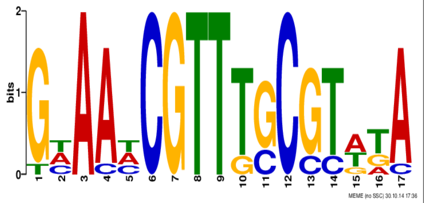 | 1.7e-033 | **GO:0006221** pyrimidine nucleotide biosynthetic process (p=5.8E-11) **GO:0006207** 'de novo' pyrimidine base biosynthetic process (p=1.2E-8) | **b1658** (R_score=8.3) **b4241** (R_score=6.0) **b4264** (R_score=5.6) | **purr** (qval=0.00017) |
| **Cluster_19** | b0674, b0267, b0593, b0594, b0595, b0596, b0597, b0810, b0809, b0811, b4512, b0598, b1741, b2924, b3944, b2903, b2904, b2905, b3600, b3601, b0268, b0269, b3452, b3451, b2902, b3879, b1288, b3725, b3724, b3726, b3727, b3728, b1528, b0592, b0475, b3089 | 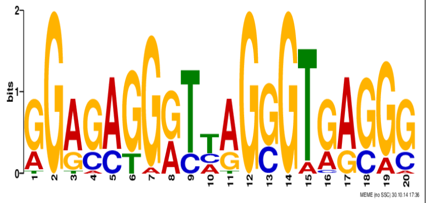 | 3.2e-090 | **GO:0015415** phosphate ion transmembrane-transporting ATPase activity (p=0) **GO:0009239** enterobactin biosynthetic process (p=7.7E-12) | **b0267** (R_score=3.5) **b1526** (R_score=3.3) **b0676** (R_score=3.2) | |
| **Cluster_20** | b2081, b3680, b2562, b4469, b3671, b3670, b3679, b3678, b2889, b2622, b1676, b0910, b0911, b1281, b1282, b1675 | 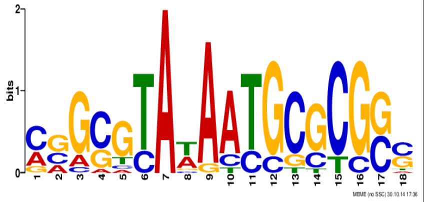 | 1.3e-028 | **GO:0004802** transketolase activity (p=1.9E-7) **GO:0003984** acetolactate synthase activity (p=4.7E-7) | **b2573** (R_score=5.9) **b3067** (R_score=5.5) **b2741** (R_score=5.1) | |
| **Cluster_21** | b3909, b2843, b1675, b1702, b1601, b1703, b3526, b3525, b4252, b1600, b1599, b1627, b1625, b1626, b1628, b1630, b1631, b1632, b1633, b3473, b1901, b4460, b1900, b1902, b3474 | 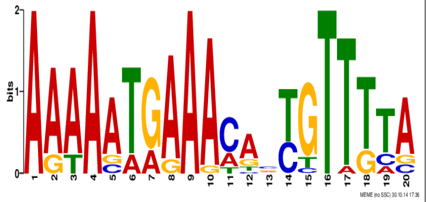 | 1.7e-050 | **GO:0022900** electron transport chain (p=2.3E-11) **GO:0042882** L-arabinose transport (p=1.9E-7) | **b4251** (R_score=2.8) **b3912** (R_score=2.8) **b2847** (R_score=2.7) | |
| **Cluster_22** | b2709, b1988, b1987, b3422, b1303, b2710, b2711, b3421, b1304, b1305, b1306, b1307, b1012, b1006, b1008, b1009, b1010, b1011, b1013, b3870, b3871, b3268 | | 9.1e-042 | **GO:0019740** nitrogen utilization (p=4.1E-17) **GO:0006212** uracil catabolic | **b3202** (R_score=4.7) **b1384** (R_score=4.1) | **sig54** (qval=0.0000011) |

| | | | | | | |
|---|---|---|---|---|---|---|
| | | 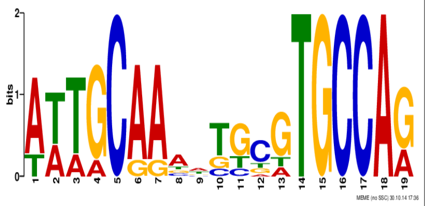 | | process<br>(p=3.3E-16) | **b3906**<br>(R_score=4.1) | |
| **Cluster_23** | b0618, b0613, b0614, b0615, b0616, b0617, b2111, b2109, b2110, b2579, b3158, b3159, b0619, b2580, b3157, b3156 | 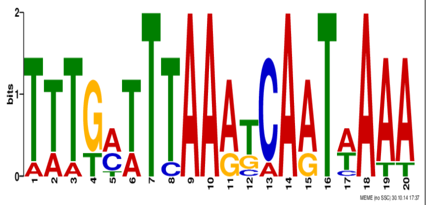 | 1.3e-016 | **GO:0006084**<br>acetyl-CoA metabolic process<br>(p=4.7E-8)<br>**GO:0008080**<br>N-acetyltransferase activity<br>(p=0.000091) | **b3357**<br>(R_score=5.2)<br>**b1334**<br>(R_score=5.0)<br>**b0447**<br>(R_score=3.2) | **fnr**<br>(qval=0.0013)<br>**fnr**<br>(qval=0.0013) |
| **Cluster_24** | b1275, b2925, b2926, b2927, b1826, b1825, b3564, b2958, b4536 | 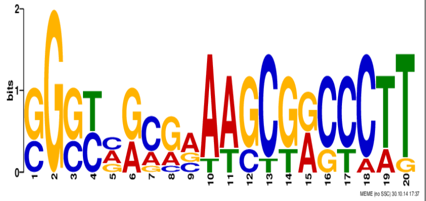 | 1.1e-016 | **GO:0006096**<br>glycolysis<br>(p=0.000012) | **b1892**<br>(R_score=5.2)<br>**b1275**<br>(R_score=3.6)<br>**b1827**<br>(R_score=3.1) | |
| **Cluster_25** | b3756, b3755, b2591, b0201, b3279, b3643, b3321, b3294, b3295, b3296, b3297, b3298, b3299, b3300, b3301, b3302, b3303, b3304, b3305, b3306, b3307, b3308, b3309, b3310, b3311, b3312, b3313, b3314, b3315, b3316, b3317, b3318, b3319, b3320, b2559, b2400, b4006, b4005, b3644 | 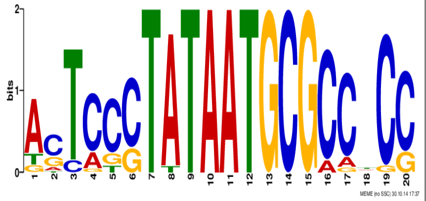 | 2.5e-109 | **GO:0006412**<br>translation<br>(p=0)<br>**GO:0022625**<br>cytosolic large ribosomal subunit<br>(p=0) | **b3755**<br>(R_score=4.0)<br>**b3261**<br>(R_score=3.3)<br>**b2399**<br>(R_score=3.0) | |
| **Cluster_26** | b2601, b2600, b3161, b1906, b2603, b2074, b2075, b2077, b3365, b3366, b2602, b0919 | 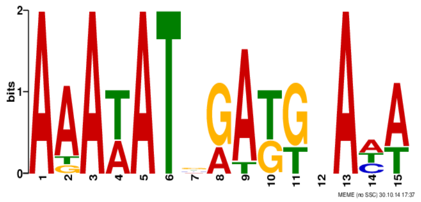 | 2.3e-011 | **GO:0042128**<br>nitrate assimilation<br>(p=0.000021)<br>**GO:0009073**<br>aromatic amino acid family biosynthetic process<br>(p=0.000024) | **b1914**<br>(R_score=2.3)<br>**b3357**<br>(R_score=2.2)<br>**b2079**<br>(R_score=2.1) | |
| **Cluster_27** | b0954, b2323, b2899 | 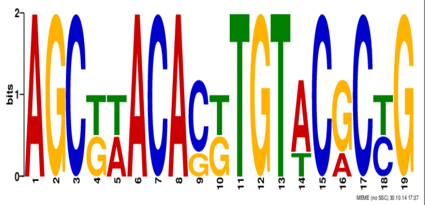 | 1.2e-008 | **GO:0008610**<br>lipid biosynthetic process<br>(p=7.1E-9)<br>**GO:0006633**<br>fatty acid biosynthetic process<br>(p=8.2E-8) | **b2217**<br>(R_score=5.6)<br>**b2369**<br>(R_score=5.5)<br>**b2193**<br>(R_score=5.3) | |
| **Cluster_28** | b0967, b1435, b4374, b4372, b4373, b1866, b1863, b1864, b1865, b0968, b1867, b1868 | | 1.7e-014 | **GO:0016787**<br>hydrolase activity<br>(p=0.00011) | **b1434**<br>(R_score=3.9)<br>**b1438**<br>(R_score=3.1) | |

| | | | | | | |
|---|---|---|---|---|---|---|
| | | 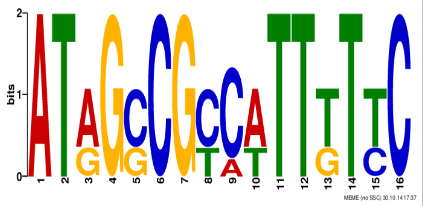 | | | **b4366** (R_score=2.8) | |
| **Cluster_29** | b3870, b0450, b0451, b1988, b1987, b0855, b0854, b0856, b0857, b2310, b2309, b2306, b2307, b2308, b3869, b3868, b0811, b0809, b0810 | 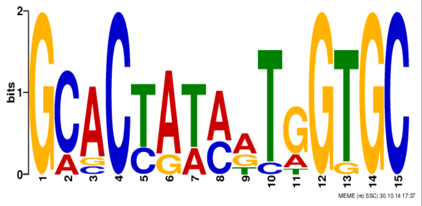 | 4.7e-026 | **GO:0009399** nitrogen fixation (p=0) **GO:0015594** putrescine-importing ATPase activity (p=1.2E-12) | **b3868** (R_score=7.2) **b1987** (R_score=5.3) **b2220** (R_score=5.2) | **glng** (qval=0.00022) |
| **Cluster_30** | b3632, b3623, b3624, b3625, b3626, b3627, b3628, b3629, b3630, b3631, b3438, b2041, b2032, b2033, b2034, b2035, b2036, b2037, b2038, b2039, b2040, b3633, b3634, b2062, b2046, b2047, b2048, b2049, b2050, b2051, b2052, b2053, b2054, b2055, b2057, b2058, b2059, b2060, b2061 | 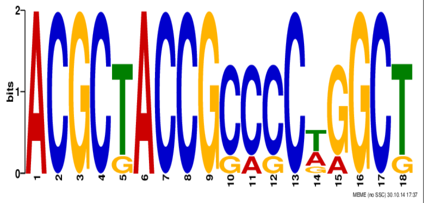 | 1.1e-024 | **GO:0009244** lipopolysaccharide core region biosynthetic process (p=9.5E-20) **GO:0009103** lipopolysaccharide biosynthetic process (p=7.2E-17) | **b3702** (R_score=3.6) **b3190** (R_score=3.0) **b3346** (R_score=3.0) | |
| **Cluster_31** | b2140, b2921, b2156, b1524, b1523, b3778, b2141, b2142 | 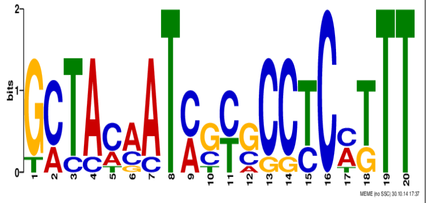 | 5.3e-014 | None | **b3202** (R_score=4.6) **b2921** (R_score=3.8) **b2157** (R_score=2.9) | |
| **Cluster_32** | b0118, b4207, b2828, b2827, b0117, b1807, b4206 | 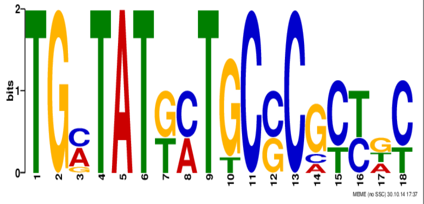 | 1.0e-018 | **GO:0006417** regulation of translation (p=0.0000033) | **b4212** (R_score=3.5) **b0113** (R_score=3.1) **b1799** (R_score=2.4) | |
| **Cluster_33** | b2552, b2732, b4209 | 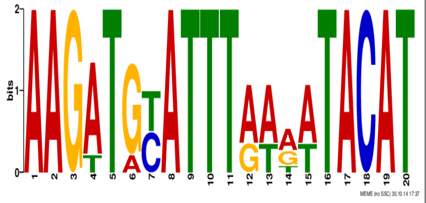 | 3.7e-018 | None | **b4178** (R_score=5.4) **b2735** (R_score=3.1) **b2731** (R_score=3.1) | **nsrr** (qval=0.0032) |
| **Cluster_34** | b2464, b2465, b0897, b2556, b2957, b2463, b0898 | 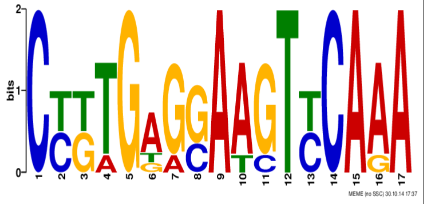 | 7.0e-013 | **GO:0005829** cytosol (p=0.00018) **GO:0003824** catalytic activity (p=0.00032) | **b2554** (R_score=2.6) **b0900** (R_score=2.5) **b2537** (R_score=2.5) | |

| | | | | | | |
|---|---|---|---|---|---|---|
| **Cluster_35** | b0237, b3863, b3862 | 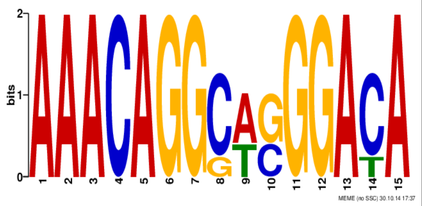 | 4.5e-013 | None | **b3755** (R_score=6.2) **b3872** (R_score=5.9) **b0113** (R_score=5.8) | |
| **Cluster_36** | b0476, b1952, b1953 | 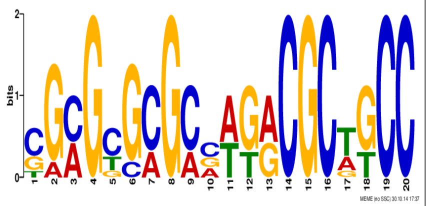 | 7.0e-023 | None | **b0435** (R_score=3.1) **b1951** (R_score=2.6) **b2015** (R_score=2.4) | |
| **Cluster_37** | b3775, b0399, b0400, b2679, b2677, b2678, b3289, b3287, b3288, b3290, b4289, b4287, b4288, b4290, b4291, b0239, b2569, b2568, b2421, b2422, b2423, b2424, b2425, b1370, b0763, b0764, b0765, b1372, b1730 | 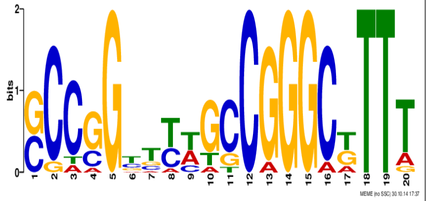 | 5.3e-039 | **GO:0015419** sulfate transmembrane-transporting ATPase activity (p=1.2E-11) **GO:0008272** sulfate transport (p=2.4E-10) | **b1892** (R_score=5.0) **b3261** (R_score=4.8) **b1303** (R_score=3.9) | **flhd** (qval=0.034) |
| **Cluster_38** | b0680, b0399, b0400, b3728, b3724, b3725, b3726, b3727 | 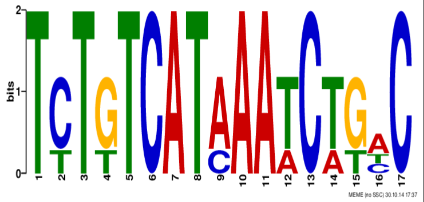 | 9.8e-005 | **GO:0015415** phosphate ion transmembrane-transporting ATPase activity (p=0) **GO:0006817** phosphate ion transport (p=4.2E-24) | **b0399** (R_score=7.7) **b0694** (R_score=5.8) **b2852** (R_score=5.0) | **phob** (qval=0.0000022) |
| **Cluster_39** | b0220, b0219, b2347 | 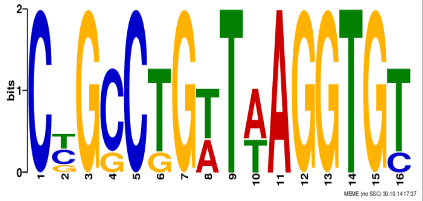 | 8.3e-012 | None | **b0208** (R_score=2.4) **b1739** (R_score=2.1) **b0146** (R_score=2.0) | |
| **Cluster_40** | b3909, b1827, b2843, b1675, b2557, b1676 | 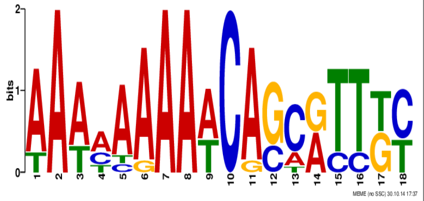 | 2.9e-022 | None | **b1827** (R_score=3.4) **b3906** (R_score=3.1) **b3905** (R_score=2.7) | |
| **Cluster_41** | b3519, b2347, b3518, b2452, b2451, b0607, b0608 | | 2.3e-022 | **GO:0046336** ethanolamine catabolic process | **b3604** (R_score=5.3) **b3684** (R_score=5.0) | |

| | | | | | | |
|---|---|---|---|---|---|---|
| | | 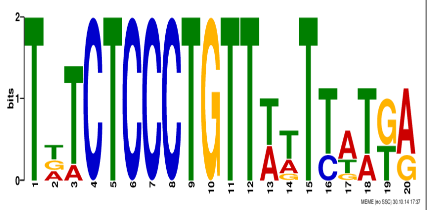 | | (p=0.0000016) | **b1439** (R_score=5.0) | |
| **Cluster_42** | b0419, b1034, b1033, b1035, b2301, b2302 | 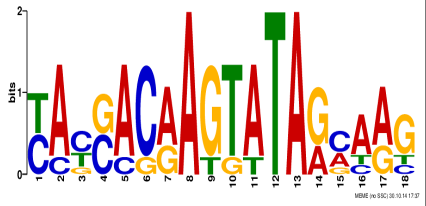 | 1.6e-005 | **GO:0071978** bacterial-type flagellar swarming motility (p=6.8E-9) **GO:0004601** peroxidase activity (p=4.8E-7) | **b1040** (R_score=3.1) **b0435** (R_score=2.9) **b3555** (R_score=2.3) | |
| **Cluster_43** | b4034, b4032, b4033, b4035, b4036, b3417, b3416, b0403 | 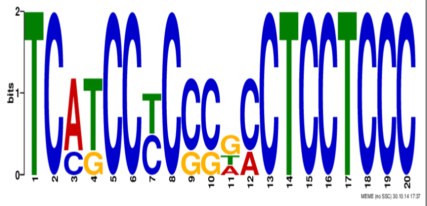 | 5.5e-012 | **GO:0015423** maltose-transporting ATPase activity (p=0) **GO:0042956** maltodextrin transport (p=0) | **b3418** (R_score=8.1) **b3520** (R_score=5.1) **b4365** (R_score=5.1) | **malt** (qval=0.044) |
| **Cluster_45** | b0273, b3574, b4254, b4255 | 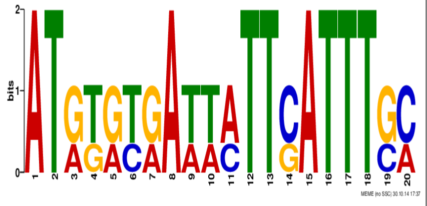 | 1.3e-013 | **GO:0016743** carboxyl- or carbamoyltransferase activity (p=3.4E-9) **GO:0006526** arginine biosynthetic process (p=7.4E-8) | **b3237** (R_score=4.4) **b0272** (R_score=3.2) **b0267** (R_score=2.7) | **argr** (qval=0.0095) |
| **Cluster_46** | b2428, b2427, b2561 | 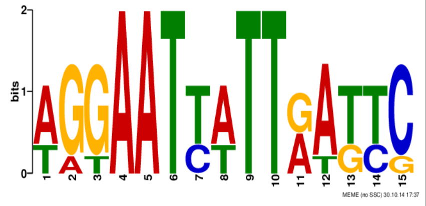 | 8.4e-007 | **GO:0005975** carbohydrate metabolic process (p=0) **GO:0030246** carbohydrate binding (p=0) | **b2427** (R_score=3.7) **b2561** (R_score=3.5) **b2554** (R_score=2.3) | |
| **Cluster_47** | b3493, b1627, b1625, b1626, b1628, b1629, b1630, b1631, b1632, b1633, b3492 | 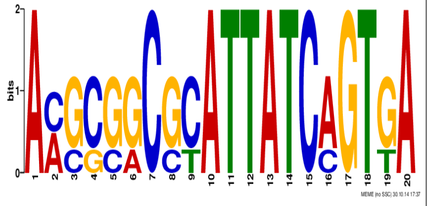 | 9.6e-009 | **GO:0022900** electron transport chain (p=6.1E-14) **GO:0055114** oxidation-reduction process (p=0.0000024) | **b3461** (R_score=6.0) **b3067** (R_score=5.4) **b2573** (R_score=4.8) | |
| | | | | **GO:0005375** copper ion transmembrane | **b0399** (R_score=7.1) | |

| | | | | | | |
|---|---|---|---|---|---|---|
| **Cluster_48** | b4444, b1969, b1968, b1020, b1932, b1970, b0383, b1456, b0572, b0573, b0574, b0575, b0571 | 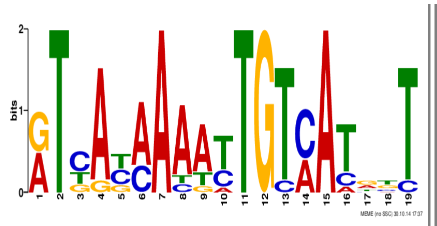 | 4.1e-019 | transporter activity (p=0) **GO:0060003** copper ion export (p=0) | **b1969** (R_score=6.2) **b0995** (R_score=5.9) | **phob** (qval=0.027) |
| **Cluster_49** | b3146, b3147, b3149, b4553 | 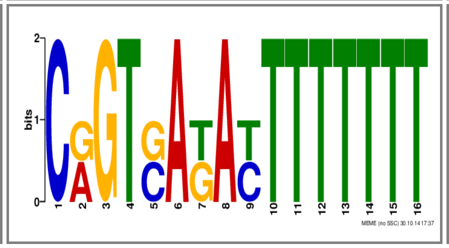 | 1.8e-005 | None | **b3556** (R_score=3.4) **b3075** (R_score=3.1) **b3190** (R_score=3.0) | |
| **Cluster_50** | b1288, b0020, b0019, b1636, b1637, b1657, b3673, b3786, b3784, b3785, b3787, b3788, b3789, b3790, b3791, b3792, b3793, b3794, b0752 | 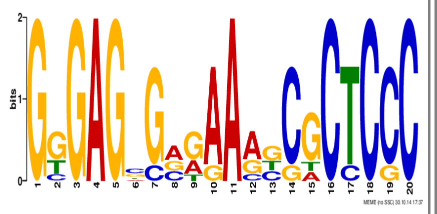 | 9.9e-022 | **GO:0009246** enterobacterial common antigen biosynthetic process (p=0) **GO:0009243** O antigen biosynthetic process (p=8.1E-13) | **b0020** (R_score=5.0) **b1284** (R_score=4.9) **b1658** (R_score=4.7) | |
| **Cluster_51** | b1557, b1558, b0990, b0989, b3008, b0599, b0600 | 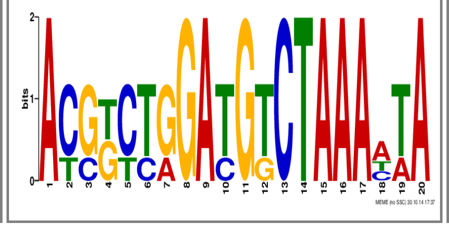 | 1.4e-030 | **GO:0003676** nucleic acid binding (p=1.0E-7) **GO:0009409** response to cold (p=6.5E-7) | **b3938** (R_score=4.3) **b1557** (R_score=4.3) **b3010** (R_score=2.8) | |
| **Cluster_52** | b3915, b2019, b2020, b2021, b2022, b2023, b2024, b2025, b2026, b2415, b2416, b2417, b0173, b2617, b0969 | 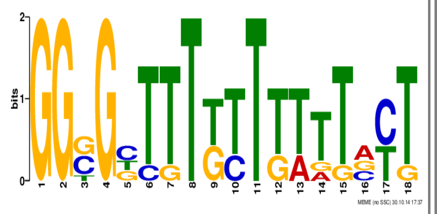 | 2.1e-011 | **GO:0000105** histidine biosynthetic process (p=5.1E-23) **GO:0008652** cellular amino acid biosynthetic process (p=1.5E-11) | **b3912** (R_score=3.4) **b3906** (R_score=3.3) **b3905** (R_score=3.0) | |
| **Cluster_53** | b1914, b1913, b0422, b0420, b0421, b1915, b0423 | 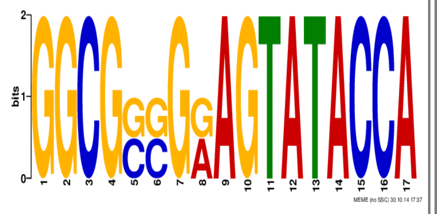 | 7.2e-011 | **GO:0008299** isoprenoid biosynthetic process (p=4.9E-7) **GO:0009228** thiamine biosynthetic process (p=0.0000011) | **b1916** (R_score=5.0) **b1914** (R_score=4.8) **b0676** (R_score=2.8) | |
| **Cluster_54** | b2231, b3461, b0630, b2232 | | 5.1e-006 | None | **b1214** (R_score=3.3) **b0676** (R_score=3.0) **b0683** | |

| | | | | | | |
|---|---|---|---|---|---|---|
| | | 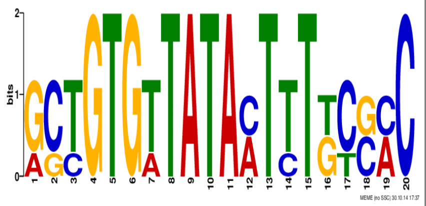 | | | (R_score=2.8) | |
| **Cluster_55** | b0323, b0658, b0659, b0660, b2067 | 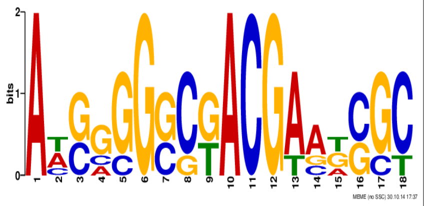 | 6.2e-007 | None | **b0315** (R_score=6.0) **b0535** (R_score=5.5) **b2369** (R_score=5.4) | |
| **Cluster_56** | b2831, b2830, b2829, b0779 | 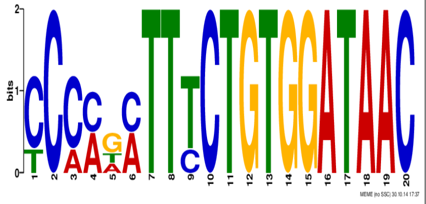 | 1.3e-009 | **GO:0004518** nuclease activity (p=0.000015) **GO:0090305** nucleic acid phosphodiester bond hydrolysis (p=0.000021) | **b3702** (R_score=5.3) **b2865** (R_score=2.6) **b0788** (R_score=2.3) | **dnaa** (qval=0.029) |
| **Cluster_57** | b0821, b0822, b0824, b0823 | 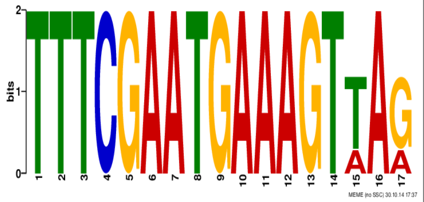 | 4.6e-009 | **GO:0003824** catalytic activity (p=0.00016) | **b0817** (R_score=2.7) **b3190** (R_score=1.9) **b0788** (R_score=1.9) | |
| **Cluster_58** | b3807, b3643, b4558, b3809, b3810, b3811, b3812, b0875, b2216, b2217, b0876, b3644 | 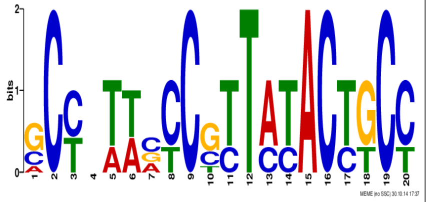 | 2.0e-015 | None | **b2217** (R_score=3.5) **b3702** (R_score=2.9) **b2213** (R_score=2.7) | |
| **Cluster_59** | b0875, b0873, b0872, b1593 | 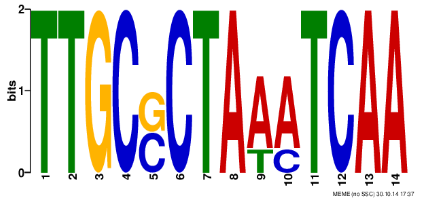 | 2.8e-006 | **GO:0051537** 2 iron, 2 sulfur cluster binding (p=0.0000015) **GO:0051536** iron-sulfur cluster binding (p=0.00015) | **b1334** (R_score=5.9) **b3357** (R_score=4.6) **b1595** (R_score=3.5) | **fnr** (qval=0.029) |
| **Cluster_61** | b1179, b1180, b1857, b1855, b1856, b1858, b1859, b1178, b0296, b4506, b1973 | 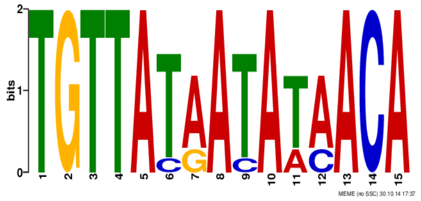 | 3.7e-023 | **GO:0006829** zinc ion transport (p=1.9E-9) **GO:0006811** ion transport (p=0.00013) | **b1187** (R_score=3.2) **b1853** (R_score=2.9) **b1969** (R_score=2.4) | |

| | | | | | | |
|---|---|---|---|---|---|---|
| **Cluster_62** | b4293, b4292, b0755, b1684, b1679, b1680, b1681, b1682, b1683, b1905 | 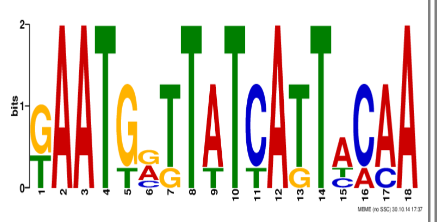 | 9.1e-007 | **GO:0016226** iron-sulfur cluster assembly (p=5.6E-16) **GO:0006979** response to oxidative stress (p=2.3E-8) | **b0683** (R_score=5.3) **b1914** (R_score=4.9) **b4293** (R_score=4.6) | **fur** (qval=0.018) |
| **Cluster_63** | b2344, b1659, b1660, b2343 | 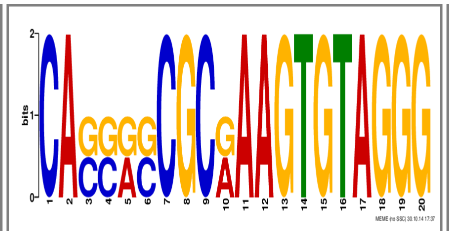 | 1.4e-012 | None | **b1659** (R_score=3.1) **b1658** (R_score=2.3) **b1618** (R_score=2.0) | |
| **Cluster_64** | b3958, b3959, b3960, b3957, b3506, b2818 | 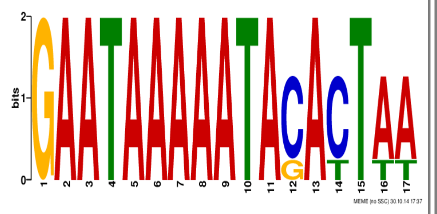 | 2.9e-012 | **GO:0006526** arginine biosynthetic process (p=1.3E-16) **GO:0008652** cellular amino acid biosynthetic process (p=2.4E-10) | **b3237** (R_score=5.0) **b0889** (R_score=3.6) **b4043** (R_score=3.6) | **argr** (qval=0.00095) |
| **Cluster_65** | b3235, b1842, b1841, b1839, b1840, b3920 | 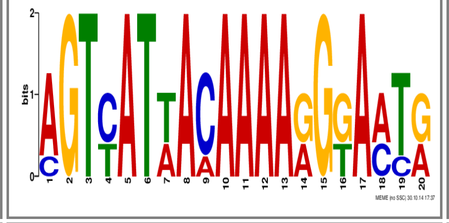 | 2.9e-011 | None | **b3237** (R_score=3.0) **b1853** (R_score=2.9) **b2697** (R_score=2.8) | |
| **Cluster_66** | b3182, b3181, b0413, b0414, b0415, b0416 | 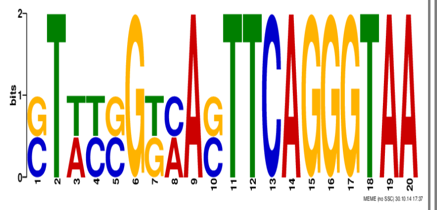 | 1.6e-008 | **GO:0009231** riboflavin biosynthetic process (p=9.5E-8) **GO:0006351** transcription, DNA-dependent (p=0.00058) | **b3190** (R_score=4.8) **b3261** (R_score=3.5) **b0113** (R_score=2.8) | |
| **Cluster_67** | b0381, b0382, b1824, b1823 | 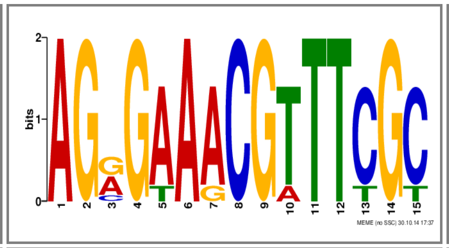 | 1.9e-021 | **GO:0006950** response to stress (p=0.00013) | **b0080** (R_score=5.3) **b0345** (R_score=5.3) **b1658** (R_score=5.2) | |
| | | | | **GO:0008775** acetate CoA-transferase activity (p=3.0E-9) | **b2217** (R_score=7.3) **b2369** | |

| | | | | | | |
|---|---|---|---|---|---|---|
| **Cluster_68** | b2221, b2222, b2223, b2224, b0330, b4340, b0331 | 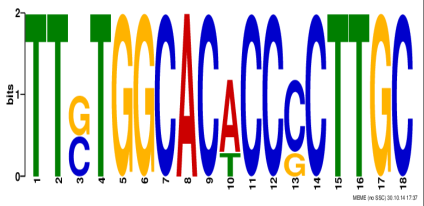 | 1.7e-012 | **GO:0046459** short-chain fatty acid metabolic process (p=3.0E-9) | (R_score=6.5) **b1916** (R_score=5.9) | |
| **Cluster_69** | b1297, b1298, b1299, b0964, b0965, b4339, b4340, b4341 | 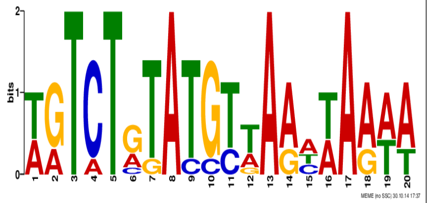 | 1.4e-011 | **GO:0009447** putrescine catabolic process (p=1.2E-9) | **b3405** (R_score=3.4) **b4340** (R_score=2.3) **b1303** (R_score=2.2) | **ompr** (qval=0.064) |
| **Cluster_70** | b2311, b0379, b0380 | 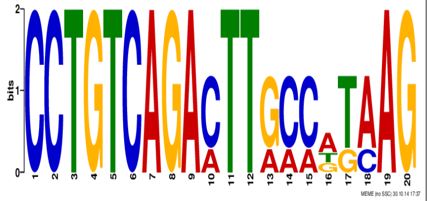 | 1.5e-009 | None | **b3755** (R_score=5.3) **b1439** (R_score=5.0) **b1187** (R_score=4.8) | |
| **Cluster_71** | b1048, b1049, b1659, b1660, b0591, b0590, b0588, b0589, b1047 | 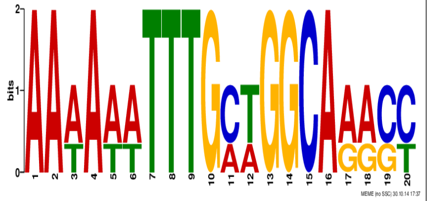 | 2.3e-018 | **GO:0009250** glucan biosynthetic process (p=1.5E-10) **GO:0055072** iron ion homeostasis (p=2.1E-7) | **b1659** (R_score=4.0) **b1658** (R_score=3.5) **b1649** (R_score=2.3) | |
| **Cluster_72** | b1296, b1013, b2361, b2362, b2363 | 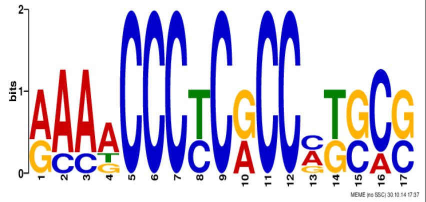 | 7.1e-008 | None | **b1320** (R_score=3.6) **b1014** (R_score=3.2) **b1384** (R_score=2.9) | |
| **Cluster_73** | b4224, b0483, b2797, b2796, b1181 | 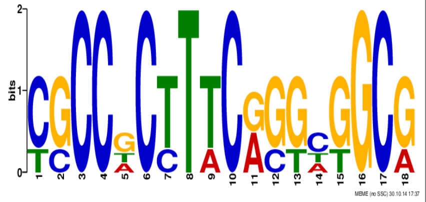 | 1.9e-005 | None | **b3884** (R_score=3.4) **b1770** (R_score=3.2) **b1221** (R_score=3.1) | |
| **Cluster_74** | b3234, b0113, b0482, b0483 | 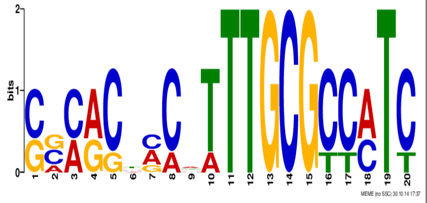 | 4.4e-007 | None | **b0483** (R_score=4.3) **b3202** (R_score=2.3) **b3237** (R_score=2.2) | |
| | | | | | **b2852** | |

| | | | | | | |
|---|---|---|---|---|---|---|
| **Cluster_75** | b0113, b2821, b1281, b1282, b0576, b0575 | 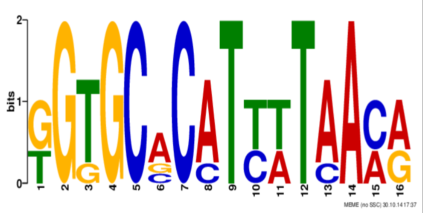 | 3.1e-006 | None | (R_score=6.2)<br>**b2847**<br>(R_score=5.8)<br>**b0995**<br>(R_score=5.3) | |
| **Cluster_77** | b0840, b0841, b1477, b1612, b1611, b1613 | 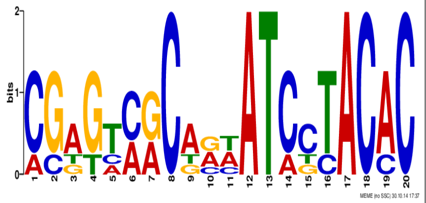 | 4.1e-006 | **GO:0004333**<br>fumarate hydratase activity<br>(p=1.7E-9)<br>**GO:0006099**<br>tricarboxylic acid cycle<br>(p=0.0000038) | **b0840**<br>(R_score=3.7)<br>**b1608**<br>(R_score=3.6)<br>**b1618**<br>(R_score=3.2) | |
| **Cluster_78** | b2709, b0464, b2710, b2711, b3659, b2169, b2167, b2168, b0070, b2170 | 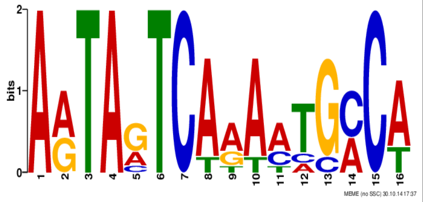 | 1.4e-010 | **GO:0015542**<br>sugar efflux transmembrane transporter activity<br>(p=0)<br>**GO:0015767**<br>lactose transport<br>(p=1.0E-8) | **b2714**<br>(R_score=2.9)<br>**b2707**<br>(R_score=2.8)<br>**b2706**<br>(R_score=2.7) | |
| **Cluster_79** | b0463, b0462, b3264, b3346, b3343, b3344, b3345, b0464 | 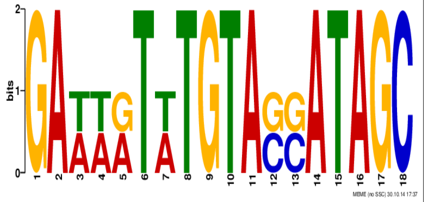 | 1.6e-006 | **GO:0042493**<br>response to drug<br>(p=7.1E-8)<br>**GO:0008033**<br>tRNA processing<br>(p=0.0000012) | **b0464**<br>(R_score=6.0)<br>**b0464**<br>(R_score=6.0)<br>**b3261**<br>(R_score=4.4) | |
| **Cluster_80** | b3498, b3497, b3346, b3343, b3344, b3345, b3499, b2826, b2823, b2825, b1406, b2013, b2012 | 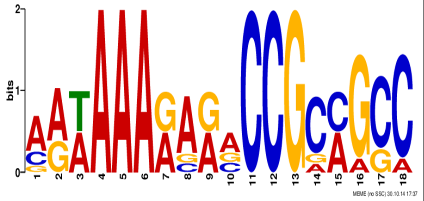 | 1.2e-020 | **GO:0008033**<br>tRNA processing<br>(p=0.000011)<br>**GO:0070475**<br>rRNA base methylation<br>(p=0.000023) | **b3346**<br>(R_score=4.4)<br>**b2015**<br>(R_score=3.5)<br>**b1384**<br>(R_score=2.9) | |
| **Cluster_83** | b1255, b1254, b2398, b2399, b4108 | 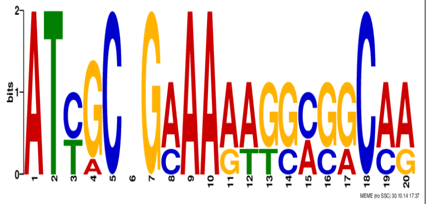 | 3.0e-019 | None | **b2491**<br>(R_score=6.9)<br>**b3261**<br>(R_score=6.1)<br>**b2398**<br>(R_score=6.0) | |
| **Cluster_84** | b4034, b4032, b4033, b4035, b4036, b0835 | 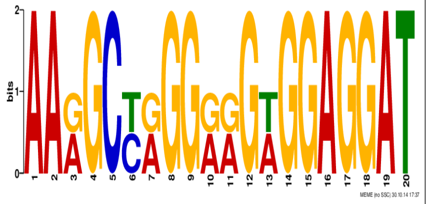 | 1.7e-010 | **GO:0042956**<br>maltodextrin transport<br>(p=0)<br>**GO:0042956**<br>maltodextrin transport<br>(p=0) | **b3418**<br>(R_score=5.7)<br>**b1040**<br>(R_score=5.1)<br>**b4366**<br>(R_score=5.0) | **malt**<br>(qval=0.044) |
| | | | | | **b1916** | |

| | | | | | | |
|---|---|---|---|---|---|---|
| **Cluster_86** | b3161, b0384, b1908 | 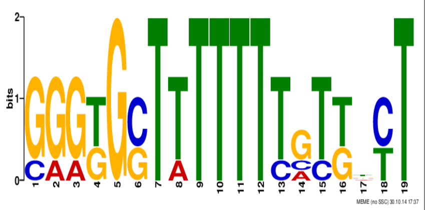 | 5.2e-007 | None | (R_score=2.5)<br>**b1914**<br>(R_score=2.3)<br>**b0378**<br>(R_score=2.2) | |
| **Cluster_87** | b0484, b0123, b0485, b0486 | 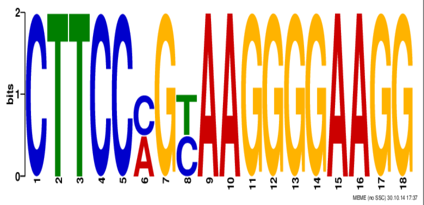 | 5.6e-013 | None | **b1040**<br>(R_score=5.4)<br>**b0535**<br>(R_score=5.2)<br>**b1916**<br>(R_score=4.8) | |
| **Cluster_88** | b4260, b4258, b4259, b0008, b4261, b4262 | 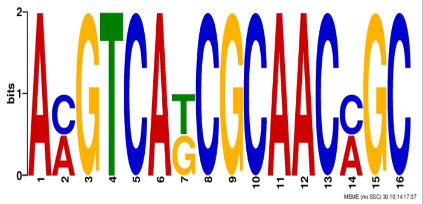 | 2.7e-009 | **GO:0015920**<br>lipopolysaccharide transport<br>(p=9.5E-8)<br>**GO:0043190**<br>ATP-binding cassette (ABC) transporter complex<br>(p=0.000046) | **b0113**<br>(R_score=7.0)<br>**b3755**<br>(R_score=6.5)<br>**b4324**<br>(R_score=5.4) | |
| **Cluster_90** | b1278, b1277, b1431 | 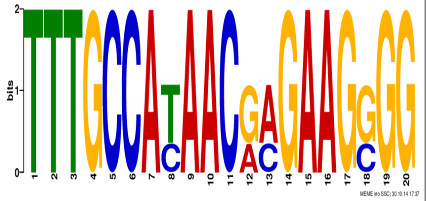 | 1.5e-005 | None | **b1399**<br>(R_score=3.8)<br>**b1438**<br>(R_score=3.6)<br>**b1275**<br>(R_score=3.4) | |
| **Cluster_91** | b0222, b3175, b2412, b0221, b4238, b4237, b2395 | 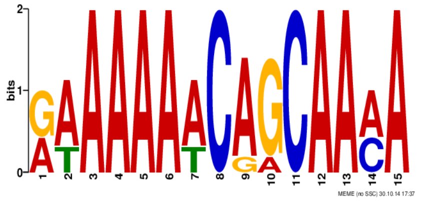 | 1.5e-016 | **GO:0004748**<br>ribonucleoside-diphosphate reductase activity, thioredoxin disulfide as acceptor<br>(p=5.9E-8)<br>**GO:0015949**<br>nucleobase-containing small molecule interconversion<br>(p=0.0000028) | **b4043**<br>(R_score=5.7)<br>**b3190**<br>(R_score=3.2)<br>**b2398**<br>(R_score=2.9) | |
| **Cluster_92** | b2713, b4002, b4003, b4004 | 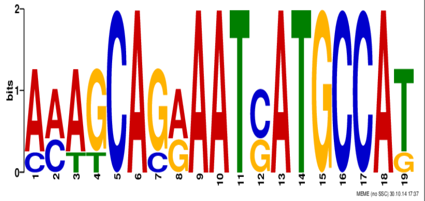 | 2.2e-013 | **GO:0000160**<br>two-component signal transduction system (phosphorelay)<br>(p=0.000026) | **b3202**<br>(R_score=4.6)<br>**b2725**<br>(R_score=3.8)<br>**b4000**<br>(R_score=2.9) | **sig54**<br>(qval=0.065) |
| **Cluster_94** | b3018, b0170, b4414, b0169, b0171, b4065, b2414, b0912, b1694, b1695 | | 1.1e-015 | **GO:0008152**<br>metabolic process | **b1892**<br>(R_score=4.8)<br>**b1696** | |

| | | | | | | |
|---|---|---|---|---|---|---|
| | | 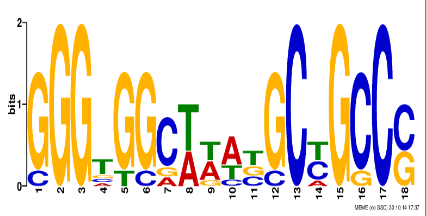 | | (p=0.00087) | (R_score=3.5) **b0900** (R_score=2.8) | |
| **Cluster_95** | b0904, b0903, b3128, b3127, b3124, b3125, b3126, b1429, b1430, b0511, b0512, b0513, b0514, b1428 | 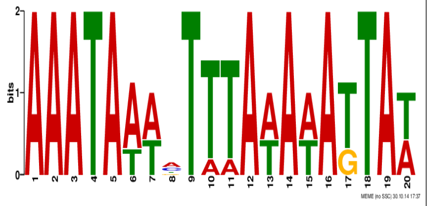 | 4.3e-018 | **GO:0046392** galactarate catabolic process (p=0) **GO:0042838** D-glucarate catabolic process (p=8.2E-11) | **b0900** (R_score=2.7) **b3118** (R_score=2.6) **b3131** (R_score=2.5) | |
| **Cluster_98** | b2153, b3067, b0119, b2154 | 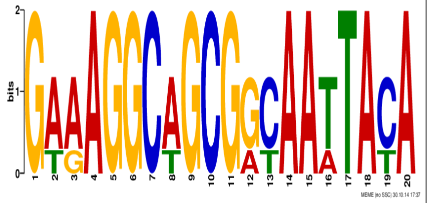 | 5.0e-010 | None | **b2151** (R_score=2.3) **b0146** (R_score=2.3) **b2157** (R_score=2.3) | |
| **Cluster_100** | b0188, b1540, b1853, b0507, b0508, b0509, b2956, b4066 | 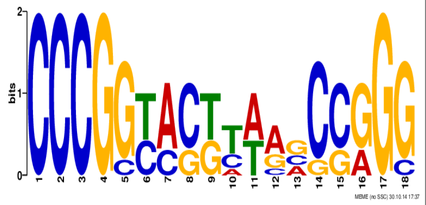 | 3.1e-018 | **GO:0046487** glyoxylate metabolic process (p=4.7E-9) **GO:0009436** glyoxylate catabolic process (p=1.9E-8) | **b1540** (R_score=4.0) **b1853** (R_score=3.6) **b4063** (R_score=2.8) | |
| **Cluster_102** | b2265, b2263, b2264, b2151, b0405, b0406, b0407, b0408, b4374, b4372, b4373 | 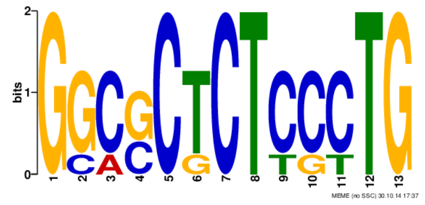 | 3.7e-009 | **GO:0009234** menaquinone biosynthetic process (p=3.4E-9) **GO:0008616** queuosine biosynthetic process (p=7.8E-7) | **b3702** (R_score=2.8) **b0676** (R_score=2.5) **b4377** (R_score=2.5) | |
| **Cluster_104** | b4313, b2151, b0530 | 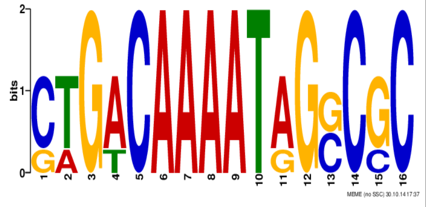 | 8.9e-007 | **GO:0006351** transcription, DNA-dependent (p=0.00054) **GO:0006355** regulation of transcription, DNA-dependent (p=0.00061) | **b2220** (R_score=5.6) **b0330** (R_score=5.5) **b2491** (R_score=5.3) | |
| | | | | **GO:0071978** bacterial-type flagellar swarming motility | **b3842** (R_score=3.9) | |

| | | | | | | |
|---|---|---|---|---|---|---|
| **Cluster_105** | b3842, b3843, b3844, b3606, b4293, b4292, b0684, b0685, b1034, b1033, b1035, b4042, b4041 | 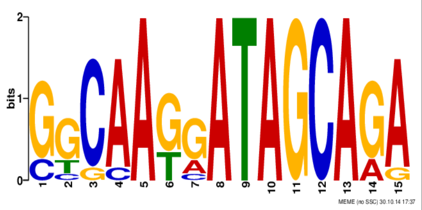 | 1.2e-026 | (p=9.7E-8) **GO:0055072** iron ion homeostasis (p=0.0000011) | **b4293** (R_score=3.6) **b4043** (R_score=3.4) | |
| **Cluster_106** | b1652, b0819, b0820 | 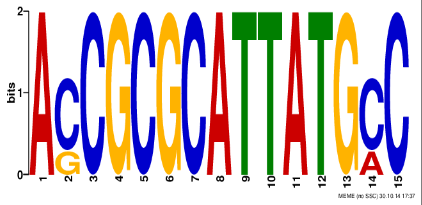 | 6.7e-010 | None | **b0817** (R_score=4.0) **b1658** (R_score=3.9) **b0840** (R_score=3.2) | |
| **Cluster_108** | b0912, b0889, b1560, b1559 | 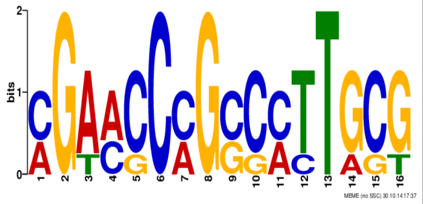 | 4.5e-007 | **GO:0043565** sequence-specific DNA binding (p=0.000023) **GO:0006351** transcription, DNA-dependent (p=0.000044) | **b0889** (R_score=3.7) **b0900** (R_score=3.1) **b1040** (R_score=2.7) | |
| **Cluster_109** | b1566, b0750, b0751, b1253 | 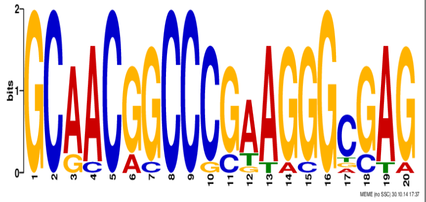 | 3.0e-064 | None | **b0566** (R_score=5.9) **b1790** (R_score=5.7) **b1384** (R_score=5.7) | |
| **Cluster_110** | b1095, b4418, b1088, b1089, b1090, b1091, b1092, b1093, b1094, b0628, b1782, b1783, b1784 | 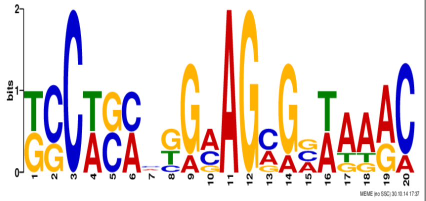 | 2.8e-005 | **GO:0006633** fatty acid biosynthetic process (p=2.1E-14) **GO:0006631** fatty acid metabolic process (p=1.7E-10) | **b0629** (R_score=3.6) **b1214** (R_score=3.0) **b0676** (R_score=2.9) | |
| **Cluster_111** | b2511, b2512, b2513, b2514, b2515, b2516, b1905, b1103, b1104, b1105, b1107, b1108, b1102 | 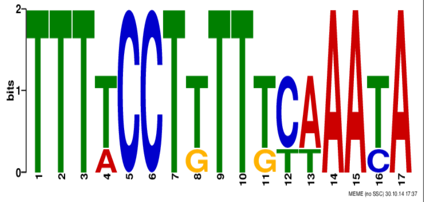 | 1.4e-005 | **GO:0071575** integral to external side of plasma membrane (p=0.0000029) **GO:0008360** regulation of cell shape (p=0.000011) | **b1914** (R_score=3.4) **b2491** (R_score=2.8) **b2479** (R_score=2.8) | |
| **Cluster_112** | b1538, b1778, b1777, b1539, b1779, b1780 | | 5.6e-007 | **GO:0016491** oxidoreductase activity (p=0.00085) | **b0080** (R_score=6.3) **b1658** (R_score=5.2) **b1620** | **cra** (qval=0.075) |

| | | | | | | |
|---|---|---|---|---|---|---|
| | | 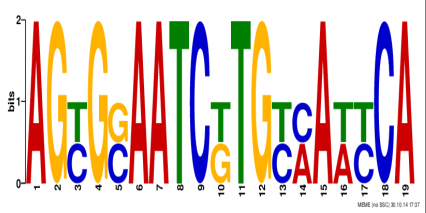 | | | (R_score=5.1) | |
| **Cluster_113** | b2169, b2167, b2168, b1676, b1675 | 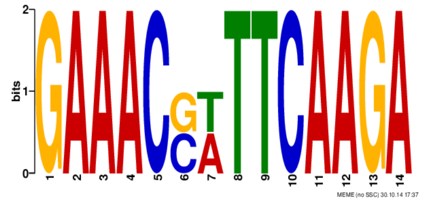 | 1.6e-006 | **GO:0022877** protein-N(PI)-phosphohistidine-fructose phosphotransferase system transporter activity (p=3.0E-8) **GO:0016301** kinase activity (p=9.5E-8) | **b0080** (R_score=6.2) **b1658** (R_score=5.3) **b2151** (R_score=5.1) | **cra** (qval=0.075) |
| **Cluster_114** | b0894, b0895, b0896, b1663, b1662, b1206 | 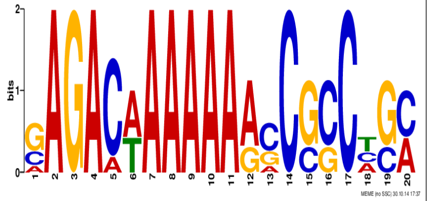 | 7.8e-024 | **GO:0009390** dimethyl sulfoxide reductase complex (p=0) **GO:0009061** anaerobic respiration (p=5.6E-8) | **b1659** (R_score=4.1) **b1658** (R_score=4.0) **b1187** (R_score=3.1) | |
| **Cluster_116** | b1018, b4490, b1019, b1281, b1282, b1520 | 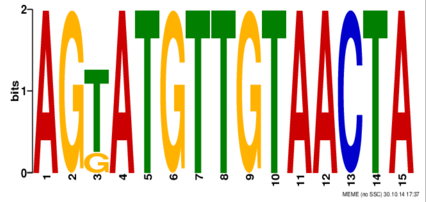 | 5.3e-014 | **GO:0015684** ferrous iron transport (p=3.4E-8) | **b1284** (R_score=4.0) **b1275** (R_score=3.3) **b1658** (R_score=2.7) | |
| **Cluster_117** | b1804, b1742, b1378 | 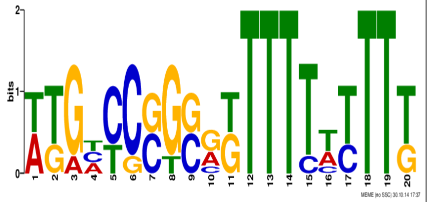 | 1.0e-009 | None | **b1334** (R_score=3.5) **b1659** (R_score=3.2) **b1320** (R_score=3.0) | |
| **Cluster_118** | b1835, b2184, b4218 | 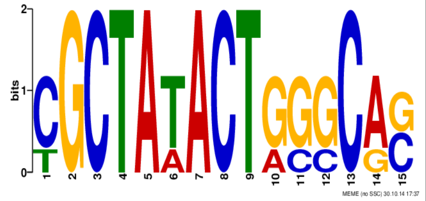 | 1.7e-005 | None | **b1823** (R_score=2.1) **b2193** (R_score=2.0) **b2213** (R_score=2.0) | |
| | | | | **GO:0006164** purine nucleotide | **b1658** (R_score=5.4) | |

| | | | | | | |
|---|---|---|---|---|---|---|
| **Cluster_119** | b2508, b2507, b0411, b2509 | 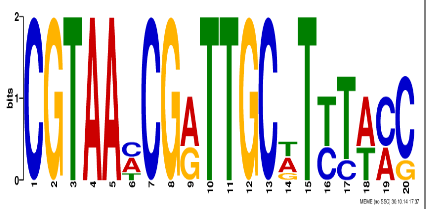 | 2.8e-013 | biosynthetic process (p=2.3E-7) **GO:0005829** cytosol (p=0.00045) | **b0345** (R_score=5.3) **b0080** (R_score=5.1) | **purr** (qval=0.043) |
| **Cluster_123** | b4213, b1759, b1758, b4214, b3553, b4331 | 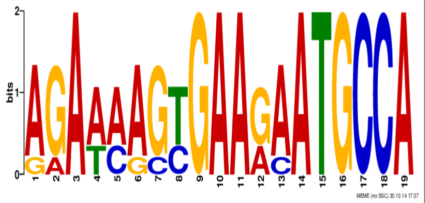 | 1.1e-018 | **GO:0016311** dephosphorylation (p=0.000041) | **b4212** (R_score=3.0) **b3555** (R_score=2.8) **b3556** (R_score=2.6) | |
| **Cluster_124** | b1385, b1384, b3003, b3002 | 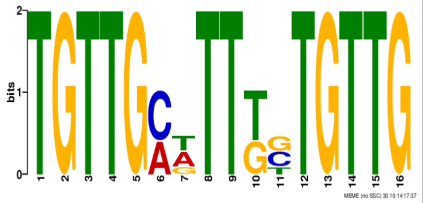 | 1.4e-010 | None | **b1608** (R_score=5.1) **b1969** (R_score=5.0) **b3912** (R_score=5.0) | |
| **Cluster_125** | b4055, b3963, b3964, b3962, b2800, b1781, b4621 | 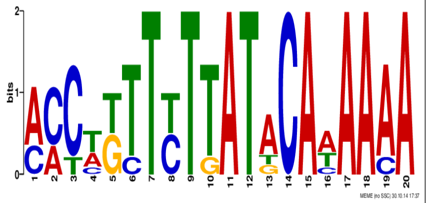 | 1.5e-011 | None | **b3961** (R_score=3.9) **b2805** (R_score=3.4) **b3938** (R_score=3.1) | |
| **Cluster_126** | b3829, b3828, b2942 | 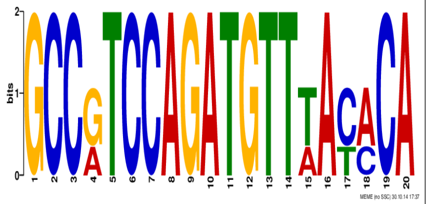 | 3.2e-005 | **GO:0009086** methionine biosynthetic process (p=5.8E-8) **GO:0008652** cellular amino acid biosynthetic process (p=0.000016) | **b3828** (R_score=4.1) **b2929** (R_score=2.2) **b2869** (R_score=2.1) | |
| **Cluster_129** | b3806, b4116, b4115, b3648, b3649, b3650, b3651, b3805, b3802, b3803, b3804, b3647, b3102 | 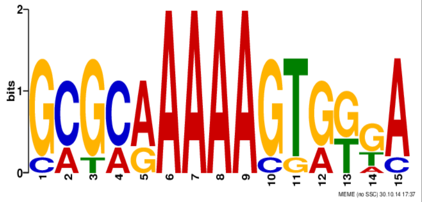 | 4.0e-012 | **GO:0006779** porphyrin-containing compound biosynthetic process (p=5.5E-10) **GO:0033014** tetrapyrrole biosynthetic process (p=2.4E-7) | **b4116** (R_score=3.7) **b3105** (R_score=2.8) **b3755** (R_score=2.7) | |
| **Cluster_130** | b3498, b2218, b3499 | 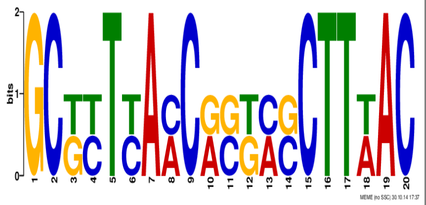 | 9.4e-006 | None | **b3520** (R_score=4.3) **b2217** (R_score=4.2) **b3684** (R_score=3.6) | |

| | | | | | | |
|---|---|---|---|---|---|---|
| **Cluster_131** | b0937, b0933, b0934, b0935, b0936, b0938, b0939, b0940, b0941, b0943, b0944, b2371 | 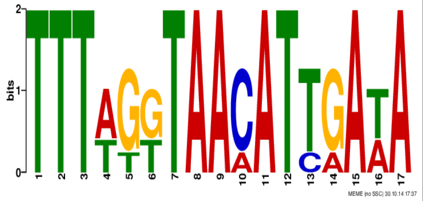 | 1.7e-008 | **GO:0042918** alkanesulfonate transport (p=0) **GO:0043709** cell adhesion involved in single-species biofilm formation (p=5.1E-9) | **b2369** (R_score=3.9) **b0900** (R_score=3.2) **b2364** (R_score=3.0) | |
| **Cluster_133** | b2066, b0064, b3233, b3232 | 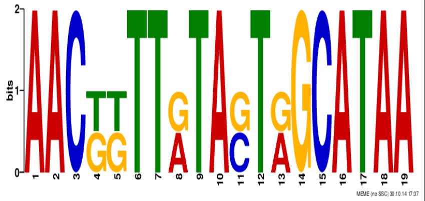 | 1.1e-007 | None | **b3190** (R_score=3.0) **b3237** (R_score=2.9) **b3261** (R_score=2.7) | |
| **Cluster_136** | b0063, b0062, b3418, b0064 | 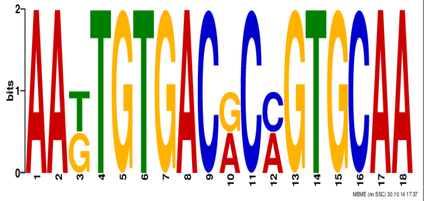 | 2.2e-010 | **GO:0005975** carbohydrate metabolic process (p=0) **GO:0019568** arabinose catabolic process (p=8.2E-14) | **b3357** (R_score=5.5) **b1334** (R_score=5.3) **b0064** (R_score=4.6) | |
| **Cluster_137** | b1047, b1048, b1049, b2393 | 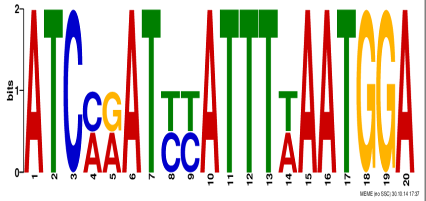 | 4.6e-008 | **GO:0009250** glucan biosynthetic process (p=1.2E-12) **GO:0006970** response to osmotic stress (p=3.3E-7) | **b2398** (R_score=2.6) **b2405** (R_score=2.3) **b1214** (R_score=2.2) | |
| **Cluster_139** | b1799, b2850, b2849, b1800 | 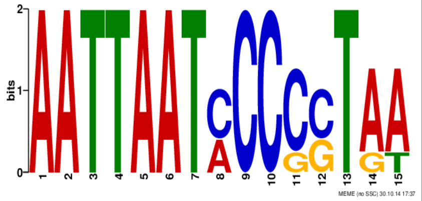 | 6.4e-010 | **GO:0006108** malate metabolic process (p=3.4E-9) | **b3520** (R_score=5.4) **b1914** (R_score=5.2) **b1916** (R_score=5.2) | |
| **Cluster_140** | b3131, b2707, b2702, b2703, b2704, b2705, b2706, b2708, b4046, b3132, b3133, b3134, b3135, b1057, b1056, b4047 | 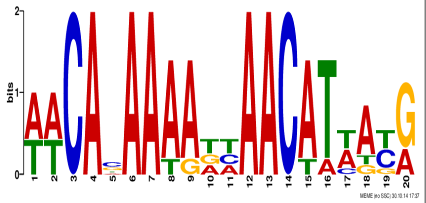 | 6.1e-012 | **GO:0009401** phosphoenolpyruvate-dependent sugar phosphotransferase system (p=4.5E-10) **GO:0008982** protein-N(PI)-phosphohistidine-sugar phosphotransferase activity | **b3912** (R_score=6.3) **b1130** (R_score=5.5) **b4398** (R_score=5.5) | **arca** (qval=0.078) |

| | | | | | | |
|---|---|---|---|---|---|---|
| | | | | (p=0.0000038) | | |
| **Cluster_141** | b2252, b2253, b2254, b2255, b2256, b2257, b4114, b4112, b4113, b0622 | 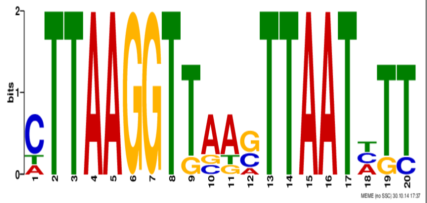 | 9.4e-016 | **GO:0009245** lipid A biosynthetic process (p=4.3E-18) **GO:0010041** response to iron(III) ion (p=1.5E-13) | **b4113** (R_score=2.8) **b2248** (R_score=2.4) **b0603** (R_score=2.3) | |
| **Cluster_142** | b2009, b3565, b3566, b3567, b3568 | 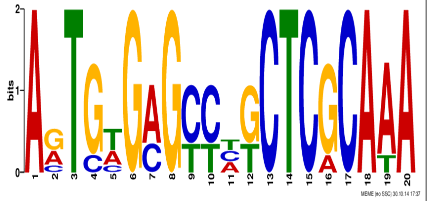 | 9.5e-018 | **GO:0015752** D-ribose transport (p=4.1E-13) **GO:0015753** D-xylose transport (p=4.1E-13) | **b3357** (R_score=4.8) **b1334** (R_score=4.4) **b3555** (R_score=3.7) | **crp_1** (qval=0.070) **crp** (qval=0.070) |
| **Cluster_143** | b1603, b1602, b1684, b1679, b1680, b1681, b1682, b1683, b1604 | 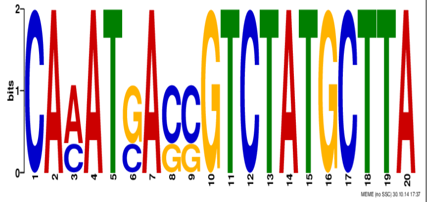 | 3.2e-005 | **GO:0016226** iron-sulfur cluster assembly (p=1.7E-16) **GO:0008746** NAD(P)+ transhydrogenase activity (p=7.1E-9) | **b1642** (R_score=3.3) **b1608** (R_score=3.2) **b1658** (R_score=3.1) | |
| **Cluster_144** | b1952, b0389, b1953 | 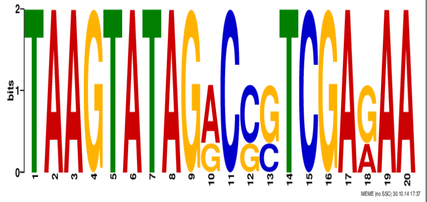 | 5.5e-005 | None | **b0435** (R_score=4.1) **b1739** (R_score=2.9) **b1951** (R_score=2.6) | |
| **Cluster_145** | b4137, b4136, b2322, b2700 | 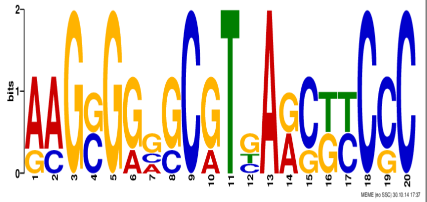 | 1.5e-005 | **GO:0055085** transmembrane transport (p=0.00058) | **b2697** (R_score=4.1) **b2714** (R_score=2.9) **b4135** (R_score=2.8) | |
| **Cluster_147** | b0384, b1536, b1537 | 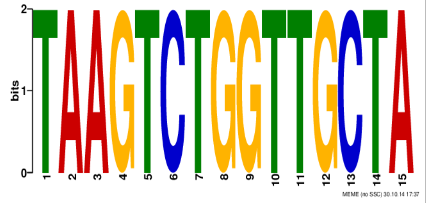 | 6.2e-005 | None | **b1450** (R_score=3.6) **b0435** (R_score=3.3) **b1739** (R_score=3.3) | |
| | | | | **GO:0055072** iron ion homeostasis | **b4293** (R_score=4.3) | |

| | | | | | | |
|---|---|---|---|---|---|---|
| **Cluster_148** | b4293, b4291, b4292, b0987 | 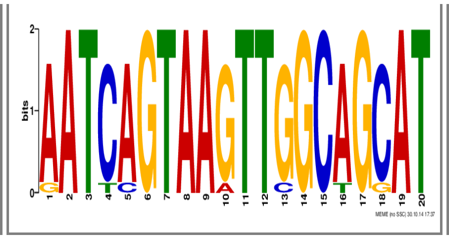 | 3.4e-079 | (p=1.7E-9) **GO:0006811** ion transport (p=4.7E-7) | **b4295** (R_score=2.6) **b4299** (R_score=2.3) | |
| **Cluster_150** | b1557, b3556, b1558, b0990, b0989 | 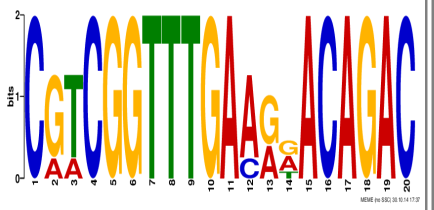 | 9.6e-019 | **GO:0003676** nucleic acid binding (p=1.3E-23) **GO:0006351** transcription, DNA-dependent (p=8.7E-21) | **b1557** (R_score=6.0) **b3556** (R_score=5.4) **b3555** (R_score=2.8) | |
| **Cluster_151** | b1557, b1558, b0990, b0989, b3556 | 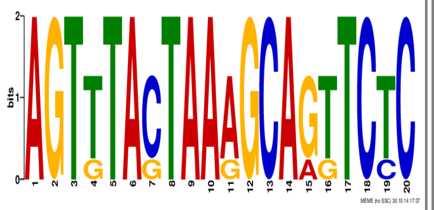 | 1.0e-017 | **GO:0003676** nucleic acid binding (p=1.3E-23) **GO:0006351** transcription, DNA-dependent (p=8.7E-21) | **b1557** (R_score=5.7) **b3556** (R_score=5.0) **b3555** (R_score=2.8) | |
| **Cluster_152** | b3544, b0643, b4061 | 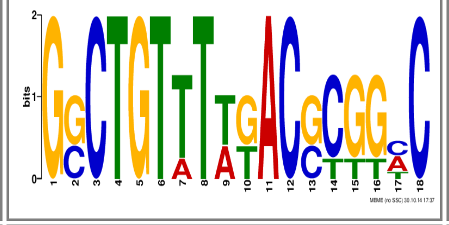 | 1.7e-011 | None | **b3261** (R_score=5.5) **b0330** (R_score=5.1) **b2869** (R_score=5.0) | |
| **Cluster_156** | b3895, b1474, b1475, b1476, b1473 | 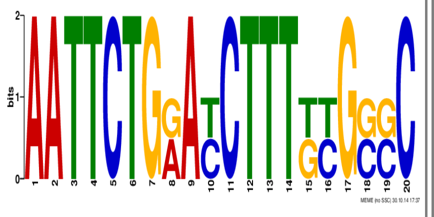 | 3.9e-009 | **GO:0009326** formate dehydrogenase complex (p=9.9E-17) **GO:0008863** formate dehydrogenase (NAD+) activity (p=1.2E-14) | **b3897** (R_score=3.0) **b1499** (R_score=2.2) **b1477** (R_score=2.2) | |
| **Cluster_157** | b0842, b4337, b0841, b0840 | 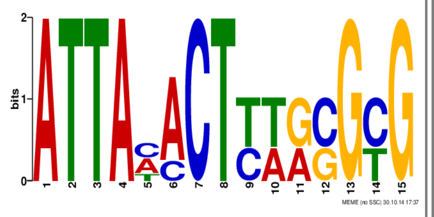 | 2.2e-006 | **GO:0015385** sodium:hydrogen antiporter activity (p=1.4E-9) **GO:0015386** potassium:hydrogen antiporter activity (p=1.4E-9) | **b4396** (R_score=5.2) **b4116** (R_score=5.2) **b3906** (R_score=5.1) | |
| **Cluster_159** | b3665, b3714, b3715 | | 4.5e-009 | **GO:0030145** manganese ion | **b3711** (R_score=2.6) **b3669** | |

| | | | | | | |
|---|---|---|---|---|---|---|
| | | 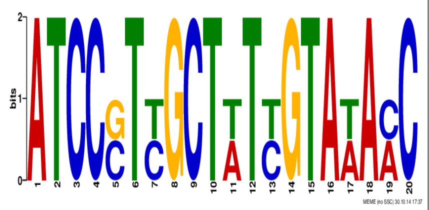 | | binding (p=0.0000021) | (R_score=2.5) **b3702** (R_score=2.3) | |
| **Cluster_165** | b2241, b2242, b2243, b2240, b2239, b2839 | 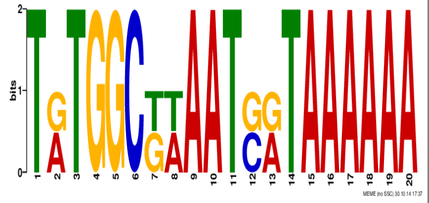 | 2.0e-008 | **GO:0019563** glycerol catabolic process (p=8.6E-11) **GO:0004368** glycerol-3-phosphate dehydrogenase activity (p=1.7E-9) | **b3743** (R_score=4.4) **b0447** (R_score=4.2) **b2217** (R_score=3.7) | |
| **Cluster_166** | b1687, b1686, b1688, b4064 | 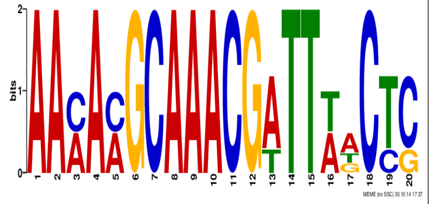 | 9.6e-008 | None | **b1658** (R_score=7.9) **b1620** (R_score=5.4) **b3753** (R_score=5.3) | **purr** (qval=0.0018) |
| **Cluster_167** | b4006, b4459, b4064 | 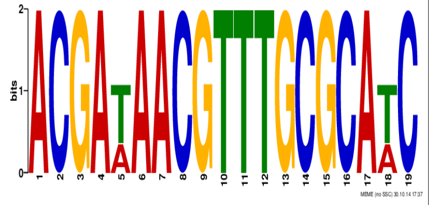 | 6.2e-006 | None | **b3934** (R_score=5.1) **b4241** (R_score=4.8) **b4264** (R_score=4.7) | **purr** (qval=0.00017) |
| **Cluster_169** | b2364, b2365, b2366, b1635 | 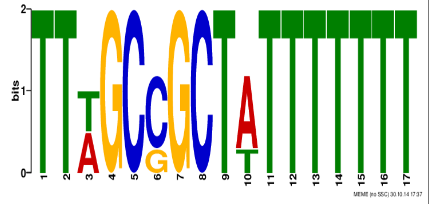 | 6.7e-009 | **GO:0006974** response to DNA damage stimulus (p=0.00073) | **b2364** (R_score=4.1) **b1618** (R_score=2.8) **b1620** (R_score=2.8) | |
| **Cluster_170** | b0386, b0387, b2902 | 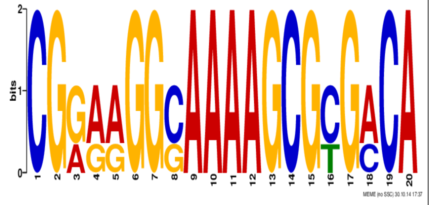 | 5.0e-005 | **GO:0016491** oxidoreductase activity (p=0.00073) | **b0378** (R_score=2.6) **b0399** (R_score=2.4) **b2852** (R_score=2.3) | |
| **Cluster_173** | b0435, b3089, b4026 | 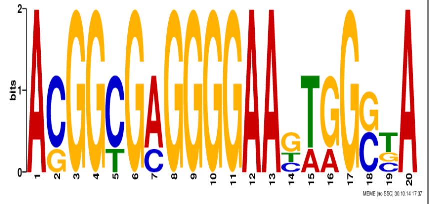 | 1.0e-009 | None | **b0535** (R_score=5.0) **b1040** (R_score=4.9) **b2369** (R_score=4.8) | |
| **Cluster_175** | b0467, b4060, b4061 | | 1.9e-006 | None | **b4018** (R_score=5.5) **b0346** | |

| | | | | | | |
|---|---|---|---|---|---|---|
| | | 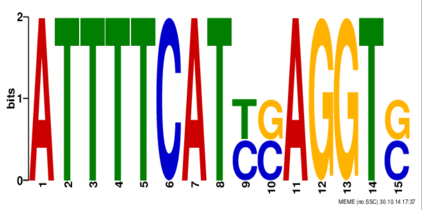 | | | (R_score=5.1)<br>**b0272**<br>(R_score=4.9) | |
| **Cluster_178** | b0260, b3219, b2193, b1330 | 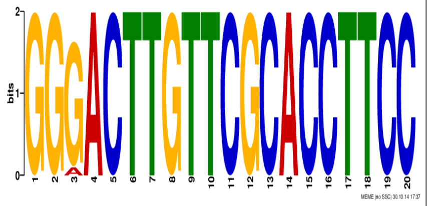 | 3.5e-124 | **GO:0055085**<br>transmembrane transport<br>(p=0.00058) | **b1328**<br>(R_score=3.7)<br>**b2193**<br>(R_score=3.7)<br>**b1334**<br>(R_score=2.9) | |
| **Cluster_182** | b0827, b0826, b3403, b3402 | 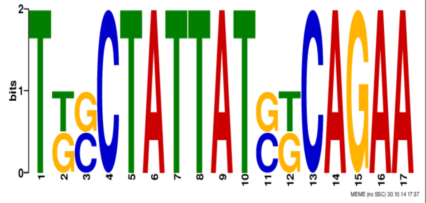 | 3.4e-007 | **GO:0006777**<br>Mo-molybdopterin cofactor biosynthetic process<br>(p=1.2E-7)<br>**GO:0000287**<br>magnesium ion binding<br>(p=0.00020) | **b3405**<br>(R_score=3.8)<br>**b3418**<br>(R_score=2.3)<br>**b3357**<br>(R_score=2.3) | |
| **Cluster_187** | b2316, b2315, b0118, b1777, b1778, b0432, b0428, b0429, b0430, b0431 | 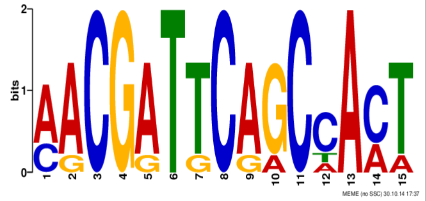 | 5.3e-011 | **GO:0004129**<br>cytochrome-c oxidase activity<br>(p=0)<br>**GO:0008827**<br>cytochrome o ubiquinol oxidase activity<br>(p=0) | **b0080**<br>(R_score=5.6)<br>**b1658**<br>(R_score=5.5)<br>**b1620**<br>(R_score=5.0) | **cra**<br>(qval=0.066) |
| **Cluster_190** | b4519, b1304, b1305, b1306, b1307, b1303 | 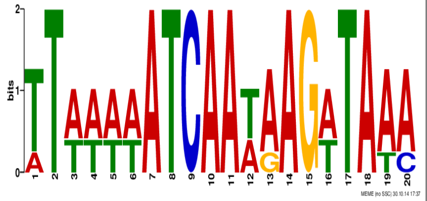 | 1.9e-008 | **GO:0009271**<br>phage shock<br>(p=1.7E-9)<br>**GO:0006950**<br>response to stress<br>(p=2.0E-7) | **b0440**<br>(R_score=4.5)<br>**b1303**<br>(R_score=4.5)<br>**b1306**<br>(R_score=4.3) | **ihfa**<br>(qval=0.053) |
| **Cluster_192** | b0842, b4205, b1804, b0841, b0840 | 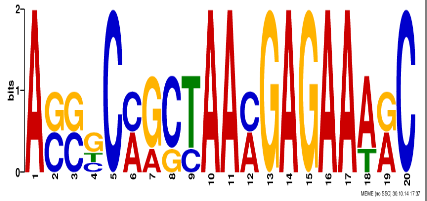 | 2.6e-006 | **GO:0046677**<br>response to antibiotic<br>(p=0.000087)<br>**GO:0005622**<br>intracellular<br>(p=0.00011) | **b0840**<br>(R_score=5.0)<br>**b1770**<br>(R_score=2.6)<br>**b0817**<br>(R_score=2.4) | |
| **Cluster_195** | b1876, b3119, b1875, b1874, b2345 | 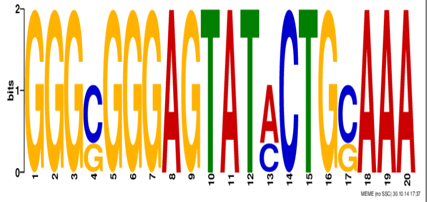 | 8.8e-007 | None | **b1922**<br>(R_score=5.2)<br>**b2573**<br>(R_score=4.8)<br>**b3067**<br>(R_score=4.6) | |

| | | | | | | |
|---|---|---|---|---|---|---|
| **Cluster_197** | b0040, b3118, b2143, b0041, b0042, b0043, b0044 | 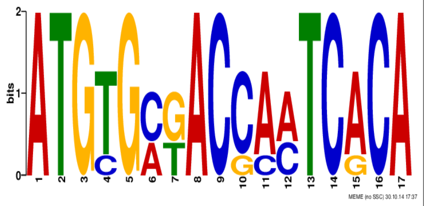 | 5.9e-012 | **GO:0009437** carnitine metabolic process (p=5.2E-13) **GO:0050660** flavin adenine dinucleotide binding (p=0.00011) | **b3357** (R_score=4.7) **b0076** (R_score=4.4) **b1334** (R_score=4.2) | **crp_1** (qval=0.039) **crp** (qval=0.040) |
| **Cluster_200** | b2831, b2830, b2829, b0779, b2234, b2235, b2236, b3863, b3862 | 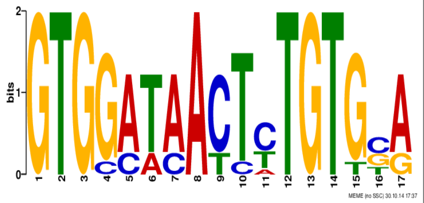 | 6.7e-029 | **GO:0005971** ribonucleoside-diphosphate reductase complex (p=2.8E-8) **GO:0009263** deoxyribonucleotide biosynthetic process (p=1.4E-7) | **b3702** (R_score=6.2) **b0788** (R_score=2.8) **b2248** (R_score=2.6) | **dnaa** (qval=0.037) |
| **Cluster_203** | b2595, b4623, b2594, b2593, b2659, b2660, b2661, b2662, b2663, b2664 | 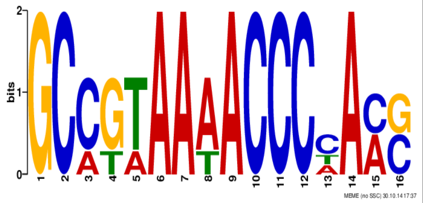 | 6.1e-005 | **GO:0009450** gamma-aminobutyric acid catabolic process (p=1.7E-11) | **b2577** (R_score=5.7) **b2537** (R_score=5.7) **b2808** (R_score=5.3) | |
| **Cluster_204** | b3963, b3964, b2620, b3962, b2619, b2618 | 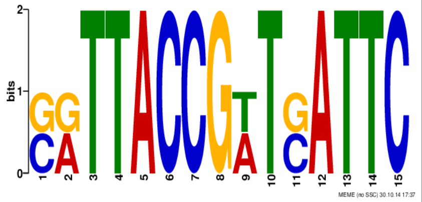 | 5.7e-006 | None | **b3961** (R_score=4.7) **b4000** (R_score=3.4) **b2697** (R_score=3.1) | |
| **Cluster_205** | b1714, b0948, b0949, b0947, b0172, b2277, b2276, b2278, b2279, b2280, b2281, b2282, b2283, b2284, b2285, b2286, b2287, b2288, b2609, b2606, b2607, b2608 | 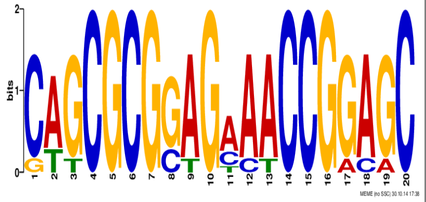 | 2.9e-041 | **GO:0005886** plasma membrane (p=0) **GO:0050136** NADH dehydrogenase (quinone) activity (p=0) | **b1658** (R_score=6.1) **b0080** (R_score=5.8) **b1620** (R_score=5.5) | |
| **Cluster_209** | b2317, b4174, b4169, b4170, b4172, b4173, b4175, b0116, b0114, b0115, b3610, b3607, b3608, b3609 | 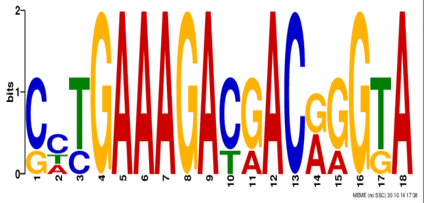 | 1.0e-006 | **GO:0006090** pyruvate metabolic process (p=1.2E-7) **GO:0006096** glycolysis (p=7.0E-7) | **b0113** (R_score=4.3) **b3702** (R_score=3.8) **b4260** (R_score=3.4) | |
| | | | | | **b4212** (R_score=2.0) **b4018** | |

| | | | | | | |
|---|---|---|---|---|---|---|
| **Cluster_211** | b4024, b4216, b4217 | 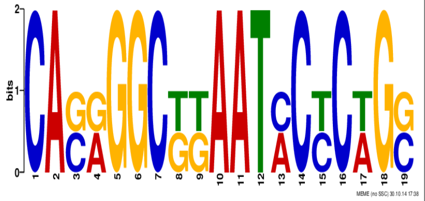 | 1.4e-007 | None | (R_score=2.0)<br>**b4135**<br>(R_score=1.7) | |
| **Cluster_220** | b3618, b3617, b3616, b2833, b3619, b3620, b3621, b3622 | 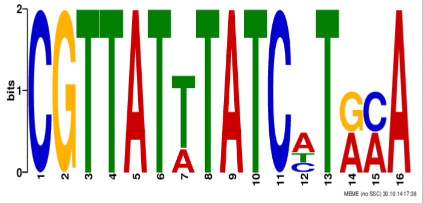 | 2.8e-005 | **GO:0009244** lipopolysaccharide core region biosynthetic process (p=1.8E-10)<br>**GO:0009103** lipopolysaccharide biosynthetic process (p=8.5E-10) | **b3346**<br>(R_score=2.8)<br>**b3702**<br>(R_score=2.7)<br>**b2837**<br>(R_score=2.4) | |
| **Cluster_222** | b3433, b2851, b3434 | 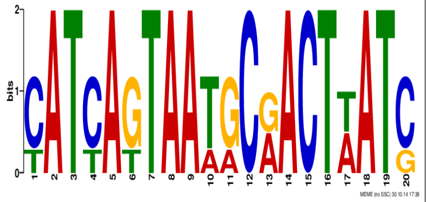 | 3.5e-007 | None | **b2852**<br>(R_score=4.2)<br>**b2847**<br>(R_score=2.9)<br>**b3422**<br>(R_score=2.5) | |
| **Cluster_225** | b3756, b2591, b0201, b3755, b3279 | 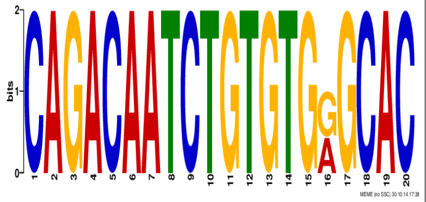 | 5.3e-131 | None | **b3755**<br>(R_score=3.8)<br>**b3261**<br>(R_score=2.7)<br>**b3753**<br>(R_score=2.5) | |
| **Cluster_226** | b3723, b4324, b0076, b3188, b4325, b0392, b1408, b1409, b1410, b1411 | 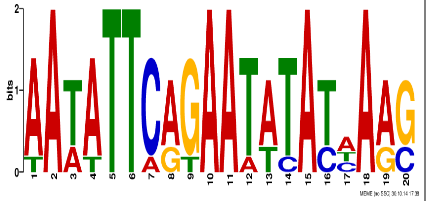 | 2.6e-017 | **GO:0006351** transcription, DNA-dependent (p=0.00064)<br>**GO:0006355** regulation of transcription, DNA-dependent (p=0.00076) | **b0076**<br>(R_score=3.7)<br>**b3190**<br>(R_score=3.1)<br>**b0080**<br>(R_score=2.8) | |
| **Cluster_231** | b2961, b2962, b2960, b2959, b3279 | 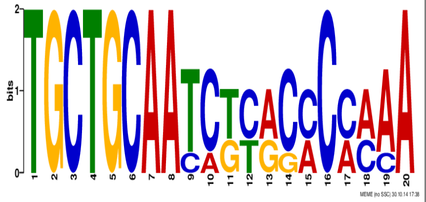 | 6.3e-013 | None | **b1014**<br>(R_score=4.2)<br>**b2929**<br>(R_score=3.7)<br>**b3261**<br>(R_score=3.5) | |
| **Cluster_232** | b1493, b1492, b0208, b0209, b0210, b3517 | | 1.6e-007 | **GO:0051454** intracellular pH elevation (p=6.1E-12)<br>**GO:0019752** | **b3512**<br>(R_score=6.6)<br>**b3520**<br>(R_score=5.4) | **gade**<br>(qval=0.0031)<br>**gadw** |

| | | | | | | |
|---|---|---|---|---|---|---|
| | | 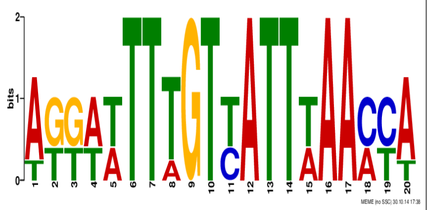 | | carboxylic acid metabolic process (p=1.7E-8) | **b0315** (R_score=4.9) | (qval=0.034) |
| **Cluster_235** | b3766, b0897, b0898 | 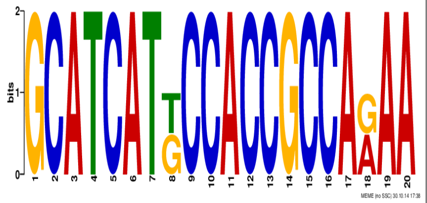 | 1.2e-014 | None | **b0900** (R_score=3.0) **b3753** (R_score=2.6) **b3755** (R_score=2.5) | |
| **Cluster_236** | b1481, b1480, b1482, b0955 | 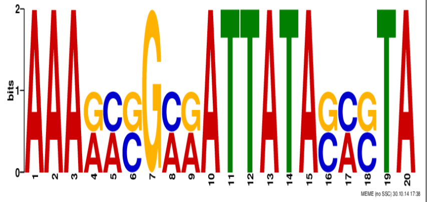 | 9.7e-006 | None | **b2079** (R_score=5.7) **b0694** (R_score=5.5) **b2852** (R_score=5.5) | |
| **Cluster_237** | b0964, b2186, b2738, b2739 | 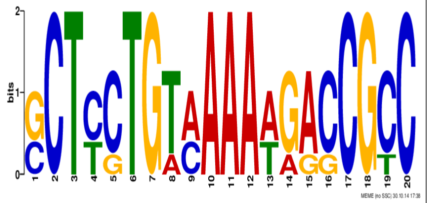 | 1.1e-014 | None | **b0995** (R_score=8.2) **b4133** (R_score=6.3) **b0694** (R_score=6.2) | **phob** (qval=0.096) |
| **Cluster_239** | b4106, b4104, b4105, b1789, b1788 | 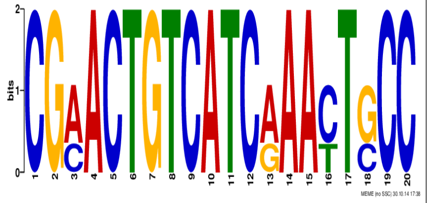 | 1.4e-005 | **GO:0042916** alkylphosphonate transport (p=8.5E-10) **GO:0015716** organic phosphonate transport (p=8.5E-9) | **b0399** (R_score=6.0) **b3912** (R_score=5.9) **b4113** (R_score=5.6) | **phob** (qval=0.0035) |
| **Cluster_240** | b0381, b1739, b0382, b1740 | 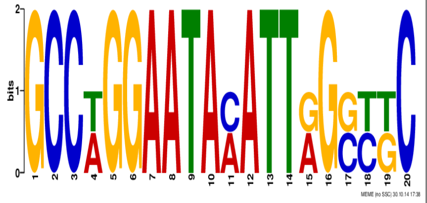 | 9.5e-010 | **GO:0016874** ligase activity (p=0.000037) | **b1739** (R_score=6.0) **b0378** (R_score=3.6) **b1823** (R_score=2.9) | |
| **Cluster_251** | b3816, b2935, b2936 | 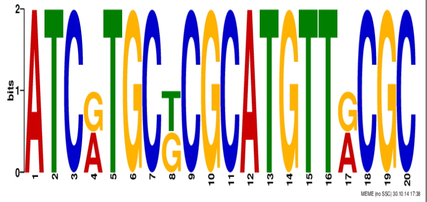 | 8.9e-014 | None | **b2929** (R_score=2.8) **b3261** (R_score=2.3) **b3702** | |

| | | | | | | |
|---|---|---|---|---|---|---|
| | | | | | (R_score=2.3) | |
| **Cluster_252** | b2789, b3555, b4348, b4349, b4350, b2523, b3779, b3780 | 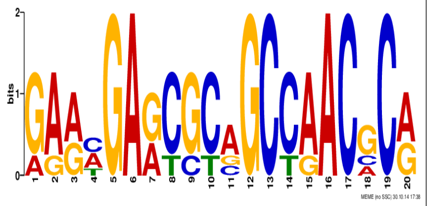 | 1.6e-010 | **GO:0009307** DNA restriction-modification system (p=7.2E-10) **GO:0006304** DNA modification (p=4.7E-9) | **b3884** (R_score=3.1) **b3190** (R_score=3.1) **b3702** (R_score=3.1) | |
| **Cluster_257** | b1605, b4469, b1852 | 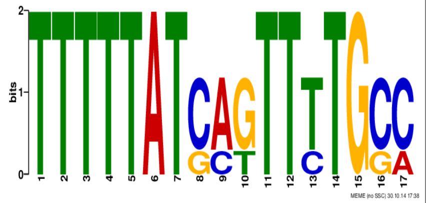 | 4.9e-006 | None | **b1284** (R_score=5.8) **b0840** (R_score=5.7) **b4300** (R_score=5.7) | |
| **Cluster_260** | b3690, b2734, b3689 | 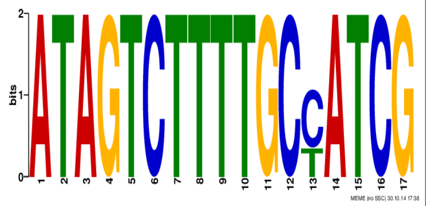 | 2.2e-005 | None | **b3684** (R_score=4.5) **b3680** (R_score=4.0) **b2735** (R_score=3.7) | |
| **Cluster_262** | b0381, b4037, b4252 | 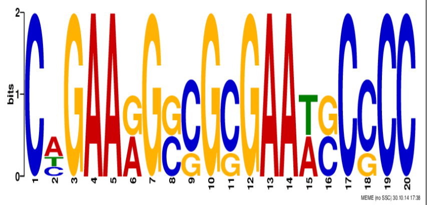 | 1.8e-010 | None | **b1892** (R_score=4.2) **b4251** (R_score=3.4) **b0378** (R_score=3.4) | |
| **Cluster_263** | b1223, b1222, b1221, b0835 | 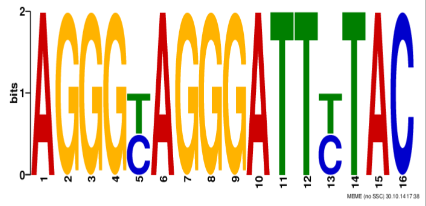 | 1.3e-009 | **GO:0042128** nitrate assimilation (p=4.0E-10) **GO:0000160** two-component signal transduction system (phosphorelay) (p=0.000026) | **b1221** (R_score=4.3) **b1187** (R_score=3.0) **b0817** (R_score=2.8) | |
| **Cluster_264** | b0286, b0283, b0284, b0285, b0865, b0866, b0867 | 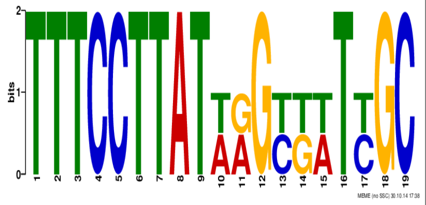 | 2.1e-007 | **GO:0016903** oxidoreductase activity, acting on the aldehyde or oxo group of donors (p=2.9E-12) **GO:0006166** purine ribonucleoside salvage (p=6.0E-10) | **b0435** (R_score=2.7) **b0146** (R_score=2.6) **b1739** (R_score=2.3) | |
| | | | | **GO:0016021** integral to membrane (p=6.5E-23) | **b3261** (R_score=5.2) **b2220** | **fis** |

| | | | | | | |
|---|---|---|---|---|---|---|
| **Cluster_267** | b0622, b2135, b2136 | 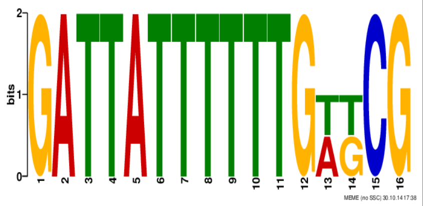 | 1.7e-008 | **GO:0016020** membrane (p=1.6E-22) | (R_score=5.1) **b1201** (R_score=4.8) | (qval=0.0036) |
| **Cluster_268** | b1220, b2671, b2672 | 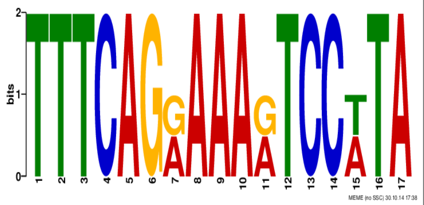 | 9.5e-008 | None | **b1221** (R_score=6.4) **b1040** (R_score=5.6) **b1696** (R_score=5.1) | **arac** (qval=0.076) |
| **Cluster_271** | b1343, b3519, b3003, b3002 | 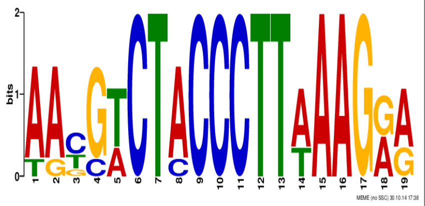 | 1.1e-017 | **GO:0008152** metabolic process (p=0.00036) | **b3520** (R_score=7.6) **b1914** (R_score=5.3) **b1916** (R_score=5.3) | |
| **Cluster_274** | b2233, b2735, b2736, b2737 | 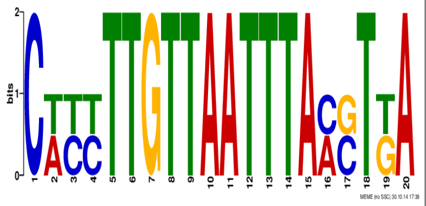 | 3.2e-007 | None | **b2409** (R_score=6.5) **b2364** (R_score=6.1) **b2577** (R_score=5.6) | |
| **Cluster_276** | b2208, b2201, b2202, b2203, b2204, b2205, b2206, b2207, b3010, b3476, b3477, b3478, b3479, b3480, b3011, b2209 | 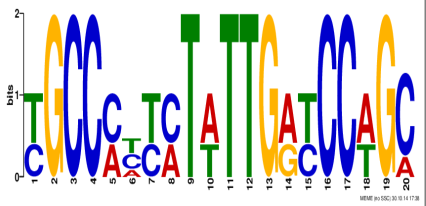 | 1.1e-011 | **GO:0015675** nickel cation transport (p=1.1E-15) **GO:0035444** nickel cation transmembrane transport (p=9.0E-12) | **b3010** (R_score=3.7) **b2213** (R_score=2.2) **b3021** (R_score=1.9) | |
| **Cluster_277** | b2725, b3521, b2726, b2727, b2728, b2729, b2730, b2670 | 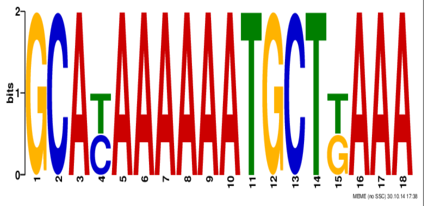 | 7.4e-010 | **GO:0051604** protein maturation (p=3.2E-14) **GO:0016151** nickel cation binding (p=0.0000032) | **b2725** (R_score=4.7) **b3237** (R_score=4.4) **b2669** (R_score=3.0) | |
| **Cluster_279** | b2419, b3520, b3521 | 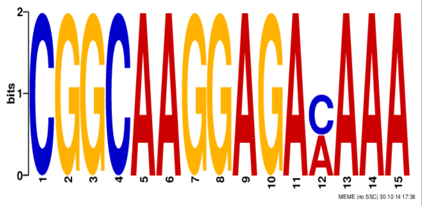 | 8.4e-010 | **GO:0003700** sequence-specific DNA binding transcription factor activity (p=0.00010) **GO:0006351** transcription, DNA-dependent (p=0.00054) | **b3520** (R_score=8.8) **b2369** (R_score=5.1) **b3418** (R_score=5.0) | |

| | | | | | | |
|---|---|---|---|---|---|---|
| **Cluster_284** | b3593, b3482, b0497 | 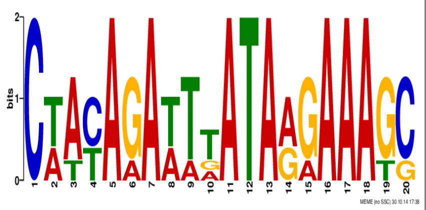 | 5.2e-009 | None | **b3520** (R_score=2.9) **b3585** (R_score=2.8) **b3684** (R_score=2.4) | |
| **Cluster_291** | b1557, b1558, b0990, b0989, b3556, b1552 | 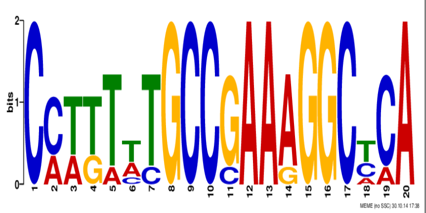 | 7.1e-020 | **GO:0005737** cytoplasm (p=0) **GO:0003676** nucleic acid binding (p=0) | **b1557** (R_score=6.0) **b3556** (R_score=5.0) **b3555** (R_score=3.1) | |
| **Cluster_296** | b0644, b0645, b0646, b1974 | 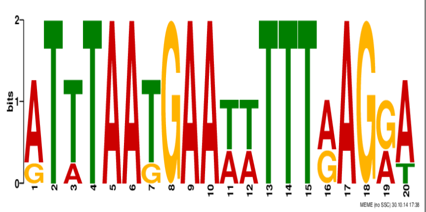 | 4.6e-011 | None | **b3237** (R_score=5.1) **b0535** (R_score=3.4) **b1987** (R_score=3.0) | **argr** (qval=0.00095) |
| **Cluster_299** | b3437, b4476, b4267, b4266, b4268 | 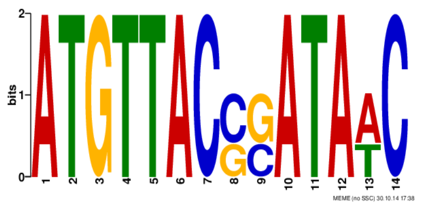 | 4.7e-010 | **GO:0019521** D-gluconate metabolic process (p=2.5E-14) **GO:0046183** L-idonate catabolic process (p=4.1E-13) | **b3438** (R_score=7.2) **b4264** (R_score=6.3) **b3753** (R_score=5.6) | **gntr** (qval=0.000052) |
| **Cluster_301** | b1805, b1387, b1388, b1389, b1390, b1391, b1392, b1393, b1394, b1395 | 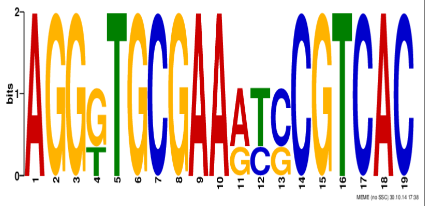 | 1.5e-005 | **GO:0010124** phenylacetate catabolic process (p=0) **GO:0004300** enoyl-CoA hydratase activity (p=1.0E-7) | **b1384** (R_score=4.7) **b1790** (R_score=3.6) **b1799** (R_score=3.1) | |
| **Cluster_302** | b2428, b2427, b2443 | 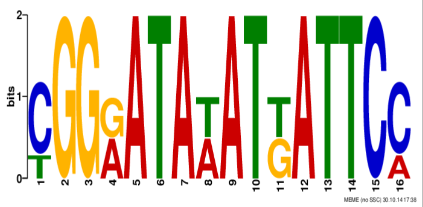 | 3.4e-006 | **GO:0030246** carbohydrate binding (p=3.4E-7) **GO:0005975** carbohydrate metabolic process (p=0.000040) | **b2427** (R_score=3.8) **b2409** (R_score=2.0) **b2399** (R_score=1.6) | |
| **Cluster_306** | b3708, b3709, b1499, b4251, b0442 | 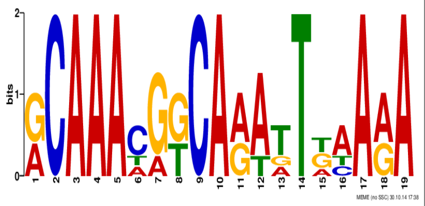 | 1.7e-006 | **GO:0006569** tryptophan catabolic process (p=8.5E-10) **GO:0003677** DNA binding (p=0.00089) | **b1497** (R_score=3.6) **b1499** (R_score=3.5) **b1507** (R_score=2.7) | |

| | | | | | | |
|---|---|---|---|---|---|---|
| **Cluster_307** | b0709, b0710, b0711, b0712, b0713, b4251 | 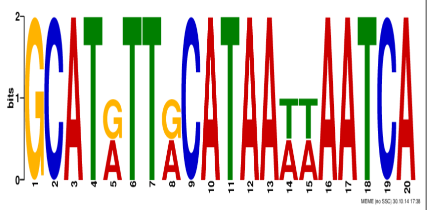 | 2.6e-009 | None | **b3237** (R_score=4.3) **b4264** (R_score=4.2) **b4241** (R_score=4.1) | **argr** (qval=0.0045) |
| **Cluster_308** | b0825, b0076, b0824, b0823 | 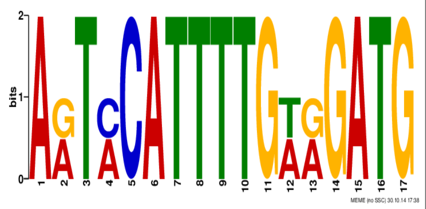 | 1.9e-007 | **GO:0003824** catalytic activity (p=0.00016) | **b0076** (R_score=3.7) **b0900** (R_score=2.7) **b0788** (R_score=2.6) | |
| **Cluster_310** | b0926, b0927, b2301, b2302 | 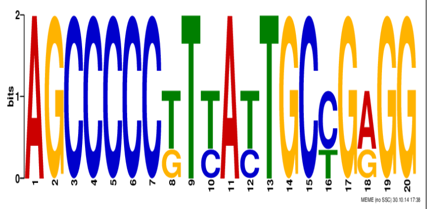 | 8.4e-006 | **GO:0004601** peroxidase activity (p=9.7E-8) | **b1040** (R_score=2.6) **b0889** (R_score=2.2) **b0788** (R_score=2.0) | |
| **Cluster_312** | b0956, b0955, b2902 | 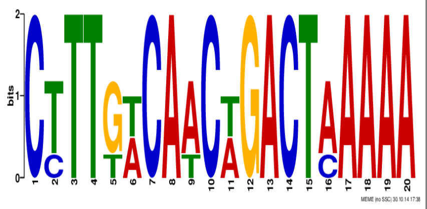 | 9.6e-009 | None | **b0995** (R_score=2.3) **b2852** (R_score=2.3) **b1187** (R_score=2.2) | |
| **Cluster_314** | b1090, b4418, b1088, b1089, b1091, b1092, b3843, b3844, b0288 | 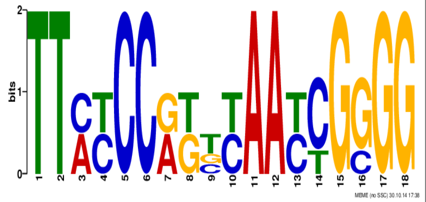 | 5.2e-005 | **GO:0006633** fatty acid biosynthetic process (p=3.9E-8) **GO:0016747** transferase activity, transferring acyl groups other than amino-acyl groups (p=0.0000080) | **b3842** (R_score=4.0) **b3743** (R_score=3.4) **b4135** (R_score=3.0) | |
| **Cluster_317** | b1952, b1627, b1625, b1626, b1628, b1629, b1630, b1631, b1632, b1633, b1953 | 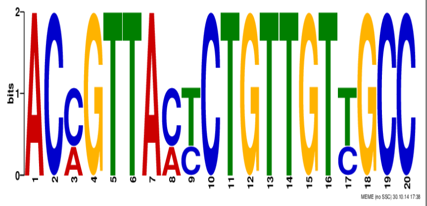 | 2.6e-005 | **GO:0022900** electron transport chain (p=6.1E-14) **GO:0009055** electron carrier activity (p=0.000030) | **b1642** (R_score=3.0) **b1659** (R_score=3.0) **b1658** (R_score=2.9) | |
| **Cluster_318** | b1352, b1351, b4526, b1320, b1353, b1321, b1322 | 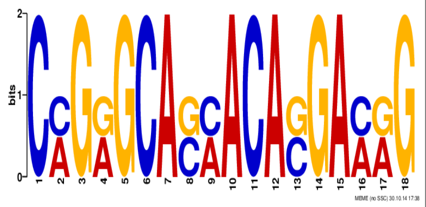 | 5.3e-008 | None | **b1439** (R_score=6.2) **b1450** (R_score=5.6) **b1187** (R_score=5.2) | |

| | | | | | | |
|---|---|---|---|---|---|---|
| **Cluster_321** | b0118, b2214, b0117 | 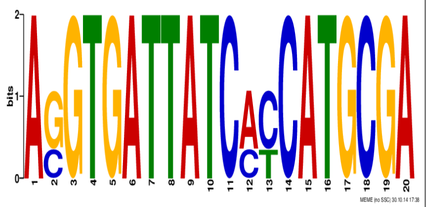 | 5.8e-006 | None | **b2220** (R_score=4.0) **b0267** (R_score=3.1) **b2248** (R_score=3.1) | |
| **Cluster_330** | b1923, b1924, b1925, b1926, b1611, b1612 | 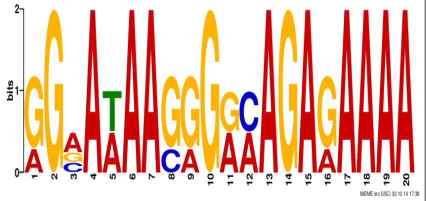 | 2.8e-007 | **GO:0004333** fumarate hydratase activity (p=1.7E-9) **GO:0009288** bacterial-type flagellum (p=1.8E-8) | **b1922** (R_score=5.5) **b1891** (R_score=3.7) **b1892** (R_score=3.3) | |
| **Cluster_333** | b3747, b4238, b4237, b3746, b3745 | 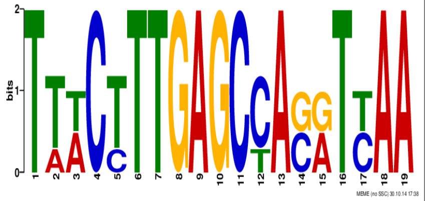 | 7.0e-007 | **GO:0004748** ribonucleoside-diphosphate reductase activity, thioredoxin disulfide as acceptor (p=1.7E-8) **GO:0015949** nucleobase-containing small molecule interconversion (p=8.2E-7) | **b3357** (R_score=4.7) **b1334** (R_score=4.5) **b3912** (R_score=3.8) | |
| **Cluster_337** | b3806, b1288, b3805, b3802, b3803, b3804 | 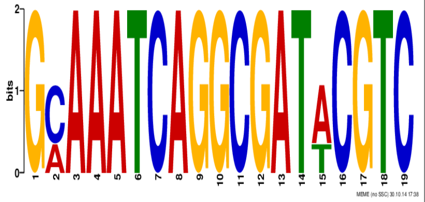 | 5.5e-008 | **GO:0006779** porphyrin-containing compound biosynthetic process (p=2.6E-12) **GO:0033014** tetrapyrrole biosynthetic process (p=1.7E-8) | **b3755** (R_score=4.2) **b1221** (R_score=3.9) **b3912** (R_score=3.9) | |
| **Cluster_342** | b2127, b0131, b0132 | 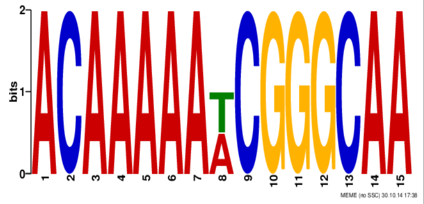 | 5.6e-009 | None | **b1201** (R_score=5.5) **b1303** (R_score=5.5) **b0330** (R_score=5.2) | **glng** (qval=0.056) |
| **Cluster_347** | b1704, b1370, b1372 | 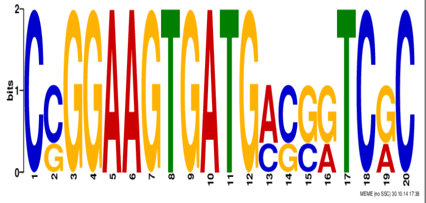 | 2.2e-005 | None | **b1439** (R_score=3.3) **b1696** (R_score=3.3) **b1526** (R_score=3.2) | |
| | | | | **GO:0008746** NAD(P)+ | | |

| | | | | | | |
|---|---|---|---|---|---|---|
| **Cluster_348** | b1603, b1602, b1013, b1604 | 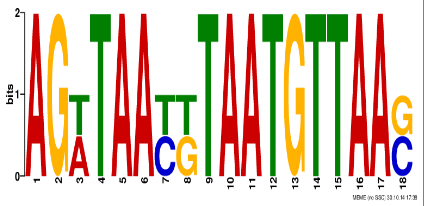 | 5.2e-007 | transhydrogenase activity (p=3.4E-10) **GO:0005887** integral to plasma membrane (p=0.00051) | **b1608** (R_score=3.3) **b1014** (R_score=3.2) **b1658** (R_score=2.9) | **arca** (qval=0.080) |
| **Cluster_351** | b1343, b4431, b1689, b4575 | 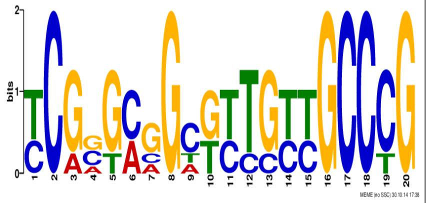 | 1.8e-009 | None | **b1914** (R_score=2.7) **b1570** (R_score=2.4) **b3520** (R_score=2.3) | |
| **Cluster_352** | b2496, b0814, b0813 | 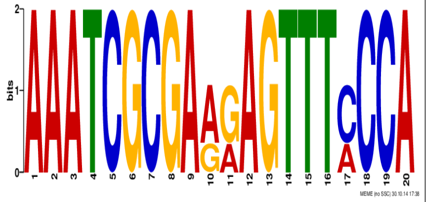 | 1.8e-005 | None | **b0817** (R_score=2.6) **b0840** (R_score=2.6) **b2537** (R_score=2.4) | |
| **Cluster_353** | b1714, b0948, b0949, b0947 | 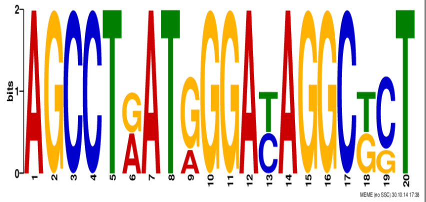 | 5.6e-012 | **GO:0005515** protein binding (p=0.00065) | **b1214** (R_score=3.2) **b1608** (R_score=2.9) **b2697** (R_score=2.7) | |
| **Cluster_357** | b0116, b0114, b0115, b3632, b3623, b3624, b3625, b3626, b3627, b3628, b3629, b3630, b3631, b3633, b3634 | 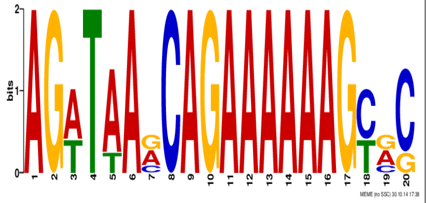 | 2.3e-014 | **GO:0009244** lipopolysaccharide core region biosynthetic process (p=0) **GO:0009103** lipopolysaccharide biosynthetic process (p=3.3E-18) | **b4063** (R_score=4.4) **b0113** (R_score=4.4) **b1162** (R_score=4.3) | |
| **Cluster_358** | b3618, b3872, b3619, b3620, b3621, b3622, b3095, b3096 | 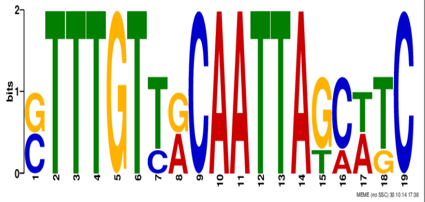 | 4.4e-007 | **GO:0009244** lipopolysaccharide core region biosynthetic process (p=1.8E-10) **GO:0009103** lipopolysaccharide biosynthetic process (p=8.5E-10) | **b3094** (R_score=4.6) **b3190** (R_score=3.5) **b3702** (R_score=3.4) | |
| **Cluster_359** | b0948, b0949, b0097, b0098, b0947 | | 8.0e-008 | **GO:0005737** cytoplasm (p=0.00022) | **b0113** (R_score=3.3) **b0676** (R_score=2.8) **b1214** | |

| | | | | | | |
|---|---|---|---|---|---|---|
| | | 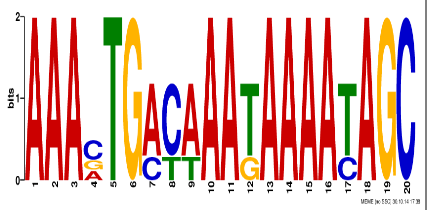 | | | (R_score=2.6) | |
| **Cluster_361** | b1387, b1388, b1389, b1390, b1391, b1392, b1393, b1394, b1395, b0699 | 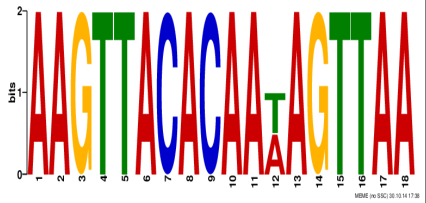 | 2.6e-010 | **GO:0010124** phenylacetate catabolic process (p=0) **GO:0004300** enoyl-CoA hydratase activity (p=1.0E-7) | **b0694** (R_score=6.0) **b0399** (R_score=5.3) **b0995** (R_score=5.1) | |
| **Cluster_367** | b3939, b3938, b2300, b2299, b3493, b3492 | 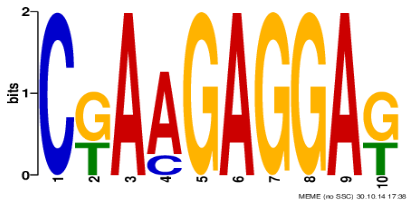 | 1.3e-005 | **GO:0009086** methionine biosynthetic process (p=0.0000011) **GO:0008652** cellular amino acid biosynthetic process (p=0.00031) | **b2369** (R_score=5.8) **b1914** (R_score=5.7) **b3520** (R_score=5.7) | |
| **Cluster_372** | b2468, b2469, b3087 | 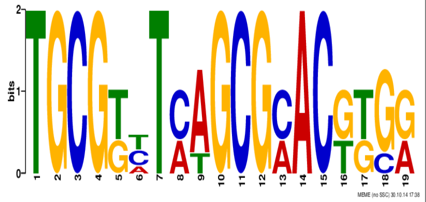 | 3.3e-013 | **GO:0016491** oxidoreductase activity (p=0.00073) | **b2213** (R_score=6.2) **b2382** (R_score=5.7) **b0566** (R_score=5.2) | |
| **Cluster_374** | b3068, b4658, b3001 | 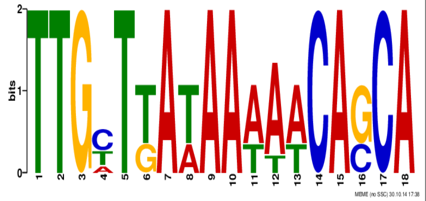 | 4.5e-015 | **GO:0006974** response to DNA damage stimulus (p=0.00019) | **b2869** (R_score=6.1) **b3261** (R_score=5.8) **b2220** (R_score=4.9) | **fis** (qval=0.0026) |
| **Cluster_376** | b0465, b4329, b4328, b4330 | 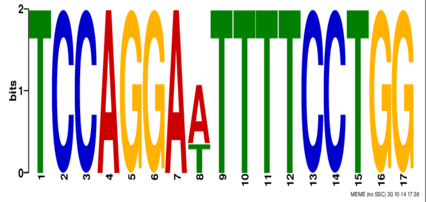 | 2.2e-005 | **GO:0001882** nucleoside binding (p=3.4E-9) | **b0464** (R_score=3.2) **b4327** (R_score=3.2) **b4324** (R_score=2.0) | |
| **Cluster_380** | b0478, b2379, b2380, b2381 | 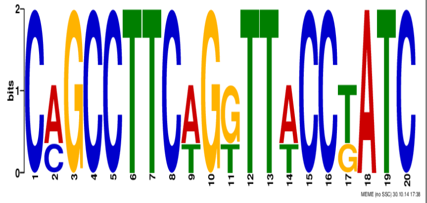 | 8.3e-005 | **GO:0000160** two-component signal transduction system (phosphorelay) (p=0.000026) | **b0483** (R_score=3.0) **b2837** (R_score=2.6) **b2479** (R_score=2.5) | |
| | | | | **GO:0015757** galactose transport | **b1014** | |

| | | | | | | |
|---|---|---|---|---|---|---|
| **Cluster_381** | b1014, b1015, b4227, b4485, b4230, b4231, b4226, b4329, b4328, b4330 | 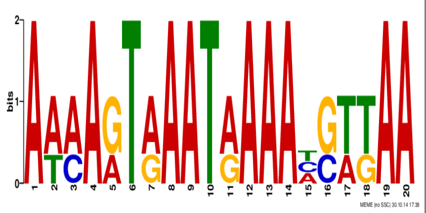 | 9.2e-014 | (p=1.4E-12)<br>**GO:0043190**<br>ATP-binding cassette (ABC) transporter complex<br>(p=9.0E-8) | (R_score=3.6)<br>**b4327**<br>(R_score=2.6)<br>**b4212**<br>(R_score=2.0) | |
| **Cluster_383** | b2965, b0643, b2966 | 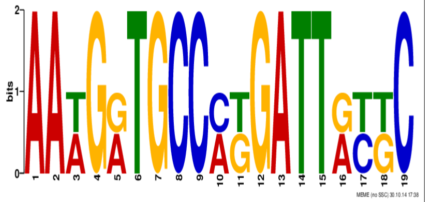 | 3.7e-007 | None | **b0683**<br>(R_score=3.1)<br>**b1916**<br>(R_score=2.4)<br>**b1214**<br>(R_score=2.4) | |
| **Cluster_384** | b3723, b4180, b0122 | 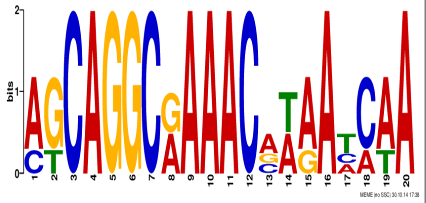 | 4.2e-006 | **GO:0003723**<br>RNA binding<br>(p=0.000031) | **b0113**<br>(R_score=3.4)<br>**b3556**<br>(R_score=2.9)<br>**b3711**<br>(R_score=2.7) | |
| **Cluster_385** | b3599, b1334, b1333, b2343, b3598, b3597 | 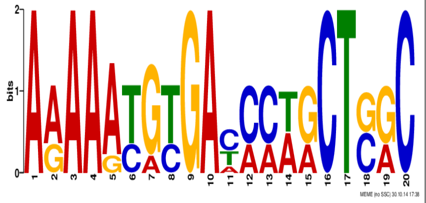 | 1.1e-014 | None | **b3357**<br>(R_score=4.7)<br>**b1334**<br>(R_score=4.5)<br>**b3202**<br>(R_score=2.5) | **crp_1**<br>(qval=0.00067)<br>**crp**<br>(qval=0.00067) |
| **Cluster_388** | b1955, b4353, b4352, b4354 | 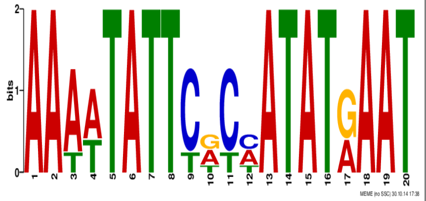 | 1.2e-010 | **GO:0006974**<br>response to DNA damage stimulus<br>(p=0.000011) | **b1951**<br>(R_score=2.7)<br>**b4357**<br>(R_score=2.5)<br>**b1987**<br>(R_score=2.2) | |
| **Cluster_391** | b3093, b3092, b3091, b0644, b0645, b0646 | 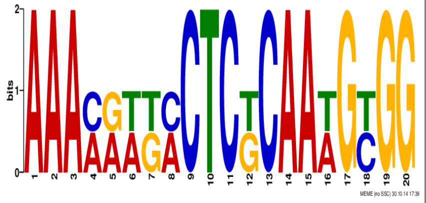 | 4.5e-007 | **GO:0019698**<br>D-galacturonate catabolic process<br>(p=3.4E-8) | **b3094**<br>(R_score=2.6)<br>**b3190**<br>(R_score=2.5)<br>**b0676**<br>(R_score=2.4) | |
| **Cluster_392** | b3756, b2591, b0201, b3755, b3279 | 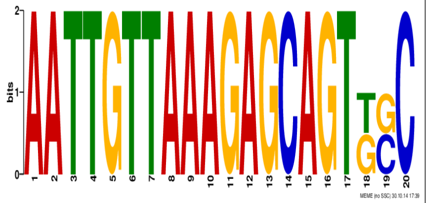 | 3.5e-062 | None | **b1530**<br>(R_score=4.4)<br>**b0676**<br>(R_score=4.2)<br>**b1642**<br>(R_score=4.1) | |
| | | | | | **b3357**<br>(R_score=5.4) | **crp_1** |

| | | | | | | |
|---|---|---|---|---|---|---|
| **Cluster_393** | b3519, b3574, b3518, b3575, b3576 | 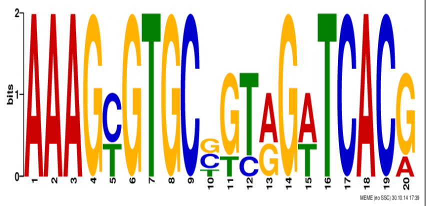 | 1.9e-016 | None | **b3520** (R_score=5.1) **b1334** (R_score=4.6) | (qval=0.0024) **crp** (qval=0.0024) |
| **Cluster_394** | b3225, b3221, b3222, b3223, b3224, b4310, b4277 | 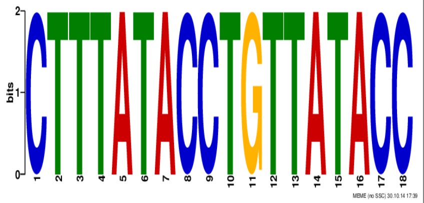 | 1.9e-012 | **GO:0019262** N-acetylneuraminate catabolic process (p=1.4E-11) **GO:0005975** carbohydrate metabolic process (p=8.8E-7) | **b4340** (R_score=5.6) **b4324** (R_score=5.5) **b4357** (R_score=5.2) | |
| **Cluster_395** | b3128, b3127, b3124, b3125, b3126, b3211 | 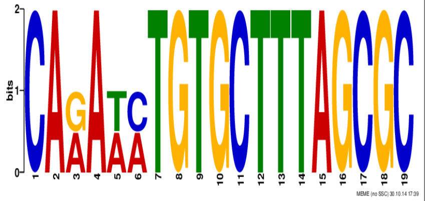 | 1.2e-011 | **GO:0046392** galactarate catabolic process (p=0) **GO:0042838** D-glucarate catabolic process (p=1.2E-12) | **b3357** (R_score=4.9) **b1334** (R_score=4.1) **b3118** (R_score=3.4) | **crp_1** (qval=0.069) **crp** (qval=0.069) |
| **Cluster_399** | b1817, b1818, b1819, b0876, b1816 | 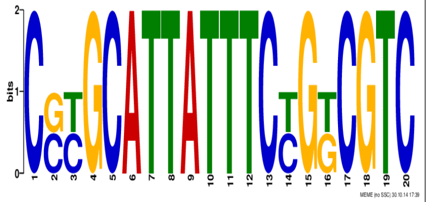 | 3.8e-012 | **GO:0015761** mannose transport (p=0) **GO:0022870** protein-N(PI)-phosphohistidine-mannose phosphotransferase system transporter activity (p=0) | **b1799** (R_score=2.6) **b1827** (R_score=2.5) **b1823** (R_score=2.3) | |
| **Cluster_402** | b2392, b2393, b0129 | 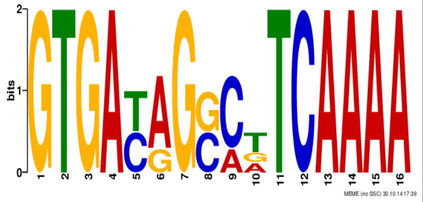 | 6.3e-006 | **GO:0006810** transport (p=2.2E-23) **GO:0016021** integral to membrane (p=6.5E-23) | **b2398** (R_score=2.7) **b2217** (R_score=2.3) **b0113** (R_score=2.1) | |
| **Cluster_408** | b2709, b2710, b2711, b3421, b1387, b3422, b1472, b1388, b1389, b1390, b1391, b1392, b1393, b1394, b1395 | 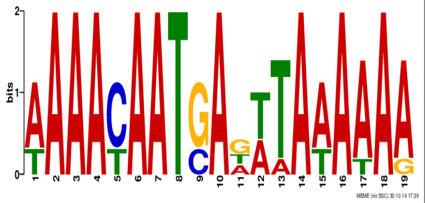 | 8.1e-019 | **GO:0010124** phenylacetate catabolic process (p=7.4E-24) **GO:0004300** enoyl-CoA hydratase activity (p=3.8E-7) | **b4000** (R_score=5.1) **b0440** (R_score=4.8) **b3422** (R_score=4.2) | **arca** (qval=0.098) |
| | | | | | **b4327** | |

| | | | | | | |
|---|---|---|---|---|---|---|
| **Cluster_409** | b4453, b4454, b4325 | 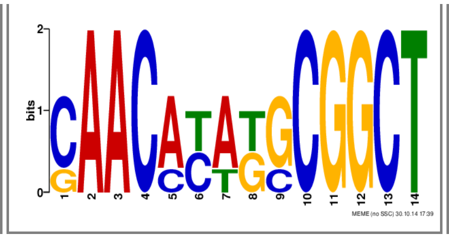 | 1.3e-005 | None | (R_score=2.8) **b4324** (R_score=2.8) **b4300** (R_score=2.7) | |
| **Cluster_410** | b3265, b4329, b4328, b4330 | 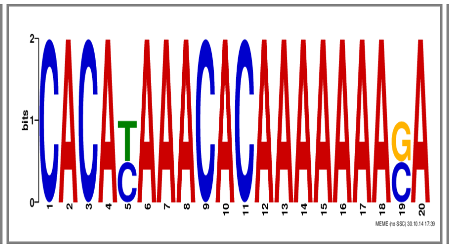 | 1.2e-011 | **GO:0001882** nucleoside binding (p=3.4E-9) | **b4327** (R_score=3.3) **b3264** (R_score=2.8) **b3261** (R_score=2.2) | |
| **Cluster_411** | b3806, b3805, b3803, b3804, b4249 | 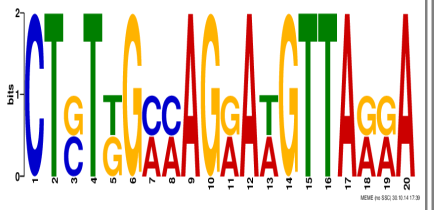 | 1.2e-006 | **GO:0006779** porphyrin-containing compound biosynthetic process (p=7.4E-10) **GO:0033014** tetrapyrrole biosynthetic process (p=8.5E-9) | **b3773** (R_score=3.9) **b4324** (R_score=3.9) **b3868** (R_score=3.8) | |
| **Cluster_413** | b0032, b0033, b1012, b1006, b1008, b1009, b1010, b1011, b1013 | 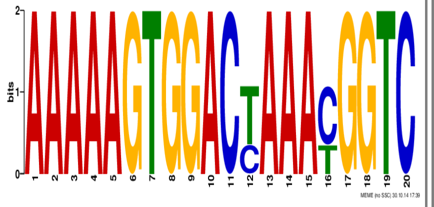 | 5.5e-006 | **GO:0006212** uracil catabolic process (p=7.0E-20) **GO:0019740** nitrogen utilization (p=8.4E-17) | **b1013** (R_score=5.6) **b1111** (R_score=5.1) **b0846** (R_score=5.0) | **rutr** (qval=3.1E-7) |
| **Cluster_415** | b2241, b2242, b2243, b2240, b2239, b2111, b2109, b2110 | 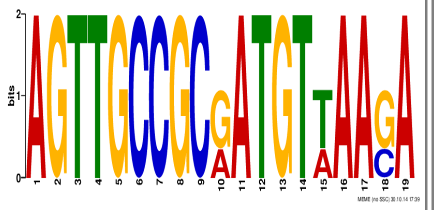 | 2.0e-005 | **GO:0019563** glycerol catabolic process (p=4.0E-10) **GO:0052591** sn-glycerol-3-phosphate:ubiquinone-8 oxidoreductase activity (p=4.7E-9) | **b2248** (R_score=2.5) **b2220** (R_score=2.5) **b2151** (R_score=2.0) | |
