# Supplementary material for: An Integrated Approach to Reconstructing Genome-Scale Transcriptional Regulatory Networks
Source: PLoS Comput Biol. 2015 Feb 27;11(2):e1004103. doi: 10.1371/journal.pcbi.1004103 (PMC4344238; doi:10.1371/journal.pcbi.1004103)
Supplement: S4 Fig — The phylogenetic tree was constructed by the neighbor-joining method using aligned gyrB DNA sequences from 52 representative species of the major genera of α-proteobacteria. Sequence alignment was done using Clustal X and phylogenetic tree generated using phylip. Escherichia coli was used as the out group in this analysis. The grey bar next to each organism is a measure of the number of orthologs that organism shares with R. sphaeroides 2.4.1. The dash red circle corresponds to the number of orthologs E. coli shares with R. sphaeroides 2.4.1. Organisms selected for phylogenetic footprinting are identified with a red star. Phylogenetic tree was visualized using iTOL. (PDF) [file pcbi.1004103.s004.pdf]

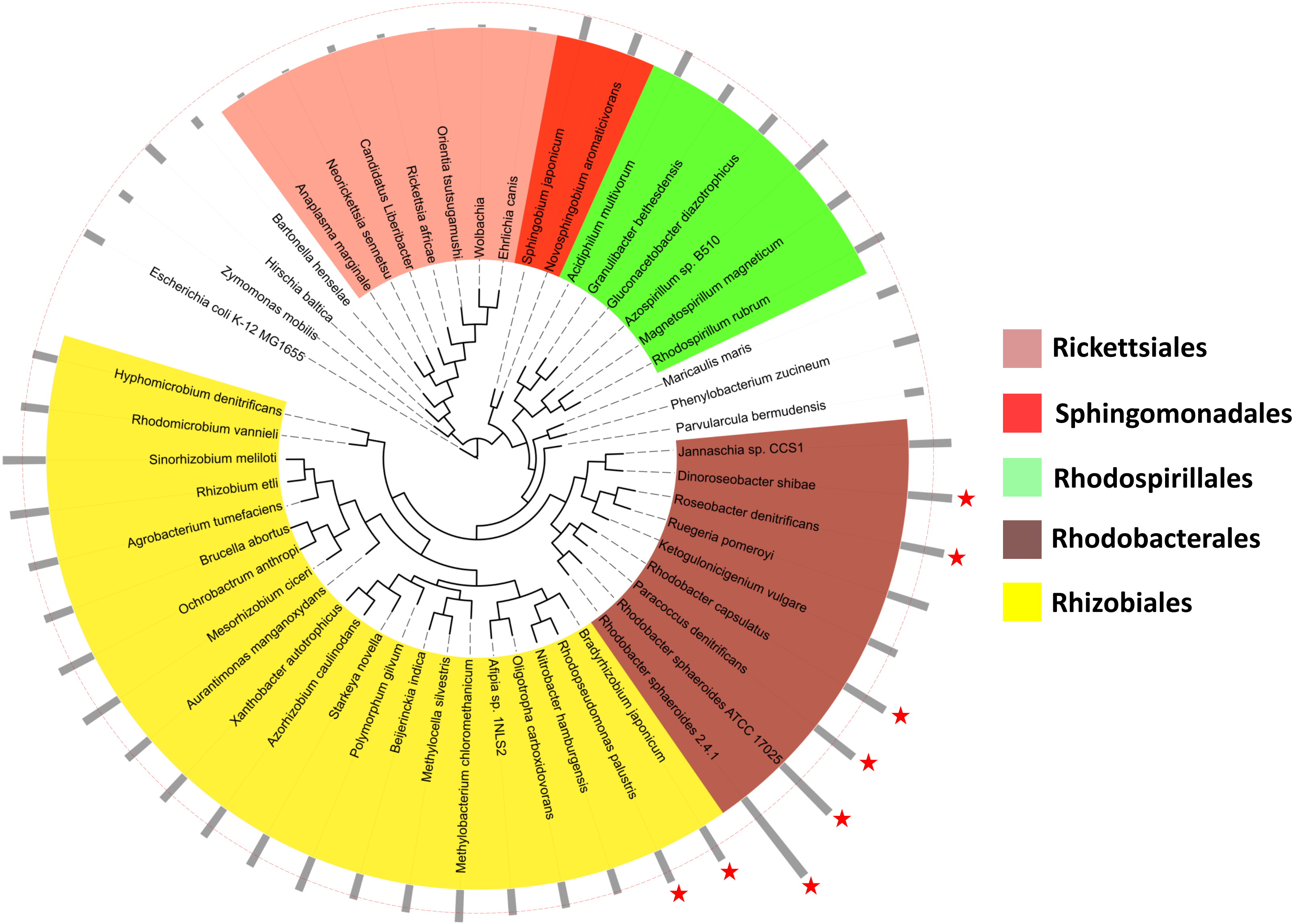

Rickettsiales
Sphingomonadales
Rhodospirillales
Rhodobacterales
Rhizobiales
Escherichia coli K-12 MG1655
Zymomonas mobilis
Hirschia baltica
Bartonella henselae
Anaplasma marginale
Neorickettsia sennetsu
Candidatus Liberibacter
Rickettsia africae
Orientia tsutsugamushi
Wolbachia
Ehrlichia canis
Sphingobium japonicum
Novosphingobium aromaticivorans
Acidiphilium multivorum
Granulibacter bethesdensis
Gluconacetobacter diazotrophicus
Azospirillum sp. B510
Magnetospirillum magneticum
Rhodospirillum rubrum
Maricaulis maris
Phenylobacterium zucineum
Parvularcula bermudensis
Jannaschia sp. CCS1
Dinoroseobacter shibae
Roseobacter denitrificans
Ruegeria pomeroyi
Ketogulonicigenium vulgare
Rhodobacter capsulatus
Paracoccus denitrificans
Rhodobacter sphaeroides ATCC 17025
Rhodobacter sphaeroides 2.4.1
Bradyrhizobium japonicum
Rhodopseudomonas palustris
Nitrobacter hamburgensis
Oligotropha carboxidovorans
Afipia sp. 1NLS2
Methylobacterium chloromethanicum
Methylocella silvestris
Beijerinckia indica
Polymorphum gilvum
Starkeya novella
Azorhizobium caulinodans
Xanthobacter autotrophicus
Aurantimonas manganoxydans
Mesorhizobium ciceri
Ochrobactrum anthropi
Brucella abortus
Agrobacterium tumefaciens
Rhizobium etli
Sinorhizobium meliloti
Rhodomicrobium vannieli
Hyphomicrobium denitrificans
