# Supplementary material for: An Integrated Approach to Reconstructing Genome-Scale Transcriptional Regulatory Networks
Source: PLoS Comput Biol. 2015 Feb 27;11(2):e1004103. doi: 10.1371/journal.pcbi.1004103 (PMC4344238; doi:10.1371/journal.pcbi.1004103)
Supplement: S1 Dataset — This dataset consists of 3 folders containing data used for the analysis presented in the main text and a summary of the results from the E. coli analysis. These folder include: “Ecoli _results”, which contains a summary of the results obtained from the E. coli analysis; “Integrate_v1.0”, which contains sample code for running the presented analysis (the most up-to-date version of this code can be obtained from the figshare link provided in the Materials and methods); and “Rsp_data”, which contains the R. sphaeroides gene expression compendium used in our analysis. (ZIP) [file pcbi.1004103.s024.zip › S1 Dataset/Integrate_v1.0/bin/DISTILLER-V2/readme.pdf]

DISTILLER Version 1

DISTILLER is free to use for academic purposes only. If used in your academic work, please include all relevant references by its authors. For any non-academic purpose, please contact its authors.
In addition, DISTILLER can be distributed freely for academic purposes, as long as this file and all other files mentioned in this file are distributed along with the code.
DISTILLER makes use of JCommon (http://www.jfree.org/), the original jar files of which are also included in this distribution. They should always be distributed along with DISTILLER, as well as this reference to JCommon. By using DISTILLER you agree to adhere to the terms of the GNU Lesser General Public License (LGPL) to which JCommon are subject. The GNU Lesser General Public License (LGPL) is included in this distribution, and should remain so, if further distributed:
LGPL-license.txt.
Please direct comments and questions related to the software to:
kathleen.marchal@biw.kuleuven.be
Also check the accompanying website for recent updates:
http://homes.esat.kuleuven.be/~kmarchal/Supplementary_Information_Lemmens_2008/Index.html

INSTRUCTIONS
Unpack the zip-file DISTILLER .zip and store all files in the same folder.
Make sure java 1.5 is installed.
Then:
* Execute the command 'java -jar -Xmx512m Miner.jar input.txt' in a terminal, in the folder in which the files are stored.
* Execute the command 'java -jar -Xmx512m Filter.jar inputModuleSelection.txt' for the module selection step.
PREPARING THE INPUT FILES
1. Main input file
In the main input file, one can specify the parameter settings that will be used by DISTILLER to find the seed modules. Although in our study, only expression and regulatory motif data were used, our data integration framework is very flexible for adding more data sources, for instance ChIP-chip data. These data sources can be listed by separating the parameters by commas (See the example below). The motif and/or ChIP-chip are referred to as input interaction data in the accompanying paper. Note that they can either be 0-1 matrices or matrices containing 1 – (p-values). In the latter case, the variable binary pvalues (or binary thresholds) is used to obtain the corresponding binary matrices. In contrast, all parameters concerning the expression data are referred to as 'box' parameters.
• binary supports: 3, 3
= the minimum number of regulators (in case of ChIP-chip data) or number of regulatory motifs (in case of regulatory motif data) that should be shared by the 3 genes in the modules. In this example, two input interaction matrices are available and the

parameters for these data were both set to one.

• box supports: 50

= the minimum number of arrays in which the genes of the module should be coexpressed.

• binary pvalues: 0.0001, 0.0001

= the probability that a randomly sampled gene set of a certain size (size of random modules) will satisfy the binary supports.

Alternatively, one may specify "binary thresholds: 0.99, 0.9", for example (as in the previous version). These thresholds are the minimum score (or 1 - p-value) a regulator/motif should have in the respective matrices. Based on these variables binary pvalues or binary thresholds, the algorithm converts the input interaction matrices to binary matrices.

• box pvalues: 0.0001

= the probability that a randomly sampled gene set of a certain size (size of random modules) will satisfy the threshold bandwidth sequence on the expression data (or box p-value).

• **Query-driven genelist**

= distiller will only output the modules containing at least one gene of the query-driven genelist.

• binary files: C:/Documents and Settings/data/Chip-chip.txt, C:/Documents and Settings/data/Motifs.txt

= input interaction matrices files

The binary files should be of the following format: the rows represent the genes, and for each of the respective input files the columns represent the motifs (motif data), the regulators (ChIP-chip data) or the experiments (expression data). Each binary file should contain the same number of genes, ordered in the same way. Both the rows and the columns should be numbered in the binary file.

• box files: C:/Documents and Settings/data/Expressiondata.txt

= expression data file

• number of randomizations: 1000000

= the number of random modules that will be used to set the binary and box pvalues

• size of random modules: 4

= the number of genes in the random modules (gene content threshold)

• output file initial significances: outputInitial.m

= the name of the file to which the module output should be written. This matlab file contains for each module the index of the genes (items), the arrays in which the genes are co-expressed (boxtidset1), the regulators/motifs shared by the genes (tidset1), the p-values that were assigned to the modules (Significances).

• minimal module size: 4

= the minimum gene content of the modules that should be reported

• logfile: logfile.txt

= a log file containing information on, for example, the running time

• data folder: dataFolder

= folder where all output files should be written to

2. Input file for the module selection step

During the module selection step, an iterative procedure is applied that selects the most interesting modules one by one. It takes into account the significance of individual modules but penalizes at the same time overlap with modules that have already been reported (for details, see our accompanying paper).

• output file greedy cover: outputModuleSelection.m
= name of the output file after module selection

• number of greedy modules: 100
= the number of modules that should be selected

• data folder: dataFolder
= folder where the input files for the module detection step can be found
