# Supplementary material for: An Integrated Approach to Reconstructing Genome-Scale Transcriptional Regulatory Networks
Source: PLoS Comput Biol. 2015 Feb 27;11(2):e1004103. doi: 10.1371/journal.pcbi.1004103 (PMC4344238; doi:10.1371/journal.pcbi.1004103)
Supplement: S1 Dataset — This dataset consists of 3 folders containing data used for the analysis presented in the main text and a summary of the results from the E. coli analysis. These folder include: “Ecoli _results”, which contains a summary of the results obtained from the E. coli analysis; “Integrate_v1.0”, which contains sample code for running the presented analysis (the most up-to-date version of this code can be obtained from the figshare link provided in the Materials and methods); and “Rsp_data”, which contains the R. sphaeroides gene expression compendium used in our analysis. (ZIP) [file pcbi.1004103.s024.zip › S1 Dataset/Integrate_v1.0/User Guide.doc]

**User Guide for integrative TRN inference**

**by**

**Saheed Imam**

**Institute for Systems Biology**

email: simam@systemsbiology.org

These instructions are to facilitate the use of the algorithm described in "An Integrated Approach to Reconstructing Genome-scale Transcriptional Regulatory Networks" by Saheed Imam, Daniel R. Noguera and Timothy J. Donohue, for reconstruction of TRNs using comparative genomics and gene expression analyses.

**System requirements**

The code for this workflow was written in Java and designed to work on Unix systems. It requires the following software be installed:

1. Operating system: Unix/Linux

2. Java

3. MySql

4. Meme suite

5. NCBI blast

6. Mcl

7. Perl

***Installing Java***

On Debian Linux distributions:

sudo apt-get install java-sdk

(then select one of the options provided e.g., sudo apt-get install openjdk-7-jdk)

***Installing MySql***

sudo apt-get install mysql-server

(be sure to remember the password you provide during installation as that will be required to run this application)

***Installing Meme***

Download meme suite from http://ebi.edu.au/ftp/software/MEME/index.html

Install following the instructions provided: http://meme.nbcr.net/meme/doc/meme-install.html

Add the meme bin directory to your path. This software will only work when meme has been added to your path (i.e., you can call meme from your terminal window without specifying any directory information). This can be done in several ways e.g. you can do this by editing your "~/.bashrc" files and adding your new path e.g. "export PATH=$PATH:/[path to meme bin directory].

***Installing NCBI blast***

sudo apt-get install blast2

***Installing Mcl***

sudo apt-get install mcl

***Perl***

Perl installed by default on most UNIX systems.

**Running software**

*Directories*

The home directory should have the following folders:

1. bin

2. Data

and *files*:

3. Integrate.java

4. Parameters.txt

5. Instructions.txt

*Required data*

To run the software you'll be expected to provide the appropriate data files. One of each of the following files for each organism you are interested in using for your analysis is expected:

1. **Gff v3 file** (with genomic coordinates, placed in the "Data/Gffs" directory)

2. **Genomes sequence** file (placed in the "Data/Genomes" directory)

3. **Proteome fasta** file (placed in the "Data/Proteomes" directory)

These 3 file types can be downloaded from NCBI ( ftp://ftp.ncbi.nih.gov/genomes/Bacteria ) or obtained from other sources. If obtained from other sources they should be in similar formats.

If any of the organisms you'd like to use for the analysis have multiple replicons (i.e., chromosomes or plasmids), concatenate them together with the "cat" command:

e.g., cat file1 file2 file3 > file_combined

or cat *.gff > file_combined.gff (if all gff files in the current directory belong to only one organism).

This should obviously be done separately for each type of file (ie gff or proteome or genome) and these combined files placed in the appropriate directories as described above (see the *E. coli* example data, if clarification required).

Other data files needed include:

4. **List of transcription factors** for your target organism of interest (placed in the "Data" directory in a file called “**TFs.txt**”. If this file is not provided and you intend to go beyond the comparative genomics step, you will get an error…)

5. **Gene expression matrix** (optional; if not provided analysis will stop at end comparative genomics step)

6. **GO annotaion data** (optional; see below for details)

7**. Pfam analysis of TF protein** sequences (see below for details on how to generate this)

8. **Operon data** (optional; see below for details)

**NB:** if you’re only interested in a phylogenetic footprinting analysis, then file option 5, 6, 7 and 8 are not strictly required.

Prior to running this software you'll also need to fill out the "Parameters.txt" file. This requires inputs for the following entries:

1**. Genomic_sequences** – the names of all the genome sequence fasta files you want to use for footprinting analysis i.e. the ones you placed in the "Data/Genomes" directory, or a subset thereof.

2. **GFF –** the names of all the gff files you want to use for obtaining genomic coordinates i.e. the ones you placed in the "Data/Gffs" directory, or a subset thereof.

3. **Proteomes –** the names of all the proteome fasta files you want to use for ortholog identification i.e. the ones you placed in the "Data/Proteomes" directory, or a subset thereof.

4. **Abbr –** four letter abbreviations to be used by orthomcl, which corresponds to the genome, proteome and gff files in the same order.

**NB:** the order of all the names of the files in the genomic_sequences, GFF and Proteomes entries should be the same and equivalent the order of the abbreviations used in the Abbr entry.

5. **Target –** the four letter abbreviation for your particular organism of interest. Same as the one used in Abbr.

6. **IGR_length** – the length of inter-genic region you'd like to search for motifs. Default 500. Overlap with adjacent genes will be prevented.

7. **Order** – the order of the background distribution you'd like to use. Starting from 0 which is equivalent to just the base frequency. Default 2

8. **pvalue** – pvalue for a motif match using mast. Default 0.000001 (10e^-6^).

**NB:** It may be worth trying a value of 0.0000001 (10e^-7^) and comparing the two results. For the *E. coli* network presented in the paper 10e^-7^ was used while 10e^-6^ was used for the *R. sphaeroides* network. A lower pvalue would give you more precision but reduced recall.

9. **GOA –** GO annotation file for computing gene set enrichment via hypergeometric distribution. Maybe obtainable from: ftp://ftp.ebi.ac.uk/pub/databases/GO/goa/proteomes . Alternatively a tab-separated file with gene ids (column 1) and goID (column 2) could be provide. These need not be go terms but the file must be in that format.

10. **Operon_data** – name of operon data file (operon data file for microbes can be obtained from microbes online: http://microbesonline.org/operons/OperonList.html ). If no valid file is specified, no extension will be done.

11. **Mysql credentials** - credentials for logging into Mysql (i.e. username and password)

***Running code***

Once all these are provided, open up a terminal window, navigate to the "Integrate_v1.0" directory and run:

javac Integrate.java

then

java Integrate

**NB:** During the run there will be a pause where you will be asked to provide the results for the **Pfam analysis** of the list of TFs you intend to use. To do this look into the "Data" directory and you’ll find a file called "**TF_protein_seqs.txt**", which contains the sequences for the list of TFs you provided in “TFs.txt” file. You'll need to run Pfam analysis on these sequences from ( http://pfam.xfam.org/search ). On this web page select the "Batch search" option, then choose the "TF_protein_seqs.txt" file, provide an email address and send. Copy the results (header included) from your email (when you receive it) and paste it in a text file, which you MUST name "**TF_domains.txt**". See sample *E. coli* run if you need clarification.

Only continue when this file is ready. To do this enter “y” at the command line then press ENTER.

Doing things this way seemed easier than having the user install Pfam locally, as Pfam has quite a few dependencies…

**Results**

A "Results" folder will be generated during the run with the subfolders (DISTILLER, Motif_finding and Miscellaneous) with the results from the various analysis.

One or two (if expression data is provided) HTML files summarizing the results of the analysis will created in the home directory: **Summary_phylo** and **Summary_final**

**Summary_phylo:** summary of results from phylogenetic footprinting analysis

**Summary_final**: summary of final results and predictions

Several files and generated in the Results folder and its subdirectories the most pertinent of these (all located in the Results directory) are:

1. **Integrated_modules_final.txt:** file with clusters, their associated genes and the conditions (microarrays) under which they are co-regulated (only if expression data provided).

2. **All_motifs_TOMTOM.txt:** PSSM of the motifs identified via phylogenetic footprinting in a format that can be used for comparative analysis with tools like tomtom (part of the meme software suite).

3. **Clusters.txt:** list of clusters from phylogentic footprinting and their associated genes

4. **GSEA_analysis_phylo.txt:** gene set enrichment analysis for clusters identified in phylogenetic footprinting step

5. **GSEA_analysis_file.txt:** gene set enrichment analysis for final clusters of co-regulated genes (i.e. both shared evolutionarily conserved motif and expression pattern)

6. **Final_sorted_interaction.txt:** list of interactions from the network sorted by a score generated by combining the TF R_score with the meme motif pvalue.

**NB:** At the start of each the results folder with be overwritten, so if you’re interested in keeping the results from a previous run you’ll want to move the results folder and the html files to a different folder.

***Runtime***

*E. coli* benchmark dataset (906 microarrays, 14 genomes) – ~14 hrs

*R. sphaeroides* dataset (198 microarrays, 11 genomes) – ~4 hrs

Computer specs: Intel Core i7-4770 CPU @ 3.4 GHz (8 cores), RAM 8G.

**Example dataset**

An example dataset for *E. coli* is available at <http://dx.doi.org/10.6084/m9.figshare.1249869>

**Some general comments**

Probably the most important step in this analysis is selection of the right group of organisms for phylogenetic footprinting. As a rule of thumb, I have found selecting organisms classed within the same family and/or order as the target organism to be the most productive approach. Using strains on the same organism is not very useful and may be detrimental if too many are included. On the other hand, a few different species from the same genus didn’t have a negative impact on my analysis in general.

Another point of note is the results one gets from DISTILLER. If you observe that you get a large number of clusters from the phylogenetic footprinting step (which can be accessed from the “Summary_phylo.html” file), but you only end up with a small number of clusters in the final results (“Summary_final.html”), it could mean one of two things. Either (i) your gene expression compendium is not large/diverse enough and thus DISTILLER can’t find enough biclusters; or (ii) you ran out of memory during the DISTILLER run. In the case of (ii), you’ll likely need to rerun your analysis on a computer with more RAM to enable DISTILLER to run till completion (DISTILLER required about 6G of memory for the *E. coli* data but much less for the *R. sphaeroides* analysis)

If you run into any issues or have any questions feel free to contact me (email above).

Happy hunting...
